# Brain functional network connectivity interpolation characterizes the neuropsychiatric continuum and heterogeneity

**DOI:** 10.1162/IMAG.a.1220

**Published:** 2026-04-27

**Authors:** Xinhui Li, Eloy Geenjaar, Zening Fu, Godfrey D. Pearlson, Vince D. Calhoun

**Affiliations:** Tri-institutional Center for Translational Research in Neuroimaging and Data Science, Georgia State University, Georgia Institute of Technology, Emory University, Atlanta, GA, United States; School of Electrical and Computer Engineering, Georgia Institute of Technology, Atlanta, GA, United States; Department of Psychiatry, School of Medicine, Yale University, New Haven, CT, United States

**Keywords:** functional network connectivity, psychosis continuum, psychosis heterogeneity, variational autoencoder, schizophrenia, autism spectrum disorder

## Abstract

Psychiatric and neurodevelopmental disorders such as schizophrenia (SZ) and autism spectrum disorder (ASD) are challenging to characterize in part due to their heterogeneous presentation in individuals, with symptoms now believed to exist on a continuum. Conventional diagnostic and neuroimaging analytical approaches rely on subjective assessment or group differences, but typically ignore progression between groups or heterogeneity within a group. To estimate the neuropsychiatric continuum and heterogeneity, we proposed a functional network connectivity (FNC) interpolation framework based on a variational autoencoder (VAE) using static FNC (sFNC) and dynamic FNC (dFNC) data from controls and patients with SZ or ASD. We demonstrated that VAEs significantly outperformed a linear baseline and a semi-supervised counterpart. For both sFNC and dFNC interpolation, the generated results effectively captured representative and generalizable properties in the original data. The interpolated continua from controls to patients in both SZ and ASD revealed group-wise gradients characterized by reduced positive correlations within the auditory, sensorimotor, and visual networks, as well as between the subcortical and cerebellar domains. In contrast, anti-correlations weakened between the subcortical domain and the sensory domains, and between the cerebellar domain and the sensory domains. Finally, we showed examples of how to generate continuous FNC data following group- or state-based trajectories in the VAE latent space. The proposed framework offers added advantages over traditional methods, including data-driven discovery of hidden relationships, visualization of individual differences, imputation of missing values along a continuous spectrum, and estimation of the stage where an individual falls within the continuum.

## 1 Introduction

*“Happy individuals are all alike; every unhappy individual is unhappy in their own way.”*

     *—adapted from Anna Karenina by Leo Tolstoy*

Psychiatric and neurodevelopmental disorders such as schizophrenia (SZ) and autism spectrum disorder (ASD) are recognized as the leading causes of the global disease burden, affecting hundreds of millions of individuals worldwide (GBD 2019 Mental Disorders Collaborators, 2022). SZ is a lifelong mental disorder characterized by symptoms such as delusions, hallucinations, disorganized speech, catatonic behavior, and diminished emotional expression (McCutcheon et al., 2020). ASD is a developmental disability defined by social communication difficulties and repetitive and restrictive behaviors (Lord et al., 2018). SZ and ASD show overlapping features, including genetic risk factors (O’Connell et al., 2018; The Autism Spectrum Disorders Working Group of The Psychiatric Genomics Consortium, 2017), symptom profiles (Barneveld et al., 2011; Konstantareas & Hewitt, 2001), and brain abnormalities (H. Chen et al., 2017; Du et al., 2021; Fu et al., 2021; Yoshihara et al., 2020). Moreover, both SZ and ASD are known to be highly heterogeneous across individuals (Masi et al., 2017; McCutcheon et al., 2020). Namely, individuals within the disorder group may differ in various aspects, such as genetic traits (Abrahams & Geschwind, 2008; Bill & Geschwind, 2009; Di Biase et al., 2022; Geschwind & State, 2015; Pulver et al., 2000), clinical symptoms (Ahmed et al., 2018; Georgiades et al., 2013; Kim et al., 2016; Mohr et al., 2004; Morales-Hidalgo et al., 2018), brain structure and function (Alnæs et al., 2019; Amaral et al., 2008; Brugger & Howes, 2017; Hahamy et al., 2015; Hong et al., 2017; Marín, 2012), cognitive function (Bora, 2016; Feczko et al., 2018; Joyce & Roiser, 2007), developmental trajectories (Carpenter & Kirkpatrick, 1988; Fountain et al., 2012; Lord et al., 2015), and other relevant characteristics. In addition, psychiatric symptoms, such as hallucinations and delusions, may exist along a multidimensional continuum in the general population, varying in severity, frequency, and degree of conviction (DeRosse & Karlsgodt, 2015; Johns & Van Os, 2001; Morales-Hidalgo et al., 2018; Strauss, 1969; van Os et al., 2000, 2009; Verdoux & van Os, 2002). Estimating such a continuous spectrum and imputing missing values on the continuum remain underexplored in the neuropsychiatric literature. Considering the high prevalence and severity of both disorders, as well as their similarity and complexity, it is important to develop reliable analytical methods to characterize the continuum and heterogeneity of SZ and ASD.

However, there are several limitations in conventional evaluation approaches. First, the current diagnostic system, based on the Diagnostic and Statistical Manual of Mental Disorders, Fifth Edition (DSM-5) (American Psychiatric Association, 2013), relies on expert assessment and patient self-report data. Such subjective measures are insufficient to accurately capture the biological deficits and progression patterns of mental disorders, hindering the development of more effective treatments (Craddock & Mynors-Wallis, 2014; Cuthbert & Insel, 2013; Insel et al., 2010; Scott & Nelson, 2024; Su et al., 2020). Thus, it is critical to discover objective biomarkers to characterize psychiatric and neurodevelopmental disorders. Recent neuroimaging studies have shown that SZ and ASD can be characterized by resting-state static and dynamic brain connectivity, derived from functional magnetic resonance imaging (fMRI) data (H. Chen et al., 2017; Damaraju et al., 2014; Du et al., 2021; Fu et al., 2021; Guo et al., 2019; Hahamy et al., 2015; Hull et al., 2017; Iraji et al., 2019, 2020; Yan et al., 2024; Yoshihara et al., 2020; Yu et al., 2012). Second, group-average approaches have been widely used to analyze functional connectivity patterns between patient and control cohorts (Damaraju et al., 2014; Du et al., 2020; Y. He et al., 2020; Rabany et al., 2019). Yet, given the heterogeneous nature of SZ and ASD, group-level summaries alone are not sufficient to characterize their multidimensional continua or multifaceted heterogeneity (Segal et al., 2025). Third, supervised learning methods, which utilize diagnostic labels, have been widely developed to classify disorder conditions from functional connectivity data (C. P. Chen et al., 2015; Rabany et al., 2019; Rashid et al., 2016; Zeng et al., 2018). As a result, the success of supervised learning models inherently depends on the validity of diagnostic labels. However, these diagnostic labels derived from current psychiatric nosologies, in addition to their subjectivity, indicate only the current state of a disorder and cannot predict future progression for early intervention (Scott & Nelson, 2024). Additionally, binary or multi-class supervised classification approaches alone neither explain individual differences within a group nor estimate psychotic continua between groups. To address these challenges, it is necessary to apply *unsupervised* learning approaches to brain imaging data to characterize objective biomarkers, individual variability, and psychotic continua.

Variational autoencoders (VAEs) (Kingma & Welling, 2014) are a class of generative models that can learn data distributions in an *unsupervised* manner. The VAE architecture consists of an encoder and a decoder. The encoder projects input data onto a latent space, and the decoder reconstructs the original input data from the learned latent distributions. There are two key benefits of using the VAE. First, the encoder can perform *nonlinear* dimension reduction and approximate low-dimensional latent distributions from high-dimensional data. Second, the decoder can generate continuous synthetic data that closely resemble the observed data by sampling from the learned distributions. Recent studies have demonstrated that latent representations of resting-state fMRI data learned by a VAE can be used to identify SZ (Geenjaar et al., 2021) and ASD (F. Zhang et al., 2022), as well as characterize spatiotemporal dynamics (X. Zhang et al., 2021). Traditional dimension reduction approaches such as principal component analysis (PCA) (Jolliffe, 1986) and independent component analysis (ICA) (Hyvärinen & Oja, 2000) cannot naturally handle missing values, and thus cannot infer a continuous spectrum from the data. Probabilistic PCA (PPCA) has been developed to address the limitation of PCA by estimating a probabilistic model of observed data (Tipping & Bishop, 1999), but it cannot capture complex nonlinear relationships due to its linear nature. Deep learning models such as autoencoders nonlinearly compress the data into latent features but do not provide a probabilistic estimate of the data continuum. Other deep generative models also have limitations in this specific task. For example, generative adversarial networks (GANs) (Goodfellow et al., 2014) are known for mode collapse (Che et al., 2017; Salimans et al., 2016) and instability issues (Barnett, 2018). Furthermore, diffusion models (Ho et al., 2020) learn latent variables of the same dimensionality as the original data, rather than learning a low-dimensional, interpretable latent space. In addition, inference in diffusion models is computationally intensive, which typically requires hundreds of denoising steps. In contrast, the VAE provides a low-dimensional manifold that allows effective *interpretation* and efficient *interpolation* of latent features, making it a promising architecture for characterizing the neuropsychiatric continuum and individual variability.

Here, we propose an interpretable and generative unsupervised learning framework based on a VAE to interpolate within a latent space learned from static functional network connectivity (sFNC) or dynamic functional network connectivity (dFNC) derived from resting-state fMRI. Interpolation—mathematically defined as the process of estimating an unknown value based on surrounding known values—can be applied to estimate an FNC matrix from neighboring matrices, assuming continuous changes between observed samples. For sFNC interpolation, a trained VAE was used to interpolate the sFNC matrices by sampling from the latent distributions at equal intervals on a 2-dimensional (2D) grid. The original sFNC matrices were also displayed on the 2D grid based on their similarity. For dFNC interpolation, we trained a VAE using all windowed dFNC matrices from the training subjects, performed k-means clustering (Hartigan & Wong, 1979) on the VAE latent features, and then generated dynamic states based on the k-means clusters. For comparison, dynamic states were also identified by applying k-means clustering directly to the dFNC matrices.

Our study highlights the benefits of maximizing data transparency to visualize individual differences within a group and continuous changes between groups. Crucially, sFNC interpolation facilitates examining individual variability and disorder continua, while dFNC interpolation captures generalizable group-specific dynamic states and state transition patterns, providing both static and dynamic comprehensive views of mental illnesses. Moreover, the VAE latent space serves as a low-dimensional manifold for interpolating and interpreting sFNC and dFNC matrices, offering insights into FNC changes across different subjects, diagnostic groups, and dynamic states.

## 2 Methods

### 2.1 Data

#### 2.1.1 Datasets

We used two resting-state fMRI datasets: the Functional Biomedical Informatics Research Network (FBIRN) dataset (Keator et al., 2016) and the initial release of the Autism Brain Imaging Data Exchange (ABIDE I) dataset (Di Martino et al., 2014).

The FBIRN fMRI scans were acquired using a standard gradient-echo echo-planar imaging (EPI) paradigm with repetition time (TR) = 2s, echo time (TE) = 30 ms, flip angle (FA) = $77^{\circ }$, 162 volumes, 32 sequential ascending axial slices of 4mm thickness and 1mm skip. Participants had their eyes closed during the scan. In addition to the fMRI data, we utilized the Positive and Negative Syndrome Scale (PANSS) scores (Kay et al., 1987), with positive and negative subscale scores summed separately. We also used six cognitive domain scores (speed of processing, attention/vigilance, working memory, verbal learning, visual learning, and reasoning/problem solving), as well as composite scores derived from the Computerized Multiphasic Interactive Neurocognitive System (CMINDS) (van Erp et al., 2015). All CMINDS cognitive scores were z-scored based on the mean and standard deviation of the control group (CTR). The original FBIRN dataset includes 311 subjects. A total of 45 subjects were excluded due to missing or invalid diagnostic or cognitive scores. The remaining 266 subjects were used in the subsequent analysis, including 125 subjects labeled as SZ (age mean ± s.d.: 38.88±11.19 years; 95 males, 30 females) and 141 controls (age mean ± s.d.: 37.15±11.03 years; 103 males, 38 females).

The original ABIDE I dataset includes 869 subjects. We excluded 107 subjects without the Autism Diagnostic Observation Schedule (ADOS) scores (Lord et al., 2000). From the remaining cohort, we selected 266 subjects with valid ADOS scores from the 3 sites with the highest sample sizes, including 133 subjects labeled as ASD (age mean ± s.d.: 17.01±7.20 years; 119 males, 14 females) and 133 controls (age mean ± s.d.: 16.05±5.67 years; 104 males, 29 females). The site-specific demographics and acquisition parameters for the ABIDE I participants are presented in Tables S1 and S2, respectively (Supplementary Material, Section 1).

For each dataset, 85% of subjects were used as the training set ($N_{\text{train}}=225$ ) and 15% of subjects with balanced labels were used as the holdout test set ($N_{\text{test}}=41$ ). The detailed demographic information of the subjects used in this study is described in Table 1. The statistics of subject measures in each dataset are shown in Table S3 (Supplementary Material, Section 2).

**Table 1.** Dataset demographic information.

Dataset	Label	N	$N_{\text{male}}$	$N_{\text{female}}$	$N_{\text{train}}$	$N_{\text{test}}$	Age mean ± s.d. (years)	Age range (years)
FBIRN	SZ	125	95	30	105	20	38.88±11.19	18−62
FBIRN	CTR	141	103	38	120	21	37.15±11.03	19−59
ABIDE I	ASD	133	119	14	113	20	17.01±7.20	7−39
ABIDE I	CTR	133	104	29	112	21	16.05±5.67	7−32

Abbreviations: N, number of total subjects; $N_{male}$, number of male subjects; $N_{female}$, number of female subjects; $N_{train}$, number of subjects in the training set; $N_{test}$, number of subjects in the test set; s.d., standard deviation.

#### 2.1.2 Data preprocessing

The fMRI data were preprocessed using the Statistical Parametric Mapping toolbox (SPM12, http://www.fil.ion.ucl.ac.uk/spm/) (Ashburner et al., 2021) in the MATLAB 2016 environment. The first five scans were discarded to allow for signal equilibrium and participant adaptation to the scanner environment. Rigid-body motion correction was then performed using SPM, followed by slice timing correction. Subsequently, the fMRI data were registered to the standard Montreal Neurological Institute (MNI) space using an EPI template and resampled to 3×3×3 mm^3^ isotropic voxels. The resampled fMRI data were further smoothed using a 6-mm full width at half maximum (FWHM) Gaussian kernel.

#### 2.1.3 NeuroMark functional network connectivity

The fully automated NeuroMark pipeline (Du et al., 2020) was applied to the preprocessed fMRI data to extract subject-specific functional components and their corresponding time courses (TCs). NeuroMark, implemented in the Group ICA of fMRI Toolbox (GIFT; http://trendscenter.org/software/gift), leverages network templates (available in GIFT and at http://trendscenter.org/data) originally derived by computing group-level independent components (ICs) separately from two independent datasets of healthy individuals: the Human Connectome Project (HCP) (Van Essen et al., 2013) and the Brain Genomics Superstruct Project (GSP) (Holmes et al., 2015). ICs from the 2 datasets were matched based on spatial correlations, and 53 intrinsic connectivity networks (ICNs) with correlations greater than 0.4 were selected as the network templates. The less noisy ICNs from the GSP dataset were selected for inclusion in the NeuroMark_fMRI_1.0 template (Du et al., 2020). These 53 ICNs were then ordered and assigned to 7 functional domains according to their anatomical and functional properties, including the subcortical (SC), auditory (AU), sensorimotor (SM), visual (VI), cognitive control (CC), default mode (DM), and cerebellar (CB) domains (Allen et al., 2012; Du et al., 2020). Using the components in this template as spatial priors, we performed spatially constrained ICA to derive ICNs and TCs for each subject in the FBIRN and ABIDE I datasets. The NeuroMark framework offers three key advantages: (1) fully automating the ICA pipeline, (2) estimating ICNs separately for each subject to prevent data leakage, and (3) estimating aligned, comparable ICNs across both datasets. The extracted TCs underwent four postprocessing steps: detrending, head motion regression, despiking, and low-pass filtering (0.15 Hz).

Static functional network connectivity (sFNC) was subsequently calculated as the pairwise Pearson correlation between the TCs of the 53 ICNs, yielding a 53×53 matrix. Dynamic functional network connectivity (dFNC) was estimated using a sliding window approach and a graphical LASSO method (Friedman et al., 2007). A tapered window was used to segment the TCs by convolving a rectangle (width =20 TRs for FBIRN and 26 TRs for ABIDE I^1^) with a Gaussian kernel (σ=3 TRs). With a step size of 1 TR, this process yielded 137 windows in the FBIRN dFNC data and 168 windows in the ABIDE I dFNC data.

### 2.2 Variational autoencoders

We used an unsupervised generative model, a variational autoencoder (VAE) (Kingma & Welling, 2014), as the backbone of the interpolation framework. We trained one VAE for each dataset (FBIRN or ABIDE I) and each data type (sFNC or dFNC). Here, we briefly explain the VAE objective function and provide a step-by-step derivation in Supplementary Material, Section 3.

Let $D_{\text{sFNC}}=\left\{{\text{x}}^{\left(1\right)},\ldots ,{\text{x}}^{\left(i\right)},\ldots ,{\text{x}}^{\left(N\right)}\right\}$ denote a dataset of N sFNC samples ${\text{x}}^{\left(i\right)}\in {\mathbb{R}}^{V}$, where N is the number of subjects and V is the number of features in each sample. Each sample ${\text{x}}^{\left(i\right)}$ is the vectorized upper triangle of a symmetric sFNC matrix, thus the dimensionality of each sample can be calculated as $V=\frac{1}{2}\times \left(53\times 53-53\right)=1378$ . For dFNC data, we treat each windowed dFNC matrix as an independent sample and denote the dFNC dataset as $D_{\text{dFNC}}=\left\{{\text{x}}^{\left(1,1\right)},\ldots ,{\text{x}}^{\left(i,t\right)},\ldots ,{\text{x}}^{\left(N,T\right)}\right\}$. The number of total samples is the product of the number of subjects N and the number of windows T (N×T ). Each sample ${\text{x}}^{\left(i,t\right)}\in {\mathbb{R}}^{V}$ is the flattened upper triangle of a windowed dFNC matrix from the i-th subject and the t-th window (V=1378 ).

For pedagogical purposes, we explain the generative model using a single sample $\text{x}\in {\mathbb{R}}^{V}$. Let $\text{z}\in {\mathbb{R}}^{D}$ denote a continuous latent variable that represents the underlying factors in the input data. We define a 2-dimensional latent space (i.e., D=2 ) to better visualize the FNC matrices. Note that it is also possible to define a latent space with a different dimension. We assume that the latent variable z is sampled from a Gaussian prior distribution p(z)=N(z|0,I) and the observed data variable x is sampled from the conditional likelihood distribution $p_{\theta }(\text{x}|\text{z})$ parameterized by θ, with the joint distribution $p_{\theta }\left(\text{x},\text{z}\right)=p_{\theta }(\text{x}|\text{z})p\left(\text{z}\right)$. Our goal is to estimate the posterior distribution to perform inference on the latent variable. According to Bayes’ theorem, the posterior can be derived as

$$p_{\theta }(\text{z}|\text{x})=\frac{p_{\theta }\left(\text{x},\text{z}\right)}{p_{\theta }\left(\text{x}\right)}=\frac{p_{\theta }(\text{x}|\text{z})p\left(\text{z}\right)}{p_{\theta }\left(\text{x}\right)}=\frac{p_{\theta }(\text{x}|\text{z})p\left(\text{z}\right)}{\int p_{\theta }(\text{x}|\text{z})p\left(\text{z}\right)\text{d}\text{z}}.$$ (1)

However, the computation of the posterior $p_{\theta }(\text{z}|\text{x})$ is analytically intractable because the integral of the marginal likelihood $\int p_{\theta }(\text{x}|\text{z})p\left(\text{z}\right)\text{d}\text{z}$ cannot be evaluated in closed form. Hence, we perform *variational inference* to approximate the true posterior distribution with a simpler distribution.

Here, we approximate the posterior distribution using a multivariate Gaussian distribution with diagonal covariance, $q_{\phi }(\text{z}|\text{x})=N(\text{z}|\mu_{\phi }\left(\text{x}\right),\mathrm{diag}\;\text{(}\sigma_{\phi }^{2}\left(\text{x}\right)))$ . The Kullback–Leibler (KL) divergence is used to quantify the difference between the true posterior distribution $p_{\theta }(\text{z}|\text{x})$ parameterized by θ and the multivariate Gaussian approximation $q_{\phi }(\text{z}|\text{x})$ parameterized by ϕ. The objective function, also known as the *evidence lower bound (ELBO)*, aims to maximize the negative KL divergence:

$$\begin{array}{l} \begin{array}{ll} {ℒ}_{\text{ELBO}} & =\underset{\theta ,\phi }{\text{max}}-D_{\text{KL}}(q_{\phi }(\text{z}|\text{x})∥p_{\theta }(\text{z}|\text{x}))\\ & \equiv \underset{\theta ,\phi }{\text{max}}-D_{\text{KL}}(q_{\phi }(\text{z}|\text{x})∥p_{\theta }(\text{x}|\text{z})p\left(\text{z}\right))\\ & =\underset{\theta ,\phi }{\text{max}}E_{q_{\phi }(z|x)}[\text{log}p_{\theta }(\text{x}|\text{z})]-D_{\text{KL}}(q_{\phi }(\text{z}|\text{x})∥p\left(\text{z}\right)). \end{array} \end{array}$$ (2)

Note that the KL divergence is non-negative, that is, $D_{\text{KL}}\left(\cdot \right)\geq 0$ . Thus, the ELBO is the lower bound to the log likelihood of generating the observed data, that is, ${ℒ}_{\text{ELBO}}\leq \text{log}p_{\theta }\left(\text{x}\right)$. An encoder parameterized by the variational parameters ϕ (i.e., $q_{\phi }(\text{z}|\text{x})$ ) and a decoder parameterized by the generative parameters θ (i.e., $p_{\theta }(\text{x}|\text{z})$ ) are trained to maximize the ELBO, thereby simultaneously maximizing the log likelihood of generating the observed data samples and minimizing the KL divergence between the approximate posterior and the true posterior. Maximizing the ELBO is equivalent to minimizing the objective function as follows (see Supplementary Material, Section 3 for more details):

$${ℒ}_{\text{VAE}}=\underset{\theta ,\phi }{\text{min}}\frac{1}{V}\sum_{i=1}^{V}{\left(x_{i}-{\hat{x}}_{i}\right)}^{2}-\frac{1}{2}\sum_{j=1}^{D}\left(1+\text{log}\sigma_{j}^{2}-\mu_{j}^{2}-\sigma_{j}^{2}\right),$$ (3)

where $x_{i}$ and ${\hat{x}}_{i}$ are the i-th element of the observed data x and the reconstructed data $\hat{\text{x}}$, respectively; $\mu_{j}$ and $\sigma_{j}$ are the j-th element of $\mu_{\phi }\left(\text{x}\right)$ and $\sigma_{\phi }\left(\text{x}\right)$, respectively. To train the model using gradient-based optimization, the *reparameterization trick* (Kingma & Welling, 2014) is used: $\text{z}=\mu_{\phi }\left(\text{x}\right)+\sigma_{\phi }\left(\text{x}\right)⊙\epsilon$ , where ⊙ represents the element-wise product, and $\epsilon \in {\mathbb{R}}^{D}$ is a noise variable ϵ∼N(0,I). By optimizing the objective function ${ℒ}_{\text{VAE}}$ , the VAE is capable of learning the underlying factors in the dataset and generating continuous synthetic data by interpolating between latent samples using the learned factors.

A multilayer perceptron (MLP) was implemented as the encoder and decoder in the VAE. We performed a hyperparameter search to select the optimal model architecture that yielded the best training performance from four different architectures for each dataset and each data type separately (see Supplementary Material, Section 6.1, Tables S4 and S5, Figure S1). The model was trained using the Adam optimizer (Kingma & Ba, 2015) with an initial learning rate of 0.001 . We implemented a learning rate scheduler to reduce the learning rate by a factor of 0.1 if the loss value did not improve for 10 epochs. Each model was trained for 1000 epochs with a batch size of 16 and 512 for the sFNC and dFNC data, respectively. We implemented an early stopping mechanism to avoid overfitting: if the loss value did not decrease after 20 epochs, the training would stop. All training processes stopped within the predefined number of epochs. For each model, we initialized the model weights with 10 different random seeds and recorded the results across 10 runs. The model was implemented in Python using the PyTorch framework (Paszke et al., 2019) and trained with NVIDIA A40 GPUs on a high-performance computing (HPC) cluster.

### 2.3 Model comparison

To comprehensively evaluate model performance, we compared the VAE with (1) a linear baseline method—probabilistic principal component analysis (PPCA) (Tipping & Bishop, 1999) and (2) a semi-supervised alternative—identifiable variational autoencoder (iVAE) (Khemakhem et al., 2020). We review the generative model and optimization approach used in PPCA in Supplementary Material, Section 4, and describe the generative model and objective function for iVAE in Supplementary Material, Section 5.

#### 2.3.1 Probabilistic principal component analysis

PPCA is a probabilistic extension of PCA. Unlike conventional PCA, PPCA can estimate missing values in the dataset and generate samples from the learned distribution. Recent work has shown that the principal components learned by PPCA can be fully recovered by linear VAEs (Lucas et al., 2019). Thus, PPCA can be viewed as equivalent to linear VAEs. We implemented the expectation-maximization (EM) algorithm to estimate the model parameters, as described in Tipping and Bishop (1999). We randomly initialized the model parameters with 10 random seeds and reported the results across 10 runs.

#### 2.3.2 Identifiable variational autoencoders

Although a VAE is trained to learn latent distributions, there is no guarantee of identifiability^2^ of the model parameters in general. The iVAE has been developed to address the identifiability problem by conditioning the latent variable on an auxiliary variable (e.g., class label, time index, or other observation), recovering the conditionally identifiable latent variables up to a permutation and pointwise nonlinear transformation (Khemakhem et al., 2020). Since our main interest lies in neuropsychiatric characteristics, we used the one-hot encoded diagnostic label as the auxiliary variable in the iVAE. We conducted a similar hyperparameter search experiment to select the optimal iVAE architecture (Supplementary Material, Section 6.2, Figure S2). Each iVAE model was initialized using 10 different random seeds.

### 2.4 Functional network connectivity interpolation framework

**Fig. 1.**
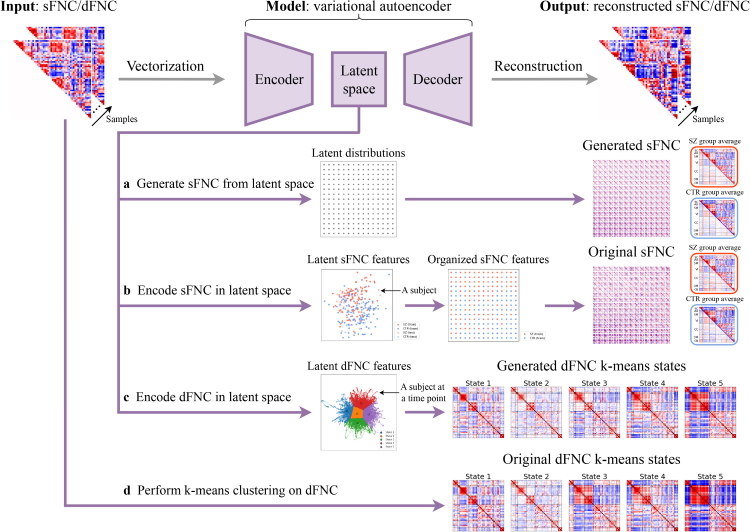
Overview of functional network connectivity interpolation framework. The upper triangle of each sFNC or windowed dFNC matrix was vectorized and used as input to the VAE. During the training stage (gray arrows), a VAE was trained to learn a 2D latent variable z by minimizing the reconstruction loss between the input and the reconstructed output, while a KL divergence term regularized the difference between the prior distribution p(z) and the approximate posterior distribution $q_{\phi }(\text{z}|\text{x})$ . During inference time, four pathways (purple arrows) are described as follows: (a) sFNC matrices were generated by sampling from evenly spaced coordinates in the latent space and then forward propagating the samples through the trained decoder. (b) Original sFNC matrices were projected onto the latent space by the trained encoder, the corresponding latent features were mapped to a 2D grid using the JV algorithm, and the corresponding original sFNC matrices were then displayed on the 2D grid. (c) dFNC matrices were projected onto the latent space by the trained encoder. The k-means clustering algorithm was then applied to the dFNC latent features to identify clusters. We then generated dFNC matrices by sampling from the posterior distributions $q_{\phi }(\text{z}|\text{x})$ . The generated dFNC state was then computed as the element-wise median of the generated dFNC matrices in each cluster. (d) The k-means clustering algorithm was performed on the original dFNC matrices directly. The original dFNC state was then computed as the element-wise median of the original dFNC matrices in each cluster.

We propose a functional network connectivity interpolation framework based on a VAE^3^ (Fig. 1). For each dataset, independent VAE models were trained for the sFNC and dFNC data, respectively. The vectorized upper triangle of each sFNC or windowed dFNC matrix was used as input to the VAE. The VAE was trained to learn a latent variable z of the training data by minimizing the reconstruction loss between the input and the reconstructed output, while a KL divergence term regularized the difference between the prior distribution p(z) and the approximate posterior distribution $q_{\phi }(\text{z}|\text{x})$ . After the training stage, we performed sFNC interpolation and dFNC interpolation separately, as described below.

#### 2.4.1 Static functional network connectivity interpolation

We independently passed the training and test sFNC matrices through the trained encoder to extract the corresponding 2-dimensional features in the latent space. To generate synthetic data, we defined a 15×15 grid of evenly spaced 2D coordinates in the latent space (as shown in Fig. 1a), with the range determined by the 80 th percentile of the absolute values of the latent features from the training set. The grid size was chosen so that the total number of grid points (i.e., the square of the grid’s side length) approximates the number of training samples, allowing all training subjects’ sFNC matrices to be displayed on the 2D grid. These coordinates (values of latent variables) were then forward propagated through the trained decoder to generate continuous synthetic sFNC matrices, which were visualized on the 2D grid. To examine the corresponding original data, we applied the Jonker–Volgenant (JV) algorithm (Jonker & Volgenant, 2005) for the linear sum assignment problem to map these latent features to the 2D grid nodes by minimizing the pairwise Euclidean distance between the positions of the latent features and the grid nodes. The original sFNC matrices were then displayed at their corresponding grid positions, allowing side-by-side visualization of the generated and original sFNC matrices on the 2D grid.

#### 2.4.2 Dynamic functional network connectivity interpolation

We independently projected the training and test windowed dFNC matrices onto the VAE latent space and extracted their corresponding latent features. Subsequently, the k-means clustering algorithm was applied to these dFNC latent features of both patient and control groups, with the number of states k searched from 2 to 9 using a fixed random seed. The optimal number of states was identified according to the elbow criterion applied to the mean $L_{2}$ distance between the samples and their respective centroids. The dFNC matrices were generated by sampling from the learned posterior distributions $q_{\phi }(\text{z}|\text{x})$ . The group-specific generated dFNC state was then computed as the element-wise median of the generated dFNC matrices in each cluster for each group. For comparison, the k-means clustering algorithm was applied to the original dFNC matrices directly over the same range of k, and then the optimal value was identified using the same elbow criterion. The group-specific original dFNC state was then computed as the element-wise median of the original dFNC matrices in each cluster for each group.

To further quantify the properties of the dFNC states, we calculated the group-specific dwell time (DT) for each state, defined as the number of FNC matrices assigned to state α ($N_{{\text{FNC}}_{g}}^{\alpha }$) divided by the number of subjects in that state ($N_{g}^{\alpha }$) for each group g:

$${\text{DT}}_{g}^{\alpha }=\frac{N_{{\text{FNC}}_{g}}^{\alpha }}{N_{g}^{\alpha }}.$$ (4)

We also calculated the group-specific transition probability matrix ${\text{P}}_{g}\in {\mathbb{R}}^{k\times k}$ to evaluate how likely a state changes to another between two consecutive time points. Specifically, we counted the number of transitions from state α to state β throughout the time course T for each group g:

$$N_{g}^{\alpha \to \beta }=\frac{1}{N_{g}}\sum_{i\in G}\frac{1}{T-1}\sum_{t=1}^{T-1}\delta \left(S\left({\text{x}}^{\left(i,t\right)}\right)=\alpha ,S\left({\text{x}}^{\left(i,t+1\right)}\right)=\beta \right),$$ (5)

where δ(⋅)=1 when the transition occurs from state α at time t to state β at time t+1 for the i-th subject, otherwise δ(⋅)=0 . S(⋅)∈{1,…,k} indicates the state of a sample. G is the set of subject indices for a group and $N_{g}$ is the corresponding number of subjects for that group.

The off-diagonal entry of the group-specific transition probability matrix ${\text{P}}_{g}\left(\alpha ,\beta \right)$ (α≠β ) is defined as the number of transitions from state α to state β divided by the total number of transitions from state α to all the other states:

$$\begin{array}{c} {\text{P}}_{g}\left(\alpha ,\beta \right)=P\left(S\left({\text{x}}^{\left(i,t+1\right)}\right)=\beta \text{|}S\left({\text{x}}^{\left(i,t\right)}\right)=\alpha ,i\in G\right)\\ =\frac{N_{g}^{\alpha \to \beta }}{\sum_{\gamma \in \left\{1,\ldots ,k\right\}\backslash \left\{\alpha \right\}}N_{g}^{\alpha \to \gamma }}. \end{array}$$ (6)

## 3 Results

### 3.1 sFNC interpolation captures the psychosis continuum and heterogeneity

**Fig. 2.**
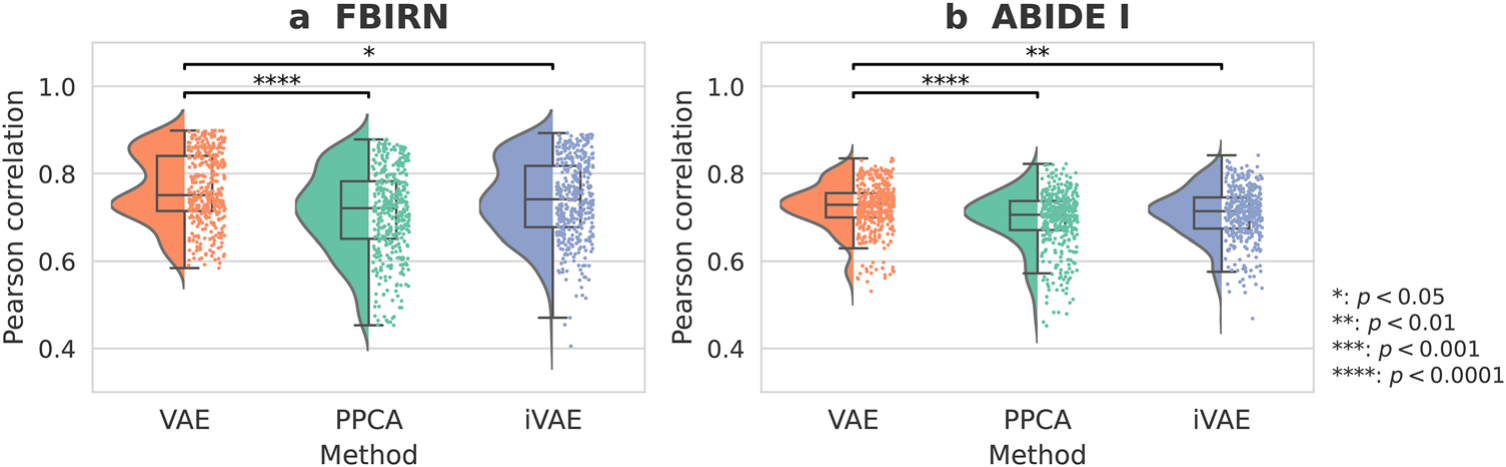
Pearson correlations between generated and original sFNC matrices. (a) FBIRN test data. (b) ABIDE I test data. The scatter-box-violin plot shows the data distribution, median and interquartile range of Pearson correlations between original and generated sFNC matrices for the VAE, PPCA, and iVAE across 10 random seeds. VAEs significantly outperformed iVAEs and PPCA in both datasets (Wilcoxon signed-rank test, Bonferroni correction).

We compared VAEs with two alternative methods, probabilistic principal component analysis (PPCA) and identifiable variational autoencoders (iVAEs). We hypothesized that nonlinear VAEs, which capture more complex relationships from observed data, would achieve better performance than linear PPCA. As expected, Pearson correlations between generated and original sFNC matrices from VAEs were significantly higher than those from PPCA (p<0.0001 for FBIRN and ABIDE I, Wilcoxon signed-rank test, Bonferroni correction; Fig. 2). More specifically, the mean correlations for VAEs were 0.765 and 0.724 , while those for PPCA were 0.711 and 0.694 for FBIRN and ABIDE I, respectively. Surprisingly, VAEs also significantly outperformed iVAEs in this interpolation task (p<0.05 for FBIRN, p<0.01 for ABIDE I, Wilcoxon signed-rank test, Bonferroni correction; Fig. 2). The mean correlations for iVAEs were 0.741 for FBIRN and 0.708 for ABIDE I, both slightly lower than the corresponding values for VAEs. By design, VAEs are better suited for learning low-dimensional continuous representations in this case due to their *nonlinear* and *unsupervised* nature. Our empirical results confirmed this theoretical advantage, as VAEs significantly outperformed both a linear baseline (PPCA) and a semi-supervised alternative (iVAE). Therefore, we focused on VAEs for all subsequent experiments.

**Fig. 3.**
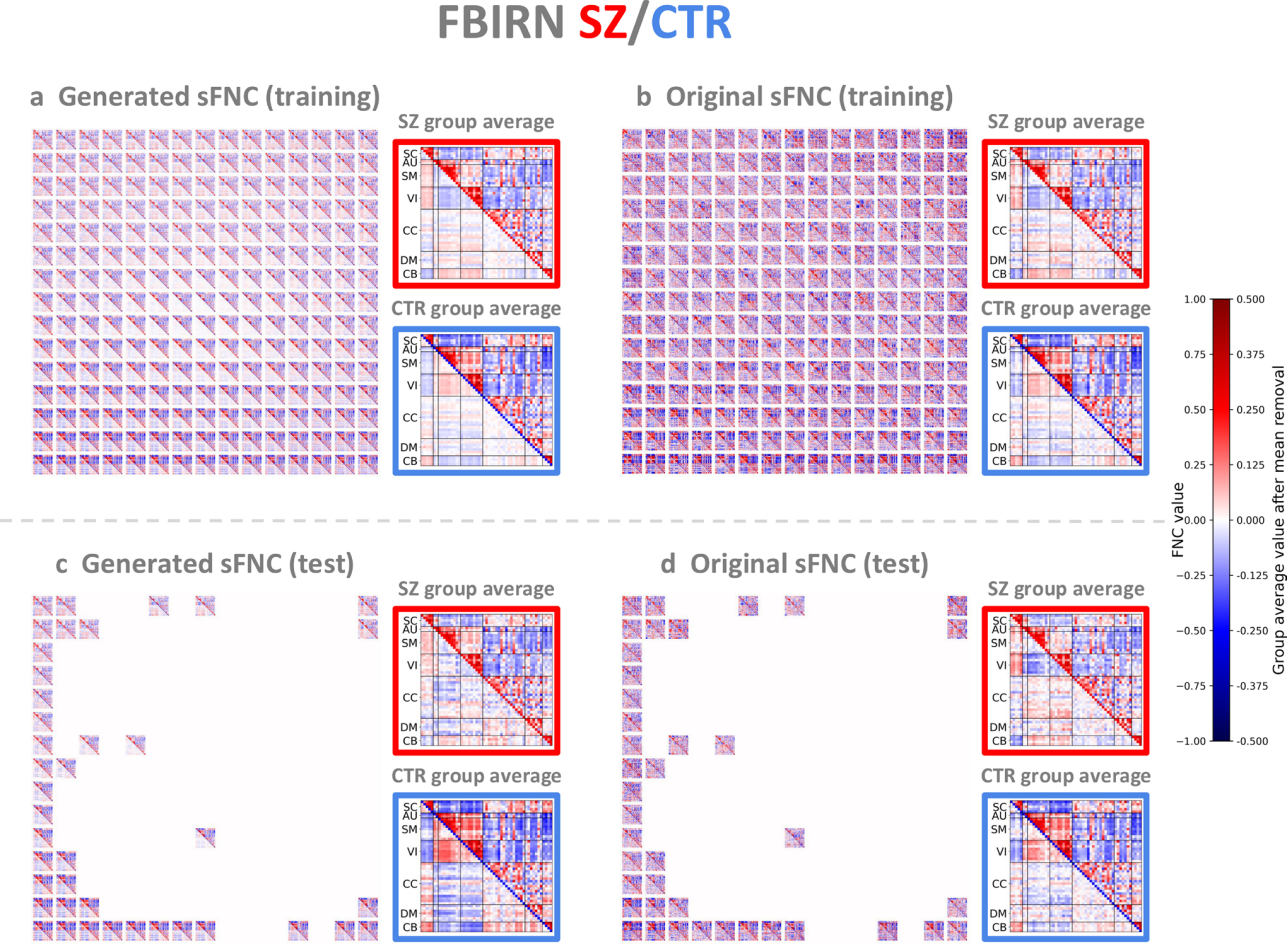
Generated and original FBIRN sFNC matrices. (a) Generated sFNC matrices in the training set. (b) Original sFNC matrices in the training set. (c) Generated sFNC matrices in the test set. (d) Original sFNC matrices in the test set. For each sFNC matrix, we retained the upper triangle, removed the element-wise mean across all subjects in the lower triangle to show how each subject deviates from the population mean, and color coded the diagonal based on diagnostic groups (red: patients; blue: controls). In each red or blue box, the SZ patient or control group-average pattern is shown in the upper triangle, and the corresponding pattern after removing the element-wise mean across all subjects is shown in the lower triangle (values were multiplied by 2 for better visualization). The generated sFNC matrices showed a high degree of correspondence with the original matrices. The generated sFNC matrices captured continuous alterations between groups, while the original sFNC matrices showed individual variability across subjects.

**Fig. 4.**
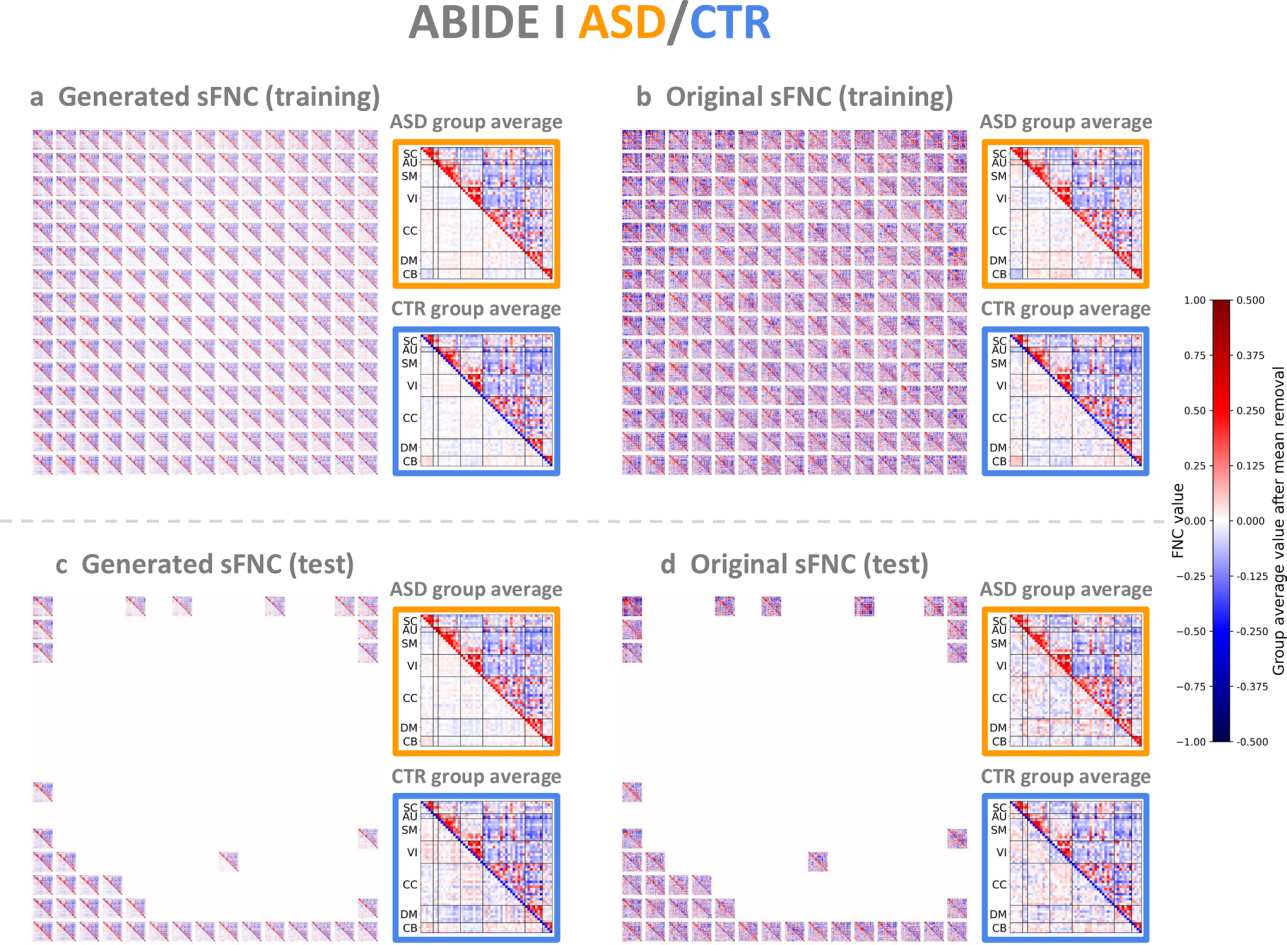
Generated and original ABIDE I sFNC matrices. (a) Generated sFNC matrices in the training set. (b) Original sFNC matrices in the training set. (c) Generated sFNC matrices in the test set. (d) Original sFNC matrices in the test set. In each orange or blue box, the ASD or control group-average pattern is shown in the upper triangle, and the respective group-average pattern after removing the element-wise mean across all subjects is shown in the lower triangle (values were multiplied by 2 for better visualization).

We confirmed that the VAE representations were robust to potential confounding factors, including age, sex, and collection site (Supplementary Material, Section 7, Figure S3), and subsequently performed sFNC interpolation as described in Section 2.4.1. The generated sFNC matrices showed a high degree of correspondence with the original matrices for both datasets (Figs. 3 and 4; zoom-in version in Supplementary Material, Section 8, Figures S4-S11). Group-specific patterns, group pattern alterations, and individual differences can be visualized on the 2D grid. By examining the group-specific patterns in the generated and original sFNC matrices, we observed focal modularity in the top half dominated by the patient group, and highly modular and polar patterns in the bottom half dominated by the control group. Specifically, the SZ patient group showed weaker functional connectivity within and between the auditory (AU), sensorimotor (SM), and visual (VI) domains, as well as between the subcortical (SC) and cerebellar (CB) domains, compared with the control group (Fig. 3). Similarly, the ASD patient group also showed weaker connectivity in these same domains (Fig. 4). In each dataset, the average element-wise standard deviation of the patient group was marginally higher than that of the control group, suggesting greater variability in the patient group (Supplementary Material, Section 9, Figure S12). In addition, the generated sFNC matrices revealed continuous alterations between diagnostic groups. The continuous sFNC spectrum from the SZ patient group to the control group showed two key connectivity patterns: (1) stronger positive correlations within the sensory domains (AU, SM, VI) and between the SC and CB domains, and (2) stronger negative correlations between the SC domain and the sensory networks, as well as between the CB domain and these sensory domains. We observed a similar continuum of sFNC changes from ASD patients to controls, but the group differences were less pronounced than those between SZ patients and controls, suggesting that functional connectivity alterations in ASD may not be as substantial as those in SZ. Overall, the generated sFNC matrices showed representative patterns within a group and continuous alterations between groups, while the original sFNC matrices ordered on the 2D grid revealed subject-level variability and inter-subject relationships.

**Fig. 5.**
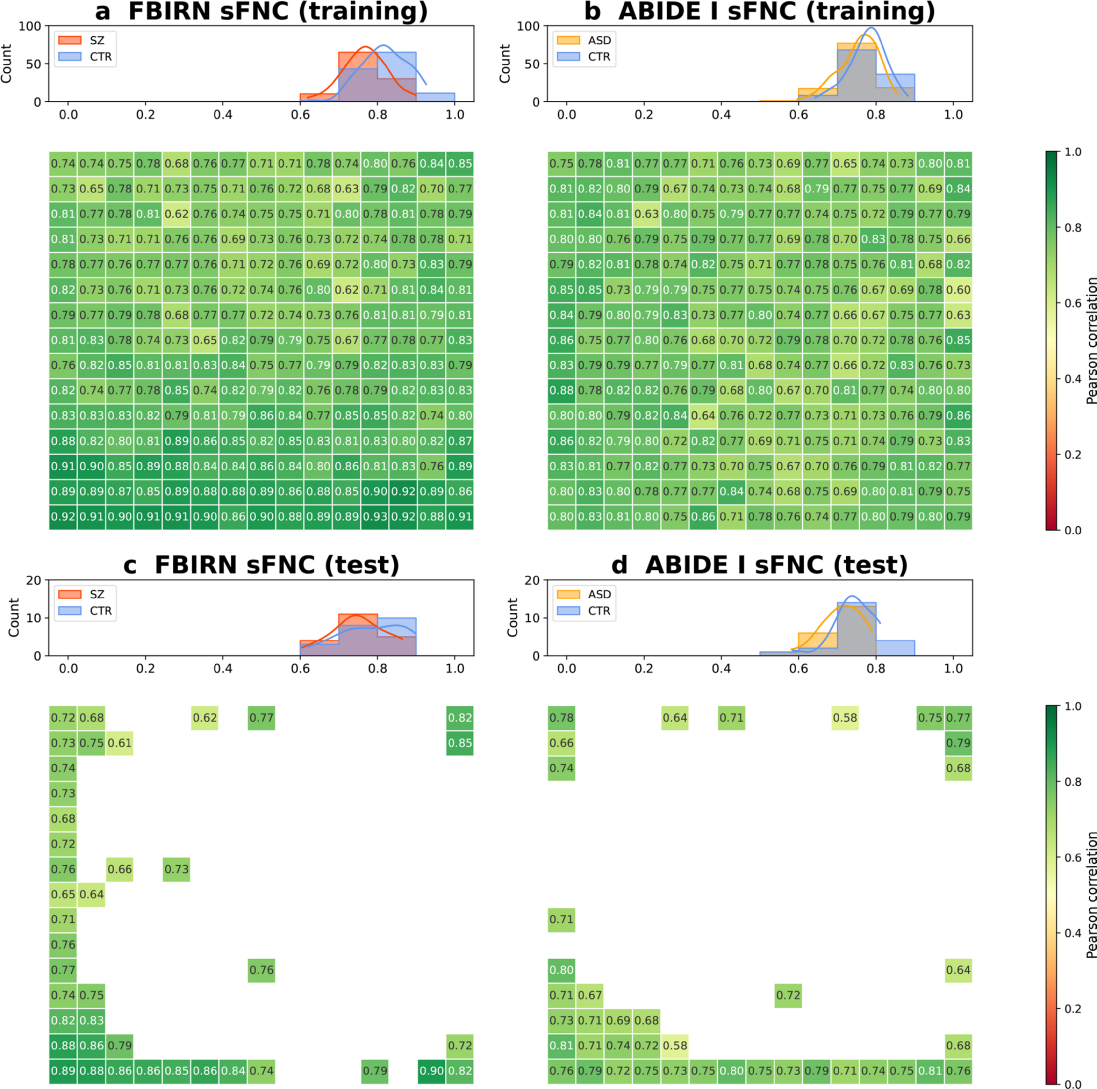
Pearson correlations between generated and original sFNC matrices. (a) FBIRN training set. (b) ABIDE I training set. (c) FBIRN test set. (d) ABIDE I test set. Each panel shows Pearson correlations between individual generated and original sFNC matrices on the 2D grid and the corresponding group-specific histogram distributions. High correlations across all panels suggest that the generated sFNC matrices closely matched the original sFNC matrices ordered by the JV algorithm, especially for the control groups.

In addition, we measured Pearson correlations between generated and original sFNC matrices to quantify their correspondence (Fig. 5). For the FBIRN training set, all 225 matrices (100.0% ) had correlations greater than 0.6 and 106 out of 225 matrices (47.1% ) had correlations greater than 0.8 , with median correlations of 0.819 and 0.768 for controls and SZ patients, respectively (Fig. 5a). For the ABIDE I training set, 224 out of 225 matrices (99.6% ) had correlations greater than 0.6 and 54 out of 225 matrices (24.0% ) had correlations greater than 0.8 , with median correlations of 0.784 and 0.762 for controls and ASD patients, respectively (Fig. 5b). High correlations were also observed for the test sets, with median correlations of 0.792 and 0.747 for the FBIRN control and patient groups (Fig. 5c), and 0.740 and 0.711 for the ABIDE I control and patient groups, respectively (Fig. 5d). The mean squared errors (MSE) between generated and original sFNC matrices were also low, with median MSEs of 0.031 , 0.034 , 0.029, and 0.034 for the FBIRN training set, FBIRN test set, ABIDE I training set, and ABIDE I test set, respectively (Supplementary Material, Section 10, Figure S13). These comparisons indicate that the generated sFNC matrices closely matched the original ones, especially for the control groups.

**Fig. 6.**
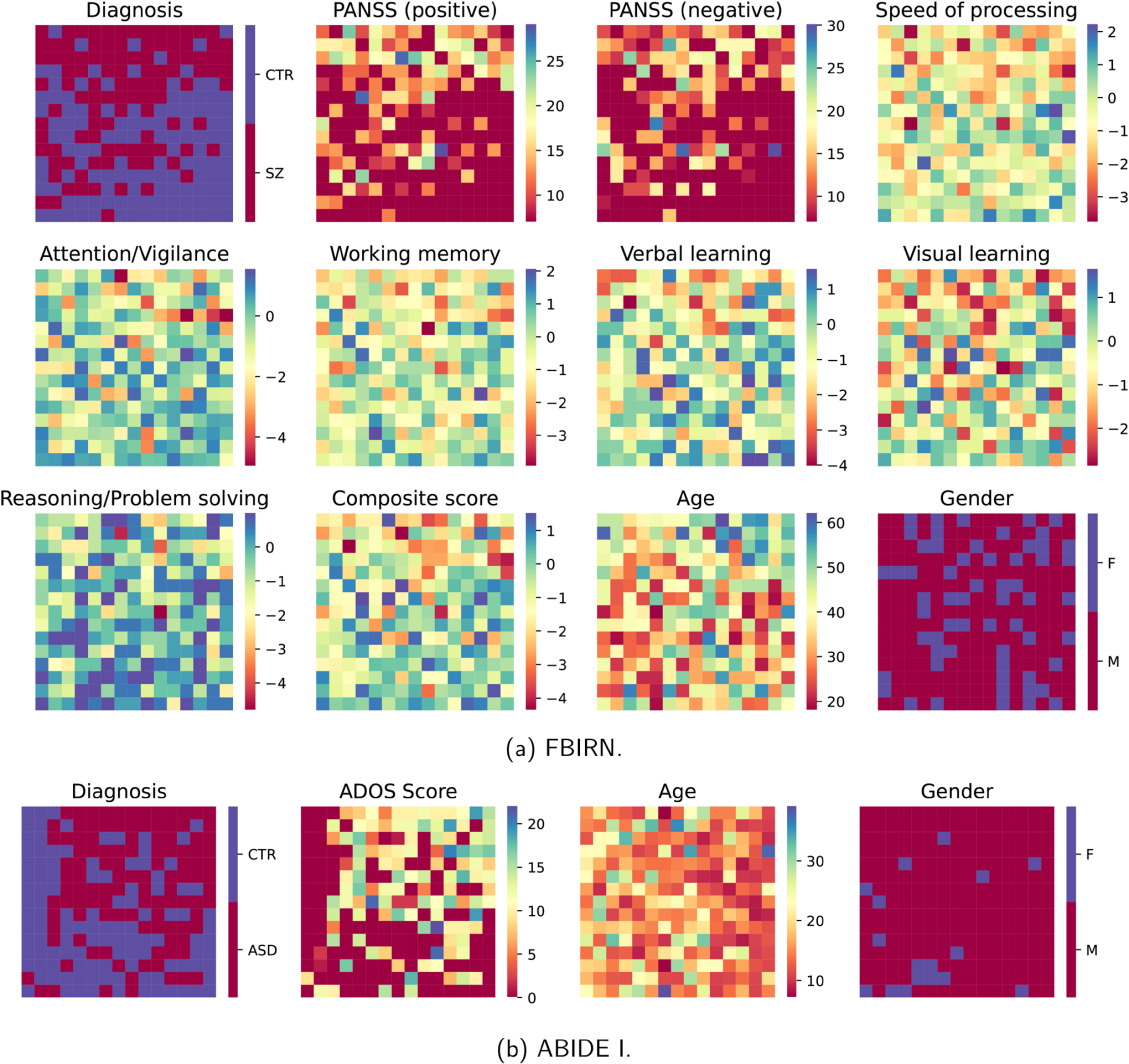
Subject measures displayed on the 2D grid. (a) Based on the mapping between latent features and 2D grid nodes (Fig. 3), we displayed the following information for FBIRN subjects on the 2D grid: diagnostic labels, PANSS positive and negative subscale scores, CMINDS cognitive scores (speed of processing, attention/vigilance, working memory, verbal learning, visual learning, reasoning/problem solving, and composite scores), age, and gender. (b) Based on the same mapping approach (Fig. 4), we displayed the following information for ABIDE I subjects on the 2D grid: diagnostic labels, ADOS scores, age, and gender.

Furthermore, we displayed multiple subject measures, including diagnostic labels, clinical assessment scores, age, gender, and FBIRN cognitive scores (van Erp et al., 2015), on the 2D grid (Fig. 6), providing interpretable visualization of how each subject measure relates to the learned latent representation. Crucially, diagnosis was a key factor distinguishing subjects in the latent space. In FBIRN, most SZ patients occupied the upper half of the 2D grid and most controls occupied the lower half (Fig. 6a). In ABIDE I, most ASD patients clustered in the upper triangle and most controls clustered in the lower triangle (Fig. 6b). In addition, age appeared to be a contributing factor in FBIRN, as younger subjects were mainly located in the lower triangle, while older subjects were mostly in the upper triangle (Fig. 6a). Interestingly, SZ patients located in the lower, left, or lower triangular half of the 2D grid consistently showed relatively higher (better) cognitive scores than those located in the upper, right, or upper triangular half, respectively (Table 2). In particular, speed of processing, reasoning/problem solving, and composite scores in the lower half were significantly higher than those in the upper half, and composite scores in the lower triangle were significantly higher than those in the upper triangle (p<0.05 , Mann–Whitney U test), suggesting that our framework can group SZ patients in the latent space based on cognitive performance. To further explore the utility of the VAE latent space for subgroup identification, we applied k-means clustering to the latent features to identify SZ subgroups, examined their associations with subject measures, and characterized subgroup-specific connectivity patterns (Supplementary Material, Section 11, Figures S14-S17, Table S6). To examine how the dimensionality of the VAE latent space affects subgroup identification, we compared the relationships between k-means subgroups and cognitive scores using both 2D and 3-dimensional (3D) latent representations and found that the 3D latent representations did not yield additional meaningful subgroups (Supplementary Material, Section 11, Figures S18-S20, Table S7). Moreover, we investigated sFNC patterns in relation to cognitive performance and identified common and distinct connectivity changes across cognitive measures (Supplementary Material, Section 12, Figures S21-S23).

**Table 2.** Cognitive measure statistics of the FBIRN patient cohort.

Cognitive measure	Upper	Lower	Left	Right	Upper triangle	Lower triangle
Diagnosis	69	36	57	48	67	38
Speed of processing	−1.35(1.0)	−0.92(0.8)*	−1.13(0.9)	−1.29(1.0)	−1.34(1.0)	−0.96(0.8)
Attention/vigilance	−1.41(1.4)	−1.05(1.2)	−1.06(1.3)	−1.55(1.4)	−1.43(1.4)	−1.05(1.2)
Working memory	−1.10(1.0)	−0.79(0.9)	−0.91(0.9)	−1.10(1.0)	−1.14(1.0)	−0.74(0.9)
Verbal learning	−1.38(1.2)	−1.11(0.9)	−1.22(1.0)	−1.37(1.2)	−1.40(1.2)	−1.10(1.0)
Visual learning	−1.09(1.1)	−0.81(1.1)	−0.92(1.0)	−1.08(1.2)	−1.11(1.1)	−0.78(1.0)
Reasoning	−0.87(1.1)	−0.40(0.9)*	−0.66(1.1)	−0.76(1.0)	−0.83(1.1)	−0.49(0.9)
Composite score	−1.70(1.2)	−1.21(1.0)*	−1.39(1.1)	−1.71(1.3)	−1.71(1.3)	−1.22(0.9)*

The 2D grid was partitioned into six regions: the upper seven rows (upper), the lower eight rows (lower); the left seven columns (left), the right eight columns (right); the upper triangle including the diagonal (upper triangle), and the lower triangle excluding the diagonal (lower triangle). The first row of the table shows the number of SZ patients in each region. The second to eighth rows show the mean (standard deviation) of each cognitive measure for SZ patients across different regions. The higher (better) mean cognitive score in each row is highlighted in bold. The star (*) indicates that cognitive scores in one half are significantly higher than those in the other half (p<0.05, Mann–Whitney U test). Overall, SZ patients located in the lower, left, or lower triangular half of the 2D grid consistently exhibited *higher* cognitive scores than those located in the upper, right, or upper triangular half, respectively, suggesting that the learned latent space captured cognitive performance differences among SZ patients.

### 3.2 dFNC interpolation reveals group-specific dynamic states

**Fig. 7.**
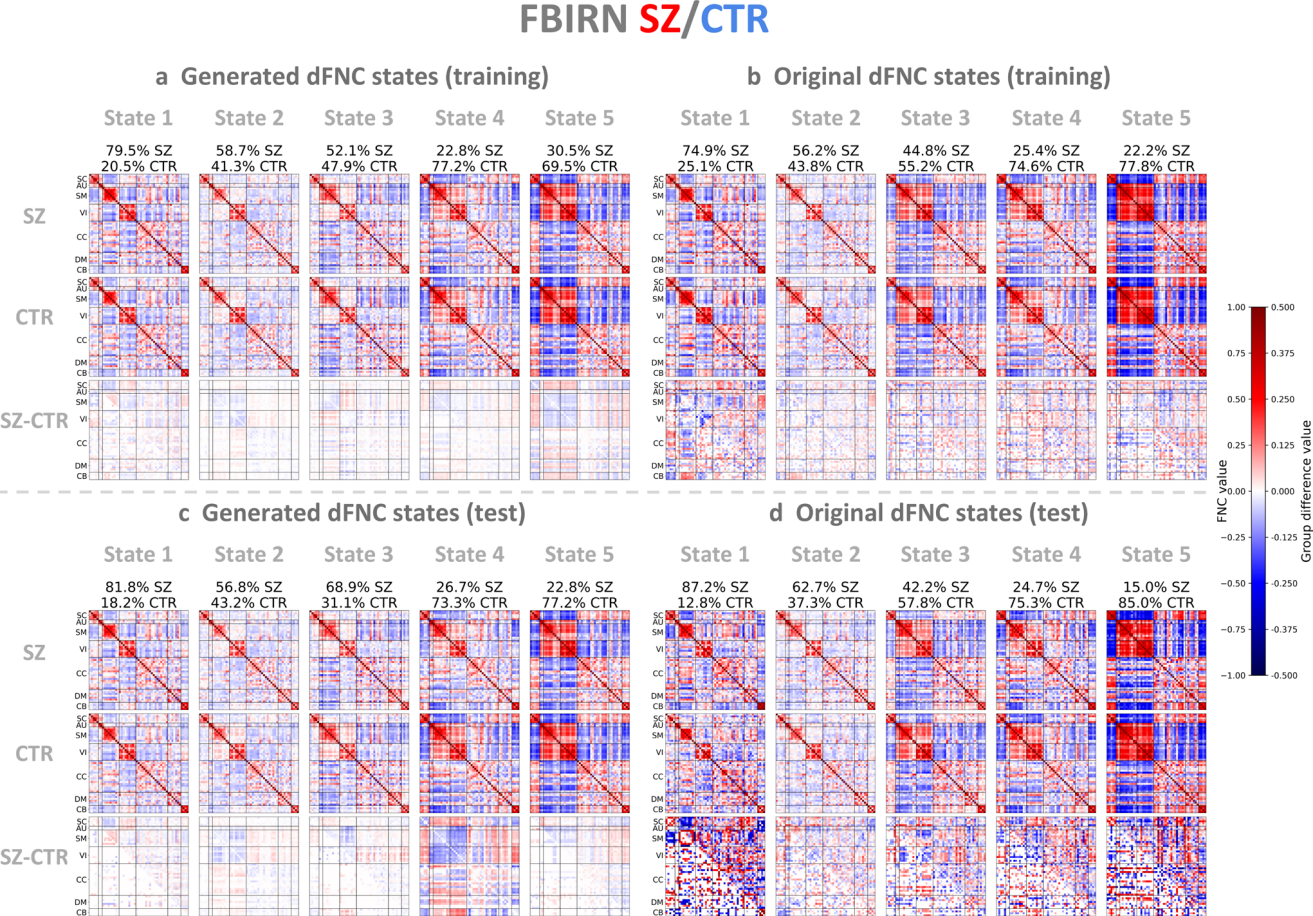
Generated and original FBIRN dFNC states. (a) Generated dFNC states of SZ patients, controls, and group differences in the training set. (b) Original dFNC states in the training set. (c) Generated dFNC states in the test set. (d) Original dFNC states in the test set. Generated and original dFNC states represent element-wise medians of dFNC patterns, obtained by applying k-means clustering to VAE latent features and dFNC matrices, respectively. Original dFNC states were sorted in increasing order of percentage of control windows relative to total windows per state, and generated states were ordered based on their similarity to the original states. Group differences between patient and control states are shown in the upper triangle, and significant differences are shown in the lower triangle (p<0.05 , two-sample t-test, Bonferroni correction); all values were multiplied by 2 for better visualization. The generated dFNC states revealed state-based group differences, similar to those observed in the original dFNC states. Moreover, the VAE effectively captured group-specific representations that generalized to the test set.

**Fig. 8.**
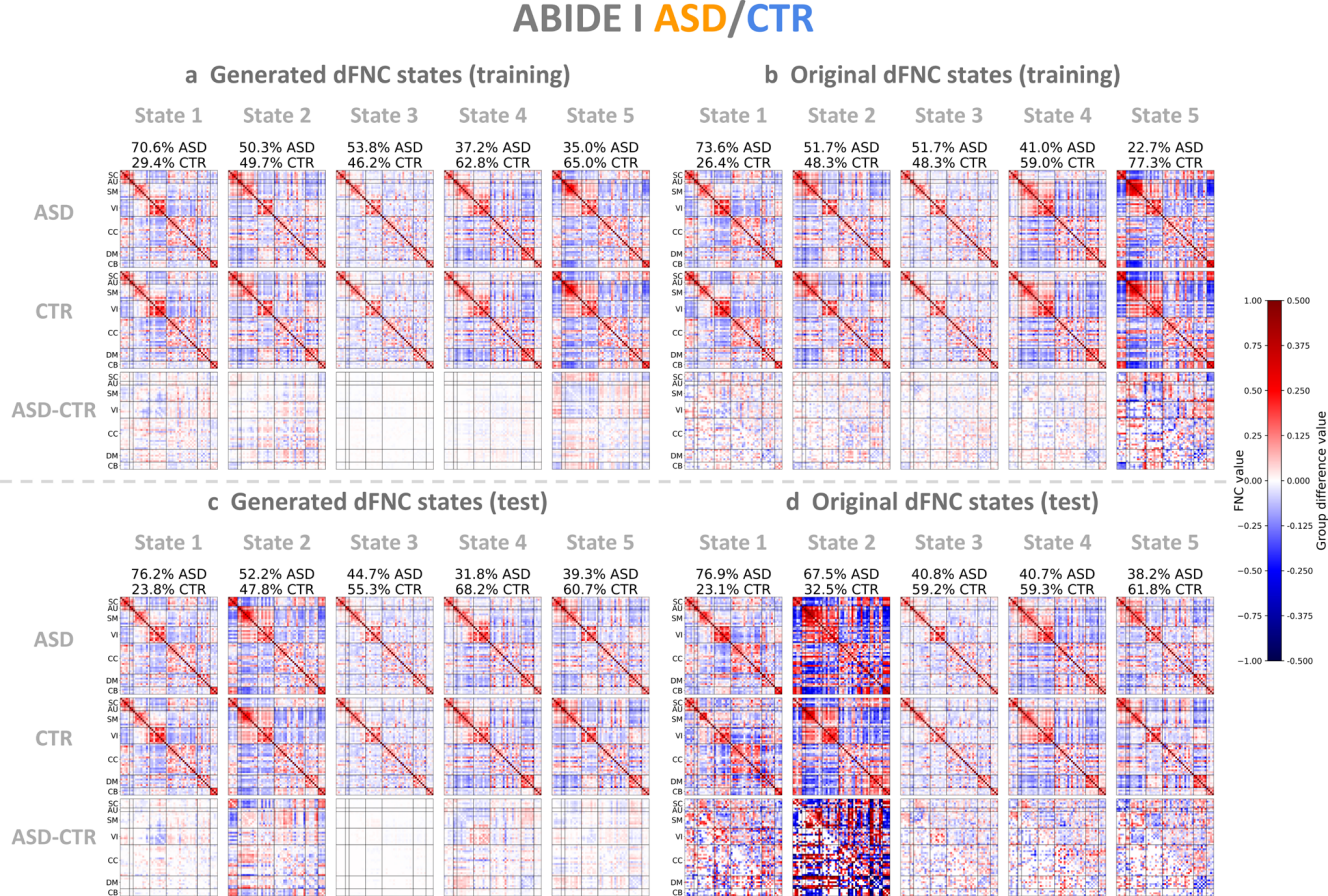
Generated and original ABIDE I dFNC states. (a) Generated dFNC states of ASD patients, controls, and group differences in the training set. (b) Original dFNC states in the training set. (c) Generated dFNC states in the test set. (d) Original dFNC states in the test set. Generated and original dFNC states represent element-wise medians of dFNC patterns, obtained by applying k-means clustering to VAE latent features and dFNC matrices, respectively. Original dFNC states were sorted in increasing order of percentage of control windows relative to total windows per state, and generated states were ordered based on their similarity to the original states. Group differences between patient and control states are shown in the upper triangle, and significant differences are shown in the lower triangle (p<0.05 , two-sample t-test, Bonferroni correction); all values were multiplied by 2 for better visualization.

To further evaluate how these group-specific patterns change over time, we conducted a dynamic state analysis by applying the k-means clustering algorithm to the dFNC data (Section 2.4.2). We searched k from 2 to 9 and considered that five clusters were optimal for both the FBIRN and ABIDE I datasets based on the elbow criterion applied to the $L_{2}$ distance (Supplementary Material, Section 13, Figure S24). We applied k-means clustering to the dFNC latent features of both patient and control groups to derive five states and then computed Pearson correlations between generated and original dFNC states separately for each diagnostic group. The group-specific dFNC states and group differences for FBIRN and ABIDE I are presented in Figures 7 and 8, respectively.

Similar to the sFNC results, the generated dFNC states were highly correlated with the original ones. Specifically, for the FBIRN training set, the correlations between generated and original dFNC states 1 to 5 were 0.990, 0.990, 0.882, 0.983, 0.967 for the SZ patient group and 0.958, 0.982, 0.872, 0.994, 0.974 for the control group (Fig. 7a, b). For the FBIRN test set, the correlations for states 1 to 5 were 0.902, 0.968, 0.891, 0.935, 0.908 for the patient group and 0.820, 0.955, 0.882, 0.967, 0.955 for the control group (Fig. 7c, d). In state 1, SZ patients exhibited stronger negative correlations between the subcortical (SC) and sensorimotor (SM) domains and between the SM and visual (VI) domains. Conversely, they showed increased positive correlations between the SC and VI domains and between the SM and cerebellar (CB) domains. In state 5, SZ patients showed stronger negative correlations between the SM and VI domains and between the SC and CB domains. They also showed stronger positive correlations between the SC and SM domains, between the SC and VI domains, and between the CB and sensory (SM, VI) domains. When evaluated on the test set, the original group difference patterns appeared noisy due to the small sample size (Fig. 7d). Nonetheless, the generated group difference patterns (Fig. 7c) closely resembled those from the training set, particularly for test state 2 vs. training state 3 (Pearson correlation coefficient r=0.811 ) and test state 4 vs. training state 4 (r=0.793 ), suggesting that the VAE effectively captured *generalizable* group-specific representations.

For the ABIDE I training set, the correlations between generated and original dFNC states 1 to 5 were 0.985, 0.994, 0.995, 0.982, 0.924 for the ASD patient group and 0.971, 0.990, 0.993, 0.984, 0.947 for the control group (Fig. 8a, b). For the ABIDE I test set, the correlations for states 1 to 5 were 0.957, 0.820, 0.974, 0.947, 0.961 for the patient group and 0.885, 0.867, 0.980, 0.969, 0.960 for the control group (Fig. 8c, d). In state 1, which was mainly occupied by ASD patients, we observed significant group differences, including negative correlations between the SM and VI domains and between the SC and SM domains, as well as positive correlations between the VI domain and the SC, CB, and DM domains. These patterns partially overlapped with the group difference patterns observed in FBIRN state 1, indicating that ASD and SZ may share similar neurobiological deficits.

**Fig. 9.**
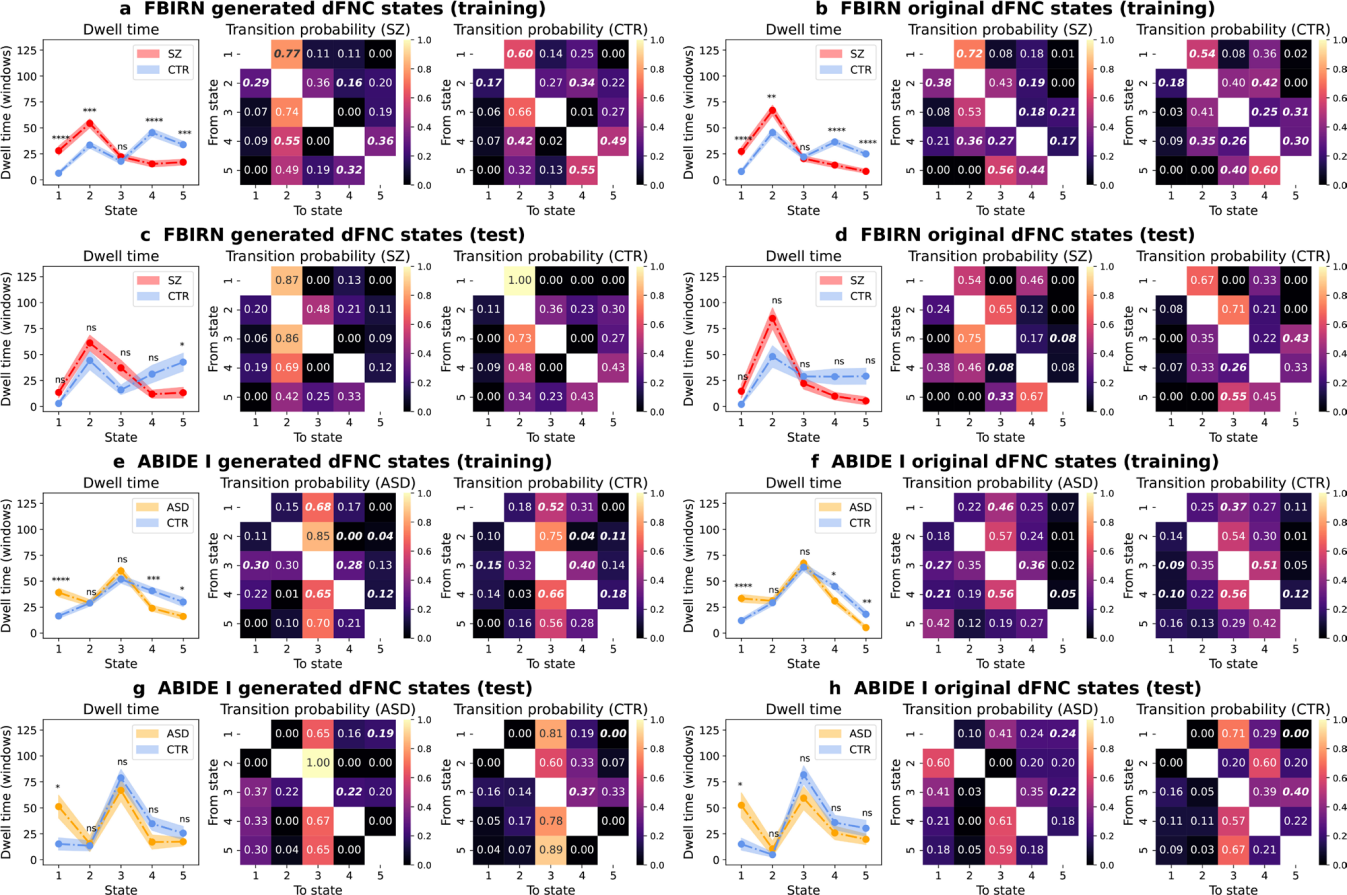
Group-specific dwell time and transition probability matrices of generated and original dFNC states. Panels (a, b, c, d) show results from FBIRN training and test sets, while panels (e, f, g, h) show results from ABIDE I training and test sets. Panels (a, c, e, g) show metrics derived from generated data and panels (b, d, f, h) present results from original data. State labels are consistent with Figures 7 and 8. In each panel, the line plot shows the mean ± standard error of dwell time for different groups (red: SZ patients; orange: ASD patients; blue: controls). Two-sample t-tests with false discovery rate (FDR) correction for five states were used to assess group differences. The number of asterisks (1, 2, 3, 4) indicates the level of significance (p<0.05 , p<0.01 , p<0.001 , p<0.0001 , FDR correction). The abbreviation “ns” stands for “not significant”. The heatmaps show transition probabilities between each pair of states for patients and controls, respectively. Transition probabilities were normalized so that each row sums to 1. Significant transition count differences between patients and controls are highlighted in bold italics (p<0.05 , two-sample t-test). The generated states closely matched the original states in group-specific properties such as dwell time and transition probabilities, indicating that VAEs effectively captured state-based dynamic properties.

Subsequently, we evaluated two group-specific state-based dynamic measures—dwell time and transition probability matrix—to further quantitatively compare the generated and original dFNC states (Fig. 9). The group-specific dwell time measures the average amount of time that a group spends in a state. The dwell time statistics of the generated states were largely consistent with those of the original states in both datasets. For the FBIRN training set (Fig. 9a, b), states 1, 2, 4, and 5 showed significant group differences in both the generated and original states (p<0.01 , two-sample t-test, FDR correction). In particular, SZ patients stayed in weakly connected states 1 and 2 significantly longer, while controls stayed in densely connected states 4 and 5 significantly longer. For the FBIRN test set (Fig. 9c, d), the generated results showed significant group differences in state 5 (p<0.05 , two-sample t-test, FDR correction), whereas the original results did not show any significant differences across all five states.

For the ABIDE I training set (Fig. 9e, f), ASD patients spent significantly longer in state 1, while controls spent significantly longer in states 4 and 5, in both the generated and original results (p<0.05 , two-sample t-test, FDR correction). No significant group differences were observed in states 2 and 3. For the ABIDE I test set (Fig. 9g, h), the dwell time statistics in the generated results were also consistent with the original ones. Only the patient-dominant state 1 showed significant group differences (p<0.05 , two-sample t-test, FDR correction).

The group-specific transition probability matrix shows the probability that a group changes from one state to another. For the FBIRN training set (Fig. 9a, b), SZ patients were more likely to transition between weakly connected states 1 and 2 (state 1 to state 2: $P_{\text{generated}}=0.77$ , $P_{\text{original}}=0.72$ ; state 2 to state 1: $P_{\text{generated}}=0.29$ , $P_{\text{original}}=0.38$ ). In contrast, controls showed significantly lower transition probabilities when moving between state 1 and state 2 (state 1 to state 2: $P_{\text{generated}}=0.60$ , $P_{\text{original}}=0.54$ ; state 2 to state 1: $P_{\text{generated}}=0.17$ , $P_{\text{original}}=0.18$ ). By comparison, controls were more likely to switch between densely connected states 4 and 5 (state 4 to state 5: $P_{\text{generated}}=0.49$ , $P_{\text{original}}=0.30$ ; state 5 to state 4: $P_{\text{generated}}=0.55$ , $P_{\text{original}}=0.60$ ), whereas patients with SZ had relatively lower probabilities of switching between states 4 and 5 (state 4 to state 5: $P_{\text{generated}}=0.36$ , $P_{\text{original}}=0.17$ ; state 5 to state 4: $P_{\text{generated}}=0.32$ , $P_{\text{original}}=0.44$ ).

Interestingly, for the ABIDE I training and test data (Fig. 9e–h), both the ASD patient and control groups tended to switch from other states to state 3, suggesting that state 3 may act as a hub for dynamic state transitions. In general, controls showed higher transition probabilities from other states to state 4, while ASD patients were more likely to transition to state 1.

Taken together, the generated states showed highly similar group-specific properties to the original ones, such as dwell time and transition probabilities, indicating that VAEs effectively learned state-based dynamic properties without explicitly incorporating temporal information during training.

### 3.3 A low-dimensional manifold for interpolating and interpreting FNC

**Fig. 10.**
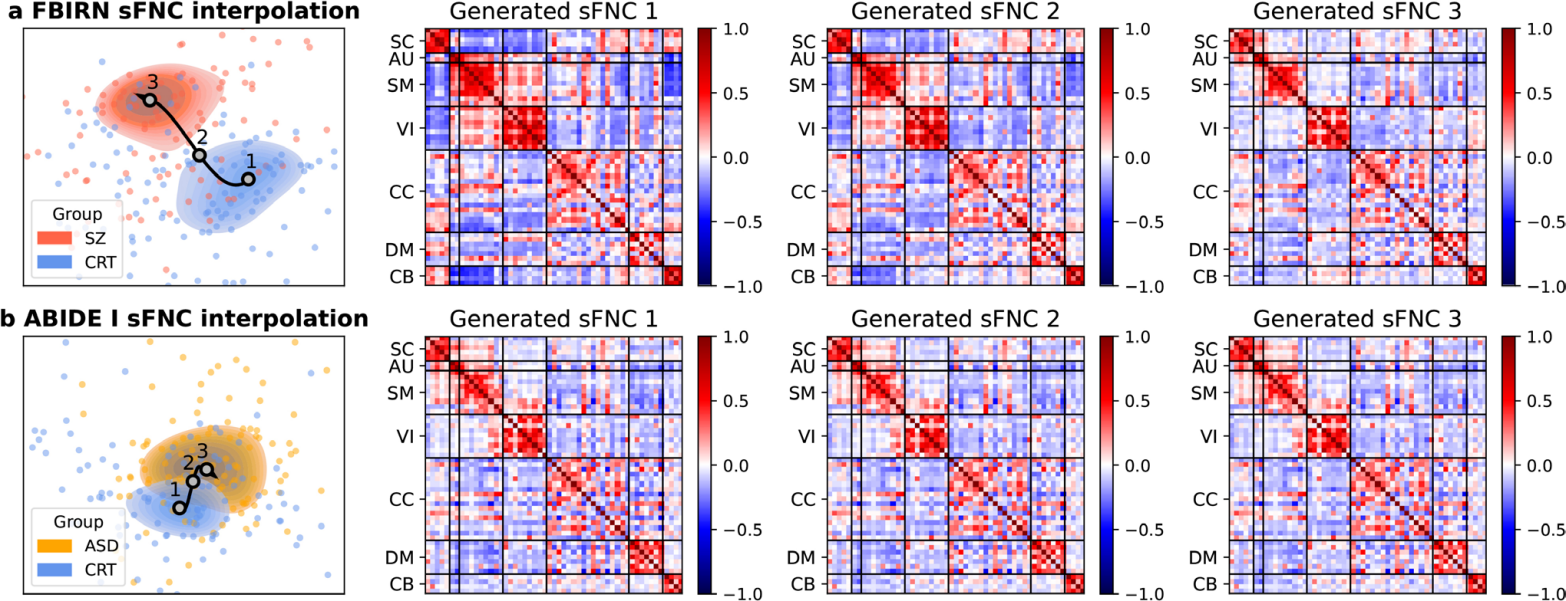
sFNC interpolation between diagnostic groups in the VAE latent space. (a) FBIRN training data. (b) ABIDE I training data. The scatter plot on the left shows latent features colored by diagnostic labels (red: SZ patients; orange: ASD patients; blue: controls), overlaid with contour plots of two clusters connected by an interpolation trajectory. The matrices on the right show three representative sFNC patterns generated by sampling from the start, midpoint, and end of the trajectory.

**Fig. 11.**
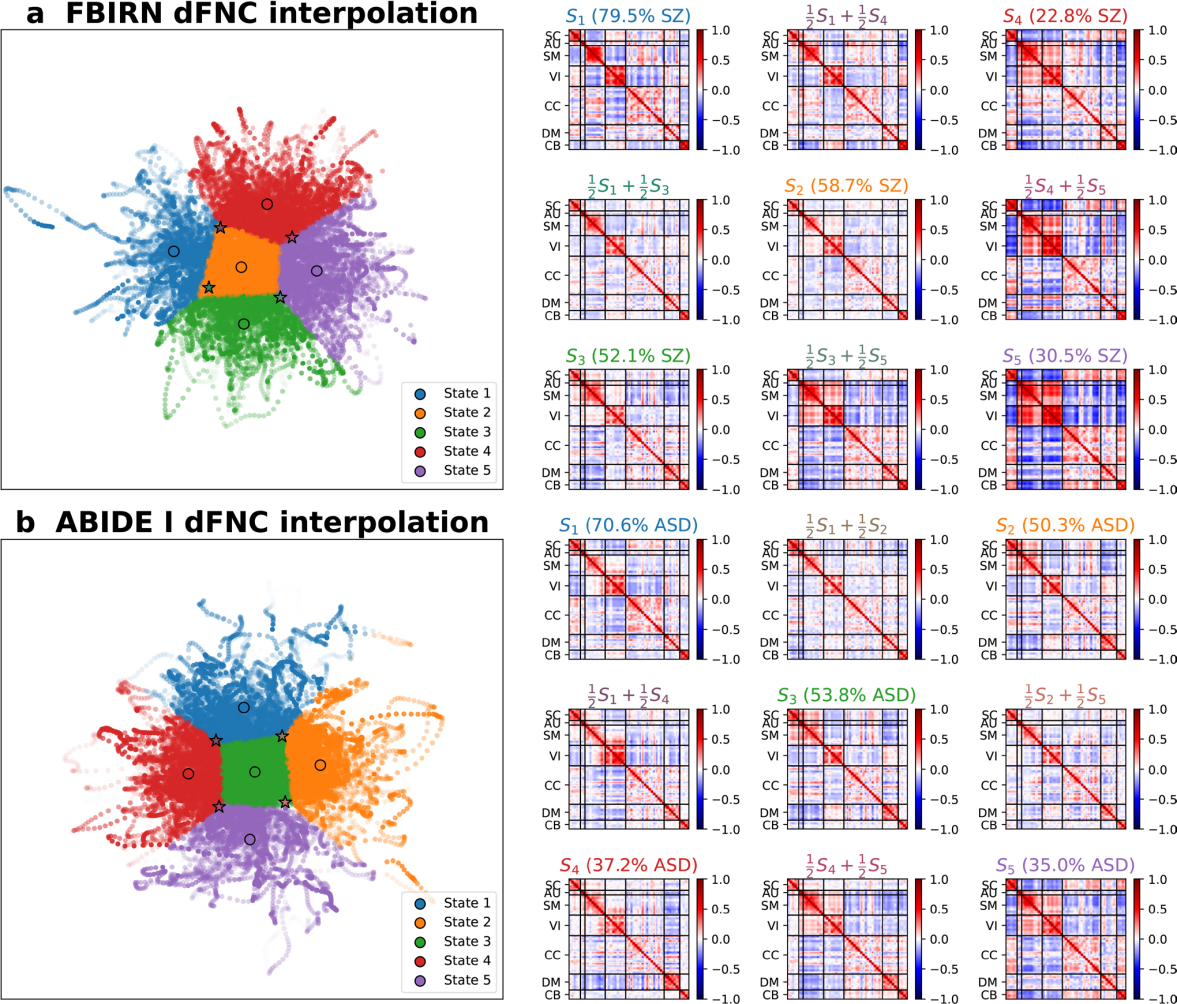
dFNC interpolation between two dynamic states in the VAE latent space. (a) FBIRN training data. (b) ABIDE I training data. The scatter plot on the left shows dFNC latent features color coded by k-means cluster assignment. Circles (∘) represent state centroids. Stars (⋆) represent midpoints between centroid pairs. The matrices on the right show samples generated from the five state centroids and the four midpoints between centroid pairs, illustrating transition patterns between dynamic states.

In the interpolation framework, the VAE latent space serves as a low-dimensional manifold for interpolating and interpreting sFNC and dFNC matrices, providing insights into how FNC patterns change along a trajectory of interest. Here, we show two potentially useful applications of the interpolation framework: (1) sFNC interpolation between patient and control groups to estimate a psychosis continuum (Fig. 10) and (2) dFNC interpolation between two states to investigate state transition patterns (Fig. 11).

To visualize how sFNC continuously changes between the control group and the patient group, we interpolated along a trajectory from the control center to the patient center (Fig. 10). When interpolating along the trajectory from the control center to the SZ patient center, we observed significant connectivity changes across multiple domains. Specifically, anti-correlations became significantly weaker in the SC–SM, SC–VI, and SM–CB domains, whereas positive correlations became significantly weaker within the SM, VI, and CC domains. In addition, positive correlations changed significantly to negative correlations in the SC–CB and SM–VI domains, while negative correlations changed significantly to positive correlations in the VI–CB domain (p<0.0001 , Wilcoxon signed-rank test, Bonferroni correction; Fig. 10a). When interpolating along the trajectory from the control center to the ASD patient center, we observed partially overlapping but distinct connectivity patterns. Similar to SZ, negative correlations were significantly stronger in the SC–CB domain but weaker in the VI–CB domain. In contrast, ASD showed unique alterations, including significantly weaker negative correlations in the SM–DM and VI–DM domains, stronger negative correlations in the SC–VI and VI–CC domains, decreased positive correlations within the DM domain, and increased positive correlations within the CC domain (p<0.0001 , Wilcoxon signed-rank test, Bonferroni correction; Fig. 10b).

To evaluate how one dynamic state gradually transitions to another, we generated an FNC matrix by sampling from the distribution at the midpoint between two state centroids in the latent space (Fig. 11). When an SZ-dominant state (e.g., state 1) transitioned to a control-dominant state (e.g., state 5), we observed significantly stronger negative correlations in the SC–SM, SM–CB, and VI–CC domains and significantly stronger positive correlations between the SC and CC domains and within the VI domain. Additionally, negative correlations shifted significantly to positive in the SC–CB, SM–VI, and CC–CB domains, while positive correlations shifted significantly to negative in the SC–VI, AU–CC, SM–CC, and VI–CB domains (p<0.0001 , Wilcoxon signed-rank test, Bonferroni correction; Fig. 11a). Similarly, when an ASD-dominant state (e.g., state 1) transitioned to a control-dominant state (e.g., state 5), we noticed significantly stronger negative correlations in the SC–SM, SC–VI, SM–CC, and SM–CB domains and significantly stronger positive correlations between the SM and VI domains and within the SM domain. Furthermore, negative correlations shifted significantly to positive in the SC–CB and CC–CB domains, while positive correlations shifted significantly to negative in the VI–CB domain, consistent with the SZ findings (p<0.0001 , Wilcoxon signed-rank test, Bonferroni correction; Fig. 11b).

## 4 Discussion

This work presents a VAE-based FNC interpolation framework to investigate the neuropsychiatric continuum and heterogeneity from static and dynamic FNC data. Our framework provides additional advantages that complement existing diagnostic and analytical methods. To identify neurobiological markers of SZ and ASD, we leveraged FNC derived from fMRI—an objective measure of brain functional differences in psychiatric and neurodevelopmental populations. Unlike supervised approaches, our *unsupervised generative* model learns hidden relationships in a fully data-driven manner and generates continuous FNC patterns along the latent disorder spectrum. The high correlations between generated and original sFNC matrices demonstrate that the VAE captured representative and generalizable latent distributions in both datasets, outperforming a linear baseline (PPCA) and a semi-supervised alternative (iVAE) (Figs. 2 and 5). Notably, the unsupervised VAE slightly outperformed the semi-supervised iVAE, likely because conditioning the iVAE’s prior on auxiliary variables, rather than a simple isotropic Gaussian, makes optimization harder and limits reconstruction performance. In addition, we chose the diagnostic label as the auxiliary variable, yet the most appropriate auxiliary information for neuroimaging data remains unknown. These findings support the VAE as the most suitable model for our interpolation task, given their ability to learn nonlinear latent relationships in an unsupervised manner.

Unlike previous studies focusing on group-level comparisons, our framework provides a generative approach to model a functional connectivity continuum between groups. Specifically, the interpolated continua from controls to patients showed two common FNC gradients in both SZ and ASD: (1) reduced positive correlations within the sensory networks (auditory, sensorimotor, visual) and between the subcortical and cerebellar domains; (2) reduced negative correlations between the subcortical domain and the sensory domains, as well as between the cerebellar domain and these sensory domains (Figs. 3 and 4). Consistent with these findings, previous studies using NeuroMark sFNC have reported overlapping group-level alterations in SZ and ASD, particularly between the subcortical domain and the cerebellar, auditory, and sensorimotor domains (Du et al., 2020; Jensen et al., 2024; Yan et al., 2024). We also identified disorder-specific patterns. For example, SZ showed reduced connectivity in the visual domain, whereas ASD showed decreased connectivity within the default mode network, which aligns with previous work (Du et al., 2020).

Another key benefit of our framework is the visualization and characterization of individual differences. Rather than relying solely on group-average patterns, we designed a 2D grid to organize sFNC matrices, supporting examination of subject-level variability and inter-subject relationships (Figs. 3 and 4). In addition to sFNC matrices, we projected multiple subject measures, including cognitive scores, onto the same 2D grid (Fig. 6). Notably, SZ patients in the lower, left, or lower triangular half of the 2D grid consistently showed higher cognitive scores, indicating better cognitive function (Table 2).

Moreover, we identified SZ subgroups by applying k-means clustering to the learned latent features and then examined subgroup differences in subject measures and FNC patterns (Supplementary Material, Section 11). Changes related to working memory were observed across multiple network pairs, including sensorimotor–default mode, sensorimotor–cerebellar, and cognitive control–cerebellar networks. Previous studies have similarly linked working memory deficits in SZ to weaker connectivity between the fronto-parietal network and cerebellar network (Repovs et al., 2011), as well as stronger within-network connectivity in the fronto-parietal and default mode networks (Unschuld et al., 2013). The default mode network was also associated with speed of processing, similar to previous reports (Mwansisya et al., 2013; Wang et al., 2014). In addition, attention performance was related to connectivity changes between the default mode and cognitive control networks and each of the sensorimotor, cerebellar, and auditory domains, in line with prior work proposing the insula as a key hub modulating activity between the default mode network and the task-positive networks (Fox et al., 2005; Z. He et al., 2013; Moran et al., 2013).

We further examined how sFNC patterns varied in relation to cognitive performance by interpolating along a trajectory from the higher-score group to the lower-score group (Supplementary Material, Section 12, Figure S22). Compared with the higher-score group, the lower-score group exhibited significantly attenuated negative correlations in the subcortical–sensorimotor, subcortical–visual, and sensorimotor–cerebellar domains, as well as reduced positive correlations within the sensorimotor, visual, and cognitive control domains, consistent with prior evidence linking cognitive deficits in SZ to reduced cortical–subcortical functional connectivity (Sheffield & Barch, 2016).

Beyond static connectivity, we evaluated whether the VAE can capture dynamic properties from windowed dFNC data. The generated dFNC states closely matched the original states (Figs. 7 and 8) and exhibited consistent group-specific properties, measured by dwell time and transition probability (Fig. 9). In particular, both generated and original states showed that SZ and ASD patients spent longer in sparsely connected states, while controls spent longer in densely connected states, in agreement with the literature (Damaraju et al., 2014; Fu et al., 2019, 2021).

A VAE is designed to learn latent distributions and capture hidden relationships from observed data in an unsupervised manner. By leveraging the VAE latent space, we interpolated along a trajectory from the control center to the patient center to visualize continuous sFNC alterations (Fig. 10). Similarly, we generated samples between two dFNC state centroids to examine state transition patterns (Fig. 11). Together, we demonstrate the ability of the VAE to capture representative group-specific FNC profiles and generate continuous FNC patterns across different subjects, groups, and dynamic states.

Despite its promising potential, our current framework has several limitations. First, we used a vanilla VAE with a multivariate Gaussian prior assumption, which might not fully capture the heterogeneity or hierarchical structures in SZ and ASD. Second, in order to generate continuous sFNC data, we sampled from evenly spaced coordinates in the latent space, rather than the posterior distributions, so the generated sFNC matrices and the original sFNC matrices ordered by the JV algorithm were not one-to-one matched. Lastly, we assumed that FNC differences between groups are continuous and can be interpolated. While whether psychosis represents a continuum or discrete categories remains debated (David, 2010; DeRosse & Karlsgodt, 2015; Lawrie et al., 2010), growing evidence suggests the shift from the concept of schizophrenia to a broader psychotic spectrum capturing a continuum of psychosis proneness from normal to abnormal (Guloksuz & van Os, 2018; van Os et al., 2009).

In the future, we aim to incorporate hierarchical clustering or mixture model priors to better characterize psychotic syndromes, and to automatically capture dynamic states using dynamical VAEs (Girin et al., 2022). To validate the continuity assumption and improve generalizability, we will apply the framework to large-scale longitudinal datasets with multiple mental disorders, such as the Adolescent Brain and Cognitive Development (ABCD) dataset (Barch et al., 2018; Casey et al., 2018; Garavan et al., 2018).

## 5 Conclusions

We presented a VAE-based FNC interpolation framework that captures individual variability within groups, continuous FNC alterations between groups, and group-specific dFNC dynamic states. The generated sFNC patterns and dFNC states showed high correspondence with the original data, and interpolation along trajectories of interest in the latent space revealed continuous FNC alterations in the original space. Our framework offers novel insights into the neuropsychiatric continuum and heterogeneity, marking a promising step toward personalized characterization of mental disorders.

## Supplementary Material

Supplementary Material

## Data Availability

The FBIRN dataset can be accessed at https://www.nitrc.org/projects/fbirn/. The ABIDE I dataset can be accessed at https://fcon_1000.projects.nitrc.org/indi/abide/. The NeuroMark network templates are available at http://trendscenter.org/software. The analysis and visualization code is publicly available at https://github.com/XinhuiLi/interpolation.git.

## Footnotes

1 The multi-site ABIDE I data with different TRs were resampled based on the minimum TR across all sites (TR=1.5s). The window width was selected to maintain a consistent temporal duration of approximately 40s across both datasets.

2 A statistical model is identifiable if the mapping from each set of its parameters to the distribution of observed data is injective (Lehmann & Casella, 1998).

3 A preliminary version of this work was published at EMBC 2022 (Li et al., 2022). The current study extends that work by (1) incorporating the ABIDE I dataset, (2) comparing the VAE with PPCA and iVAE, (3) identifying SZ subgroups in the FBIRN dataset, (4) performing dFNC interpolation in both datasets, (5) providing interpolation examples, and (6) adding further interpretation of FNC findings with respect to diagnostic and cognitive scores.
