# Supplementary material for: Brain functional network connectivity interpolation characterizes the neuropsychiatric continuum and heterogeneity
Source: Imaging Neurosci (Camb). 2026 Apr 27;4:IMAG.a.1220. doi: 10.1162/IMAG.a.1220 (PMC13125074; doi:10.1162/IMAG.a.1220)
Supplement: Supplementary Material [file IMAG.a.1220_supp.pdf]

Supplementary Material

1 ABIDE I data information

In the ABIDE I dataset, 266 subjects were used from the following three sites: (1) NYU Langone Medical Center (NYU), (2) University of California, Los Angeles: Sample 1 (UCLA) and (3) University of Utah School of Medicine (USM). Site-specific demographic information is shown in Table S1. fMRI acquisition parameters for each site are shown in Table S2.

Table S1: **ABIDE I site-specific demographic information.** Abbreviations: N, number of total subjects; N_{male}, number of male subjects; N_{female}, number of female subjects; s.d., standard deviation.

Site	N	N_{male}	N_{female}	Age mean ± s.d. (years)	Age range (years)
			ASD		
NYU	64	55	9	15.72 ± 7.11	$7.15 - 39.10$
UCLA	41	36	5	13.54 ± 2.25	$9.26 - 17.94$
USM	28	28	0	25.05 ± 6.11	$15.93 - 37.78$
			CTR		
NYU	95	70	25	16.45 ± 6.12	$7.29 - 31.78$
UCLA	29	25	4	13.35 ± 1.96	$9.50 - 17.79$
USM	9	9	0	20.57 ± 4.65	$13.81 - 27.60$

Table S2: **ABIDE I site-specific fMRI acquisition parameters.**

Site	Scanner	TR (ms)	TE (ms)	FA (degree)	Voxel size (mm)	Slices
NYU	Siemens Magnetom Allegra syngo MR 2004A	2000	15	90	$3.0 \times 3.0 \times 4.0$	33
UCLA	Siemens Magnetom TrioTim syngo MR B15	3000	28	90	$3.0 \times 3.0 \times 4.0$	34
USM	Siemens Magnetom TrioTim syngo MR B17	2000	28	90	$3.4 \times 3.4 \times 3.0$	40

2 Statistics of subject measures

Subject measures included the Positive and Negative Syndrome Scale (PANSS) scores (Kay et al., 1987), six cognitive domain scores (speed of processing, attention/vigilance, working memory, verbal learning, visual learning, and reasoning/problem solving) and composite scores derived from the Computerized Multiphasic Interactive Neurocognitive System (CMINDS) (van Erp et al., 2015) for FBIRN, and the Autism Diagnostic Observation Schedule (ADOS) scores (Lord et al., 2000) for ABIDE I. Table S3 summarizes the group-level mean, standard deviation, and range for each subject measure in each dataset.

Table S3: **Statistics of subject measures.** Abbreviations: PT, patient; CTR, control.

Subject measure	PT mean ± s.d.	PT range	CTR mean ± s.d.	CTR range
		FBIRN		
PANSS positive score	14.976 ± 4.75	$7 - 29$	7.000 ± 0.00	$7 - 7$
PANSS negative score	13.928 ± 5.00	$7 - 30$	7.000 ± 0.00	$7 - 7$
Speed of processing	-1.217 ± 0.98	$-3.682 - 1.165$	0.038 ± 1.00	$-3.764 - 2.195$
Attention/Vigilance	-1.277 ± 1.36	$-4.965 - 1.390$	0.026 ± 0.98	$-4.965 - 1.542$
Working memory	-1.018 ± 0.97	$-3.947 - 2.053$	0.020 ± 0.96	$-3.019 - 2.053$
Verbal learning	-1.313 ± 1.17	$-4.034 - 1.561$	0.042 ± 0.99	$-3.101 - 1.561$
Visual learning	-1.002 ± 1.13	$-2.873 - 1.152$	0.026 ± 0.99	$-2.873 - 1.646$
Reasoning/Problem solving	-0.756 ± 1.11	$-4.443 - 0.981$	0.022 ± 0.97	$-4.805 - 0.981$
Composite score	-1.562 ± 1.22	$-4.502 - 1.496$	0.044 ± 0.95	$-4.023 - 1.550$
		ABIDE I		
ADOS score	11.594 ± 4.00	$2 - 22$	0.090 ± 0.47	$0 - 4$

3 Variational autoencoders

Here, we provide a step-by-step derivation of the VAE objective function. We refer readers to Kingma and Welling, 2014 for the original theory and to Tiao, 2017 for a detailed tutorial on VAEs.

Let $\mathbf{x} \in \mathbb{R}^V$ be an observed data variable and $\mathbf{z} \in \mathbb{R}^D$ be its corresponding latent variable. We assume that the latent variable $\mathbf{z}$ is sampled from a prior distribution $p(\mathbf{z})$ and the observed variable $\mathbf{x}$ is sampled from the conditional likelihood distribution $p_{\boldsymbol{\theta}}(\mathbf{x}|\mathbf{z})$ parameterized by $\boldsymbol{\theta}$. We consider a latent variable model with the following joint distribution:

$$p_{\boldsymbol{\theta}}(\mathbf{x}, \mathbf{z}) = p_{\boldsymbol{\theta}}(\mathbf{x}|\mathbf{z})p(\mathbf{z}). \tag{S1}$$

We would like to estimate the posterior distribution in order to perform inference on the latent variable. According to Bayes' theorem, the posterior can be computed as:

$$p_{\boldsymbol{\theta}}(\mathbf{z}|\mathbf{x}) = \frac{p_{\boldsymbol{\theta}}(\mathbf{x}, \mathbf{z})}{p_{\boldsymbol{\theta}}(\mathbf{x})} = \frac{p_{\boldsymbol{\theta}}(\mathbf{x}|\mathbf{z})p(\mathbf{z})}{p_{\boldsymbol{\theta}}(\mathbf{x})} = \frac{p_{\boldsymbol{\theta}}(\mathbf{x}|\mathbf{z})p(\mathbf{z})}{\int p_{\boldsymbol{\theta}}(\mathbf{x}|\mathbf{z})p(\mathbf{z})\,\mathrm{d}\mathbf{z}}. \tag{S2}$$

The denominator $p_{\boldsymbol{\theta}}(\mathbf{x}) = \int p_{\boldsymbol{\theta}}(\mathbf{x}|\mathbf{z})p(\mathbf{z})\,\mathrm{d}\mathbf{z}$ is the marginal distribution of the observed data, also known as the *evidence*. It is analytically intractable because there is no closed form for the integral term.

To overcome this intractability, we leverage *variational inference*, which assumes a simpler distribution to approximate the true posterior. We want to estimate the optimal variational parameters $\boldsymbol{\phi}^*$ by minimizing the Kullback-Leibler (KL) divergence[1] between the approximate posterior $q_{\boldsymbol{\phi}}(\mathbf{z}|\mathbf{x})$ and the true posterior $p_{\boldsymbol{\theta}}(\mathbf{z}|\mathbf{x})$:

$$\boldsymbol{\phi}^* = \arg\min_{\boldsymbol{\phi}} D_{\mathsf{KL}}\left(q_{\boldsymbol{\phi}}(\mathbf{z}|\mathbf{x}) \| p_{\boldsymbol{\theta}}(\mathbf{z}|\mathbf{x})\right), \tag{S3}$$

where $D_{\mathsf{KL}}\left(q_{\boldsymbol{\phi}}(\mathbf{z}|\mathbf{x}) \| p_{\boldsymbol{\theta}}(\mathbf{z}|\mathbf{x})\right) = 0$ if and only if $q_{\boldsymbol{\phi}}(\mathbf{z}|\mathbf{x}) = p_{\boldsymbol{\theta}}(\mathbf{z}|\mathbf{x})$. This is again intractable due to $p_{\boldsymbol{\theta}}(\mathbf{z}|\mathbf{x})$. However, note that the true posterior is proportional to the joint distribution, $p_{\boldsymbol{\theta}}(\mathbf{z}|\mathbf{x}) \propto p_{\boldsymbol{\theta}}(\mathbf{x}|\mathbf{z})p(\mathbf{z})$. Thus, we can minimize the following KL divergence instead:

$$\boldsymbol{\phi}^* = \arg\min_{\boldsymbol{\phi}} D_{\mathsf{KL}}\left(q_{\boldsymbol{\phi}}(\mathbf{z}|\mathbf{x}) \| p_{\boldsymbol{\theta}}(\mathbf{x}|\mathbf{z})p(\mathbf{z})\right). \tag{S4}$$

The *evidence lower bound* (ELBO) is defined to maximize the negative KL divergence in Equation S4

[1] The KL divergence measures how a probability distribution differs from another probability distribution. For a discrete random variable, $D_{\mathsf{KL}}(p(x)\|q(x)) = \sum_{x\in\mathcal{X}} p(x)\log\frac{p(x)}{q(x)}$; for a continuous random variable, $D_{\mathsf{KL}}(p(x)\|q(x)) = \int_{-\infty}^{+\infty} p(x)\log\frac{p(x)}{q(x)}\,\mathrm{d}x$.

by optimizing the generative parameters $\boldsymbol{\theta}$ and the variational parameters $\boldsymbol{\phi}$:

$$\begin{aligned}\max \mathcal{L}_{\mathsf{VAE}} &= \max_{\boldsymbol{\theta},\boldsymbol{\phi}} -D_{\mathsf{KL}}(q_{\boldsymbol{\phi}}(\mathbf{z}|\mathbf{x})\|p_{\boldsymbol{\theta}}(\mathbf{x}|\mathbf{z})p(\mathbf{z})) \\ &= \max_{\boldsymbol{\theta},\boldsymbol{\phi}} -\mathbb{E}_{q_{\boldsymbol{\phi}}(\mathbf{z}|\mathbf{x})}\left[\log q_{\boldsymbol{\phi}}(\mathbf{z}|\mathbf{x}) - \log p_{\boldsymbol{\theta}}(\mathbf{x}|\mathbf{z}) - \log p(\mathbf{z})\right] \\ &= \max_{\boldsymbol{\theta},\boldsymbol{\phi}} \mathbb{E}_{q_{\boldsymbol{\phi}}(\mathbf{z}|\mathbf{x})}\left[\log p_{\boldsymbol{\theta}}(\mathbf{x}|\mathbf{z}) + \log p(\mathbf{z}) - \log q_{\boldsymbol{\phi}}(\mathbf{z}|\mathbf{x})\right] \\ &= \max_{\boldsymbol{\theta},\boldsymbol{\phi}} \mathbb{E}_{q_{\boldsymbol{\phi}}(\mathbf{z}|\mathbf{x})}\left[\log p_{\boldsymbol{\theta}}(\mathbf{x}|\mathbf{z})\right] - D_{\mathsf{KL}}(q_{\boldsymbol{\phi}}(\mathbf{z}|\mathbf{x})\|p(\mathbf{z})).\end{aligned} \tag{S5}$$

Crucially, maximizing the ELBO with respect to the generative parameters $\boldsymbol{\theta}$ approximately maximizes the log marginal likelihood of the observed data, while maximizing it with respect to the variational parameters $\boldsymbol{\phi}$ equivalently minimizes the KL divergence. Also, note that the KL divergence is non-negative, i.e., $D_{\mathsf{KL}}(\cdot) \geq 0$. Thus, the ELBO is the lower bound to the log likelihood of generating the observed data, i.e., $\mathcal{L}_{\mathsf{ELBO}} \leq \log p_{\boldsymbol{\theta}}(\mathbf{x})$.

In this work, we assume that both the variational posterior and the prior follow multivariate Gaussian distributions:

$$q_{\boldsymbol{\phi}}(\mathbf{z}|\mathbf{x}) = \mathcal{N}\left(\mathbf{z}|\boldsymbol{\mu}_{\boldsymbol{\phi}}(\mathbf{x}), \mathsf{diag}\left(\boldsymbol{\sigma}^2_{\boldsymbol{\phi}}(\mathbf{x})\right)\right), \quad p(\mathbf{z}) = \mathcal{N}\left(\mathbf{z}|\mathbf{0},\mathbf{I}\right). \tag{S6}$$

Then the KL term in Equation S5 can be rewritten as:

$$D_{\mathsf{KL}}(q_{\boldsymbol{\phi}}(\mathbf{z}|\mathbf{x})\|p(\mathbf{z})) = \mathbb{E}_{q_{\boldsymbol{\phi}}(\mathbf{z}|\mathbf{x})}\left[\log q_{\boldsymbol{\phi}}(\mathbf{z}|\mathbf{x}) - \log p(\mathbf{z})\right] \tag{S7}$$

$$= \mathbb{E}_{q_{\boldsymbol{\phi}}(\mathbf{z}|\mathbf{x})}\left[\log\left(\frac{1}{\boldsymbol{\sigma}_{\boldsymbol{\phi}}(\mathbf{x})\sqrt{2\pi}}e^{-\frac{1}{2}\left(\frac{\mathbf{z}-\boldsymbol{\mu}_{\boldsymbol{\phi}}(\mathbf{x})}{\boldsymbol{\sigma}_{\boldsymbol{\phi}}(\mathbf{x})}\right)^2}\right) - \log\left(\frac{1}{\sqrt{2\pi}}e^{-\frac{1}{2}\mathbf{z}^2}\right)\right] \tag{S8}$$

$$= \mathbb{E}_{q_{\boldsymbol{\phi}}(\mathbf{z}|\mathbf{x})}\left[\log\left(\frac{1}{\boldsymbol{\sigma}_{\boldsymbol{\phi}}(\mathbf{x})\sqrt{2\pi}}\right) - \frac{1}{2}\left(\frac{\mathbf{z}-\boldsymbol{\mu}_{\boldsymbol{\phi}}(\mathbf{x})}{\boldsymbol{\sigma}_{\boldsymbol{\phi}}(\mathbf{x})}\right)^2 - \log\left(\frac{1}{\sqrt{2\pi}}\right) + \frac{1}{2}\mathbf{z}^2\right] \tag{S9}$$

$$= \mathbb{E}_{q_{\boldsymbol{\phi}}(\mathbf{z}|\mathbf{x})}\left[\log\left(\frac{\sqrt{2\pi}}{\boldsymbol{\sigma}_{\boldsymbol{\phi}}(\mathbf{x})\sqrt{2\pi}}\right)\right] - \frac{1}{2}\mathbb{E}_{q_{\boldsymbol{\phi}}(\mathbf{z}|\mathbf{x})}\left[\frac{(\mathbf{z}-\boldsymbol{\mu}_{\boldsymbol{\phi}}(\mathbf{x}))^2}{\boldsymbol{\sigma}^2_{\boldsymbol{\phi}}(\mathbf{x})}\right] + \frac{1}{2}\mathbb{E}_{q_{\boldsymbol{\phi}}(\mathbf{z}|\mathbf{x})}\left[\mathbf{z}^2\right] \tag{S10}$$

$$= -\log\boldsymbol{\sigma}_{\boldsymbol{\phi}}(\mathbf{x}) - \frac{1}{2} + \frac{1}{2}\left(\boldsymbol{\sigma}^2_{\boldsymbol{\phi}}(\mathbf{x}) + \boldsymbol{\mu}^2_{\boldsymbol{\phi}}(\mathbf{x})\right) \tag{S11}$$

$$= -\frac{1}{2}\cdot\left(1 + \log\boldsymbol{\sigma}^2_{\boldsymbol{\phi}}(\mathbf{x}) - \boldsymbol{\mu}^2_{\boldsymbol{\phi}}(\mathbf{x}) - \boldsymbol{\sigma}^2_{\boldsymbol{\phi}}(\mathbf{x})\right). \tag{S12}$$

To simplify Equation S10 to Equation S11, we leverage the definition of variance,

$$\boldsymbol{\sigma}^2_{\boldsymbol{\phi}}(\mathbf{x}) = \mathbb{E}\left[(\mathbf{z}-\boldsymbol{\mu}_{\boldsymbol{\phi}}(\mathbf{x}))^2\right] = \mathbb{E}\left[\mathbf{z}^2\right] - \mathbb{E}\left[\mathbf{z}\right]^2. \tag{S13}$$

Thus, $\mathbb{E}_{q_{\phi}(\mathbf{z}|\mathbf{x})}\left[(\mathbf{z}-\boldsymbol{\mu}_{\phi}(\mathbf{x}))^2\right] = \boldsymbol{\sigma}^2_{\phi}(\mathbf{x})$ and $\mathbb{E}_{q_{\phi}(\mathbf{z}|\mathbf{x})}\left[\mathbf{z}^2\right] = \boldsymbol{\sigma}^2_{\phi}(\mathbf{x}) + \boldsymbol{\mu}^2_{\phi}(\mathbf{x})$.

In order to use gradient descent, the VAE objective function is defined to minimize the reconstruction error measured by the mean squared error (MSE) and the KL divergence in Equation S12, which equivalently minimizes the negative ELBO in Equation S5:

$$\mathcal{L}_{\text{VAE}} = \min_{\boldsymbol{\theta},\boldsymbol{\phi}} \frac{1}{V}\sum_{i=1}^{V}(x_i - \hat{x}_i)^2 - \frac{1}{2}\sum_{j=1}^{D}\left(1 + \log\sigma_j^2 - \mu_j^2 - \sigma_j^2\right), \tag{S14}$$

where x_i and $\hat{x}_i$ are the i-th element of the observed data $\mathbf{x}$ and the reconstructed data $\hat{\mathbf{x}}$, respectively; μ_j and σ_j are the j-th element of $\boldsymbol{\mu}_{\phi}(\mathbf{x})$ and $\boldsymbol{\sigma}_{\phi}(\mathbf{x})$, respectively.

To train the ELBO with gradient-based optimization, we use the *reparameterization trick* which expresses the random variable $\mathbf{z}$ as a deterministic transformation of the input $\mathbf{x}$ and a Gaussian random variable with zero mean and unit variance $\boldsymbol{\epsilon} \in \mathbb{R}^D$:

$$\mathbf{z} = \boldsymbol{\mu}_{\phi}(\mathbf{x}) + \boldsymbol{\sigma}_{\phi}(\mathbf{x}) \odot \boldsymbol{\epsilon}, \quad \boldsymbol{\epsilon} \sim \mathcal{N}(\mathbf{0}, \mathbf{I}), \tag{S15}$$

where $\odot$ represents the element-wise product.

4 Probabilistic principal component analysis

In PPCA, we assume that the prior distribution over the latent variable $\mathbf{z} \in \mathbb{R}^D$ and the conditional distribution of the observed variable $\mathbf{x} \in \mathbb{R}^V$ given $\mathbf{z}$ follow Gaussian distributions:

$$p(\mathbf{z}) = \mathcal{N}(\mathbf{z}|\mathbf{0}, \mathbf{I}), \quad p(\mathbf{x}|\mathbf{z}) = \mathcal{N}\left(\mathbf{x}|\mathbf{W}\mathbf{z} + \boldsymbol{\mu}, \sigma^2\mathbf{I}\right), \tag{S16}$$

where the matrix $\mathbf{W} \in \mathbb{R}^{V\times D}$ and the vector $\boldsymbol{\mu} \in \mathbb{R}^V$ define the mean of $\mathbf{x}$, and the scalar $\sigma^2 \in \mathbb{R}$ determines the variance of the conditional distribution.

The marginal distribution of the observed data is also a Gaussian:

$$p(\mathbf{x}) = \int p(\mathbf{x}|\mathbf{z})p(\mathbf{z})\,\mathrm{d}(\mathbf{z}) = \mathcal{N}(\mathbf{x}|\boldsymbol{\mu}, \mathbf{C}), \tag{S17}$$

where the covariance matrix $\mathbf{C} \in \mathbb{R}^{V\times V}$ is defined by $\mathbf{C} = \mathbf{W}\mathbf{W}^\top + \sigma^2\mathbf{I}$. Note that $\mathbf{x} = \mathbf{W}\mathbf{z} + \boldsymbol{\mu} + \boldsymbol{\epsilon}$, $\boldsymbol{\epsilon} \sim \mathcal{N}(\mathbf{0}, \sigma^2\mathbf{I})$. Thus, $\mathbb{E}[\mathbf{x}] = \mathbb{E}[\mathbf{W}\mathbf{z} + \boldsymbol{\mu} + \boldsymbol{\epsilon}] = \boldsymbol{\mu}$ and $\mathrm{cov}[\mathbf{x}] = \mathbb{E}[(\mathbf{W}\mathbf{z} + \boldsymbol{\epsilon})(\mathbf{W}\mathbf{z} + \boldsymbol{\epsilon})^\top] = \mathbb{E}[\mathbf{W}\mathbf{z}\mathbf{z}^\top\mathbf{W}^\top] + \mathbb{E}[\boldsymbol{\epsilon}\boldsymbol{\epsilon}^\top] = \mathbf{W}\mathbf{W}^\top + \sigma^2\mathbf{I}$.

Using Bayes' rule, the posterior distribution can be derived as:

$$p(\mathbf{z}|\mathbf{x}) = \mathcal{N}\left(\mathbf{z}|\mathbf{M}^{-1}\mathbf{W}^\top(\mathbf{x} - \boldsymbol{\mu}), \sigma^2\mathbf{M}^{-1}\right), \tag{S18}$$

where the matrix $\mathbf{M} \in \mathbb{R}^{D\times D}$ is given by $\mathbf{M} = \mathbf{W}^\top\mathbf{W} + \sigma^2\mathbf{I}$.

Given a dataset of observed samples $\mathcal{D} = \left\{\mathbf{x}^{(1)}, \ldots, \mathbf{x}^{(i)}, \ldots, \mathbf{x}^{(N)}\right\}$, the corresponding log likelihood function can be written as:

$$\ln p\left(\mathcal{D}|\boldsymbol{\mu}, \mathbf{W}, \sigma^2\right) = \sum_{i=1}^{N} \ln p\left(\mathbf{x}^{(i)}|\boldsymbol{\mu}, \mathbf{W}, \sigma^2\right) \tag{S19}$$

$$= -\frac{NV}{2}\ln(2\pi) - \frac{N}{2}\ln|\mathbf{C}| - \frac{1}{2}\sum_{i=1}^{N}\left(\mathbf{x}^{(i)} - \boldsymbol{\mu}\right)^\top \mathbf{C}^{-1}\left(\mathbf{x}^{(i)} - \boldsymbol{\mu}\right). \tag{S20}$$

Using maximum likelihood estimation, we obtain the model parameter $\boldsymbol{\mu} = \bar{\mathbf{x}} = \frac{1}{N}\sum_{i=1}^{N}\mathbf{x}^{(i)}$ by setting the derivative of the log likelihood with respect to $\boldsymbol{\mu}$ equal to zero.

We implement the expectation-maximization (EM) algorithm to estimate the model parameters $\mathbf{W}$ and σ^2. In the E step, we use the old parameter values to evaluate the expectation of the complete-data log

likelihood with respect to the posterior distribution:

$$\mathbb{E}\left[\ln p\left(\mathcal{D}, \mathcal{Z} | \boldsymbol{\mu}, \mathbf{W}, \sigma^2\right)\right] = -\sum_{i=1}^{N}\left(\frac{V}{2}\ln\left(2\pi\sigma^2\right) + \frac{1}{2}\operatorname{tr}\left(\mathbb{E}\left[\mathbf{z}^{(i)}\mathbf{z}^{(i)\top}\right]\right) + \frac{1}{2\sigma^2}\left\|\mathbf{x}^{(i)} - \boldsymbol{\mu}\right\|^2 \right.$$
$$\left. - \frac{1}{\sigma^2}\mathbb{E}\left(\mathbf{z}^{(i)}\right)^\top \mathbf{W}^\top\left(\mathbf{x}^{(i)} - \boldsymbol{\mu}\right) + \frac{1}{2\sigma^2}\operatorname{tr}\left(\mathbb{E}\left[\mathbf{z}^{(i)}\mathbf{z}^{(i)\top}\right]\mathbf{W}^\top\mathbf{W}\right) + \frac{D}{2}\ln(2\pi)\right), \tag{S21}$$

$$\mathbb{E}\left[\mathbf{z}^{(i)}\right] = \mathbf{M}^{-1}\mathbf{W}^\top\left(\mathbf{x}^{(i)} - \bar{\mathbf{x}}\right), \tag{S22}$$

$$\mathbb{E}\left[\mathbf{z}^{(i)}\mathbf{z}^{(i)\top}\right] = \sigma^2\mathbf{M}^{-1} + \mathbb{E}\left[\mathbf{z}^{(i)}\right]\mathbb{E}\left[\mathbf{z}^{(i)}\right]^\top, \tag{S23}$$

where $\mathbf{z}^{(i)}$ is the i-th element in the set of latent variables $\mathcal{Z} = \left\{\mathbf{z}^{(1)}, \ldots, \mathbf{z}^{(i)}, \ldots, \mathbf{z}^{(N)}\right\}$.

In the M step, we maximize the the expected complete-data log likelihood with respect to $\mathbf{W}$ and σ^2, yielding the new parameter values:

$$\mathbf{W}_{\text{new}} = \left[\sum_{i=1}^{N}\left(\mathbf{x}^{(i)} - \bar{\mathbf{x}}\right)\mathbb{E}\left[\mathbf{z}^{(i)}\right]^\top\right]\left[\sum_{i=1}^{N}\mathbb{E}\left[\mathbf{z}^{(i)}\mathbf{z}^{(i)\top}\right]\right]^{-1}, \tag{S24}$$

$$\sigma^2_{\text{new}} = \frac{1}{VN}\sum_{i=1}^{N}\left(\left\|\mathbf{x}^{(i)} - \bar{\mathbf{x}}\right\|^2 - 2\mathbb{E}\left[\mathbf{z}^{(i)}\right]^\top\mathbf{W}_{\text{new}}^\top\left(\mathbf{x}^{(i)} - \bar{\mathbf{x}}\right) + \operatorname{tr}\left(\mathbb{E}\left[\mathbf{z}^{(i)}\mathbf{z}^{(i)\top}\right]\mathbf{W}_{\text{new}}^\top\mathbf{W}_{\text{new}}\right)\right). \tag{S25}$$

We iteratively alternate between the E step and the M step, until the parameter values converge. For more details, we refer readers to Tipping and Bishop, 1999 or Chapter 16, Bishop and Bishop, 2023.

5 Identifiable variational autoencoders

In iVAE, we consider the following conditional generative model:

$$\mathbf{x} = \mathbf{f}(\mathbf{z}) + \boldsymbol{\epsilon}, \tag{S26}$$

$$p_{\boldsymbol{\theta}}(\mathbf{x}, \mathbf{z}|\mathbf{u}) = p_{\mathbf{f}}(\mathbf{x}|\mathbf{z}) p_{\mathbf{T},\boldsymbol{\lambda}}(\mathbf{z}|\mathbf{u}), \tag{S27}$$

$$p_{\mathbf{f}}(\mathbf{x}|\mathbf{z}) = p_{\boldsymbol{\epsilon}}\left(\mathbf{x} - \mathbf{f}(\mathbf{z})\right), \tag{S28}$$

$$p_{\mathbf{T},\boldsymbol{\lambda}}(\mathbf{z}|\mathbf{u}) = \prod_{i=1}^{D} \frac{Q_i(z_i)}{Z_i(\mathbf{u})} \exp\left[\sum_{j=1}^{s} T_{i,j}(z_i)\lambda_{i,j}(\mathbf{u})\right], \tag{S29}$$

where $\mathbf{x} \in \mathbb{R}^V$ is the observed data variable, $\mathbf{u} \in \mathbb{R}^U$ is the observed auxiliary variable, $\mathbf{z} \in \mathbb{R}^D$ is the latent variable, $\boldsymbol{\epsilon} \in \mathbb{R}^V$ is a noise variable with probability density function $p_{\boldsymbol{\epsilon}}(\boldsymbol{\epsilon})$, $\boldsymbol{\theta} = (\mathbf{f}, \mathbf{T}, \boldsymbol{\lambda})$ is a set of parameters of the generative model, and $\mathbf{f} : \mathbb{R}^D \to \mathbb{R}^V$ is a nonlinear mixing function. Since our main interest lies in psychiatric characteristics, we use the one-hot encoded diagnostic label as the auxiliary variable $\mathbf{u}$ in the iVAE ($U = 2$). We assume that the prior on the latent variable $p_{\mathbf{T},\boldsymbol{\lambda}}(\mathbf{z}|\mathbf{u})$ is conditionally independent, and each z_i follows a univariate exponential family distribution given the auxiliary variable $\mathbf{u}$, where Q_i is the base measure, $Z_i(\mathbf{u})$ is the normalizing constant, $\mathbf{T}_i = (T_{i,1}, \ldots, T_{i,s})$ are the sufficient statistics, $\boldsymbol{\lambda}_i(\mathbf{u}) = (\lambda_{i,1}(\mathbf{u}), \ldots, \lambda_{i,s}(\mathbf{u}))$ are the parameters depending on $\mathbf{u}$, and s is the dimension of each sufficient statistic.

Similar to VAE, the iVAE objective function aims to learn the parameters $(\boldsymbol{\theta}, \boldsymbol{\phi})$ that maximize the data generation likelihood by maximizing the ELBO:

$$\mathcal{L}_{\text{iVAE}} = \max_{\boldsymbol{\theta},\boldsymbol{\phi}} \log p_{\boldsymbol{\theta}}(\mathbf{x}|\mathbf{u}) - D_{\text{KL}}\left(q_{\boldsymbol{\phi}}(\mathbf{z}|\mathbf{x}, \mathbf{u}) || p_{\boldsymbol{\theta}}(\mathbf{z}|\mathbf{x}, \mathbf{u})\right), \tag{S30}$$

where $p_{\boldsymbol{\theta}}(\mathbf{x}|\mathbf{u}) = \int p_{\boldsymbol{\theta}}(\mathbf{x}, \mathbf{z}|\mathbf{u})\, \mathrm{d}\mathbf{z}$ is the conditional marginal distribution of the observed data, and $q_{\boldsymbol{\phi}}(\mathbf{z}|\mathbf{x}, \mathbf{u})$ is the variational posterior of its true posterior $p_{\boldsymbol{\theta}}(\mathbf{z}|\mathbf{x}, \mathbf{u})$.

For more information, please refer to Khemakhem et al., 2020.

6 Model architecture search

6.1 VAE architecture search

We evaluated four VAE architectures based on multilayer perceptrons (MLPs). Each architecture has a different set of output units in the VAE encoder and the corresponding symmetric output units in the VAE decoder. The number of output units in each encoder layer is listed in Table S4. In each layer, we used a Leaky Rectified Linear Unit (Leaky ReLU) activation function (Maas et al., 2013) with a negative slope of 0.5.

For each dataset (FBIRN or ABIDE I) and each data type (sFNC or dFNC), we evaluated four candidate model architectures. For each architecture, we used 10 different random seeds to initialize the model weights. As shown in Figure S1, smaller models with 2 or 3 layers achieved optimal performance for sFNC data, while larger models with 5 or 7 layers worked better for dFNC data. Table S5 summarizes the selected VAE architectures based on training performance across 10 runs.

Table S4: **Evaluated VAE architectures.**

Number of layers	Output units in each encoder layer
2	64, 16
3	128, 64, 32
5	256, 128, 64, 32, 16
7	512, 256, 128, 64, 32, 16, 8

Table S5: **Selected VAE architectures.**

Dataset	Data type	Number of layers	Output units in each encoder layer
FBIRN	sFNC	3	128, 64, 32
FBIRN	dFNC	7	512, 256, 128, 64, 32, 16, 8
ABIDE I	sFNC	2	64, 16
ABIDE I	dFNC	5	256, 128, 64, 32, 16

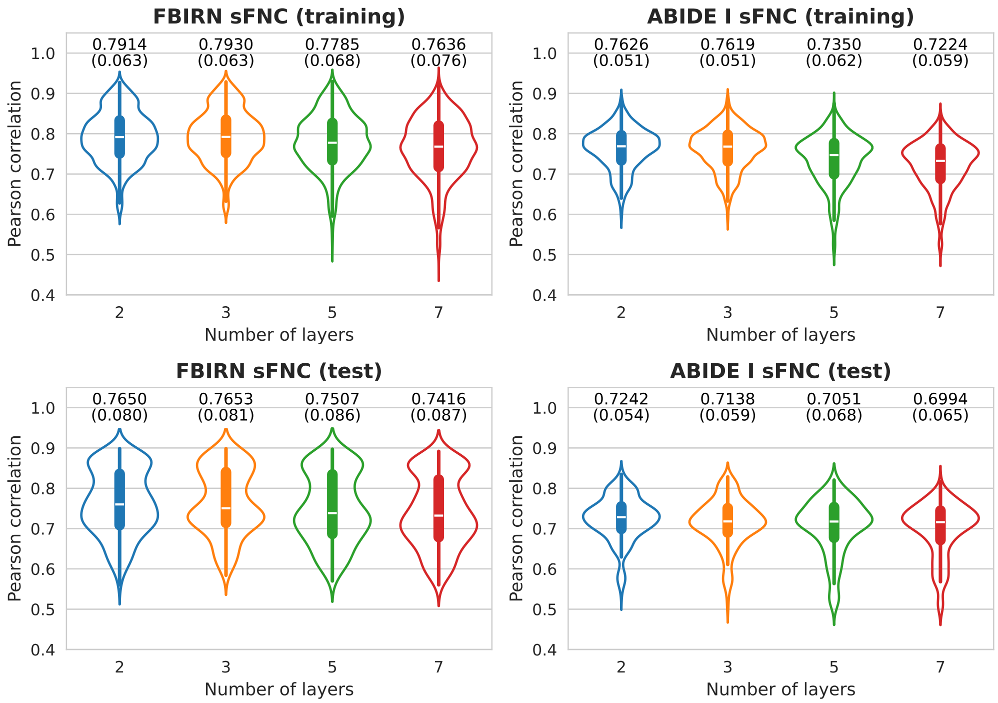

(a) sFNC data.

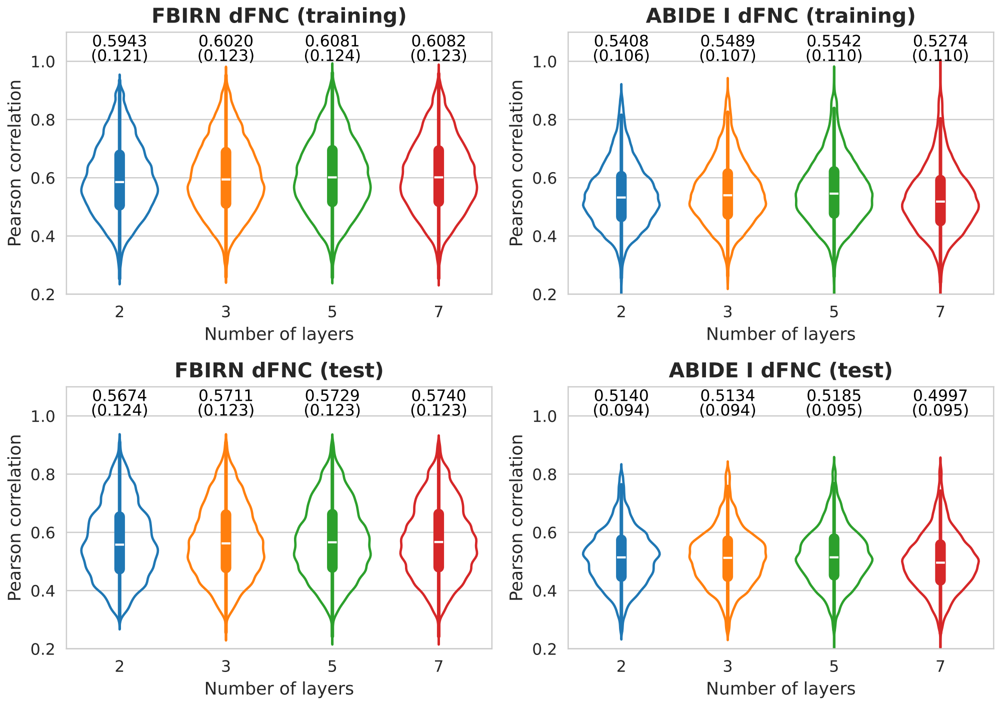

(b) dFNC data.

Figure S1: **VAE architecture search.** Each violin plot shows a symmetric kernel density estimate of Pearson correlations between original and generated sFNC matrices across 10 random seeds, along with the quartiles represented by a box plot.

6.2 iVAE architecture search

We evaluated 12 MLP-based encoder-decoder architectures, varying the number of layers $(2, 3, 4)$ and hidden dimensions $(16, 32, 64, 96)$. According to the training performance (Figure S2), we selected the iVAE model with 3 layers and 16 hidden units for FBIRN, and the model with 2 layers and 32 hidden units for ABIDE I.

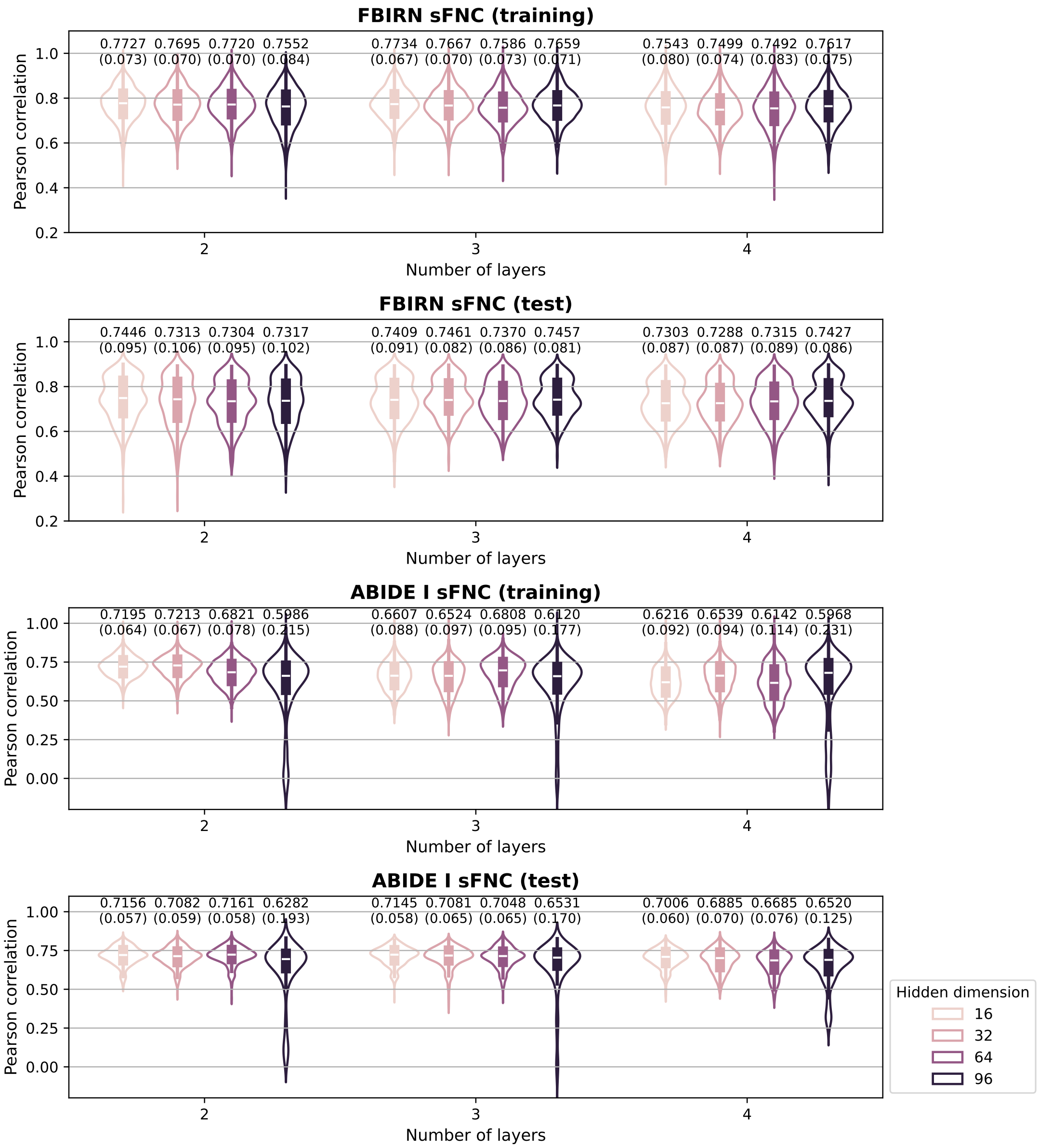

Figure S2: **iVAE architecture search for sFNC data.** Each violin plot shows a symmetric kernel density estimate of Pearson correlations between original and generated sFNC matrices across 10 random seeds, along with the quartiles represented by a box plot.

7 Confound effects on latent representations

To assess the robustness of the learned representation to potential confounding variables (e.g., age, sex, and collection site), we regressed these variables out of the latent representation ($\mathbf{z}$) using linear regression. For the FBIRN dataset, we constructed a confound matrix $\mathbf{C} = \left[\mathbf{1}, \mathbf{c}_{\text{age}}, \mathbf{c}_{\text{sex}}, \mathbf{c}_{\text{site}}\right] \in \mathbb{R}^{N\times 9}$, where $\mathbf{1} \in \mathbb{R}^N$ is a column vector of ones, $\mathbf{c}_{\text{age}} \in \mathbb{R}^N$ is the demeaned linear age vector, $\mathbf{c}_{\text{sex}} \in \mathbb{R}^N$ is the binary sex vector (0: female; 1: male), and $\mathbf{c}_{\text{site}} \in \mathbb{R}^{N\times 6}$ represents the one-hot encoded label of seven sites. For the ABIDE I dataset, we constructed a similar confound matrix $\mathbf{C} = \left[\mathbf{1}, \mathbf{c}_{\text{age}}, \mathbf{c}_{\text{sex}}, \mathbf{c}_{\text{site}}\right] \in \mathbb{R}^{N\times 5}$, where $\mathbf{c}_{\text{site}} \in \mathbb{R}^{N\times 2}$ encodes the site information ($[0,0]$: NYU; $[1,0]$: UCLA; $[0,1]$: USM). The latent representation after regressing out the confounding variables ($\mathbf{z}'$) was calculated as follows:

$$\mathbf{z}' = \mathbf{z} - \mathbf{C}\left(\mathbf{C}^\top \mathbf{C}\right)^{-1} \mathbf{C}^\top \mathbf{z}. \tag{S31}$$

Next, we calculated the randomized dependence coefficient (RDC) (Lopez-Paz et al., 2013), a measure of nonlinear dependence, between the representations before and after regression. An RDC value is bounded between 0 and 1, with higher values indicating greater similarity.

As shown in Figure S3, the high RDC values along the diagonal (FBIRN: $0.978, 0.962$; ABIDE I: $0.976, 0.955$) indicated high similarity between the representations before and after regression, suggesting that the learned representations appeared to be largely insensitive to the examined confounding factors.

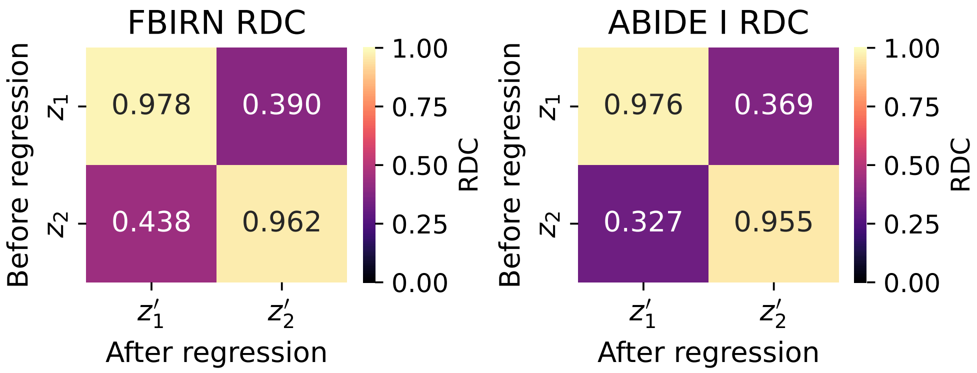

Figure S3: **Randomized dependence coefficient (RDC) between the latent representations before and after regressing out the confounding variables.** High diagonal RDC values indicated strong similarity between representations before and after regression, suggesting that the learned representations were largely unaffected by the examined confounding factors.

8 sFNC visualization

8.1 FBIRN

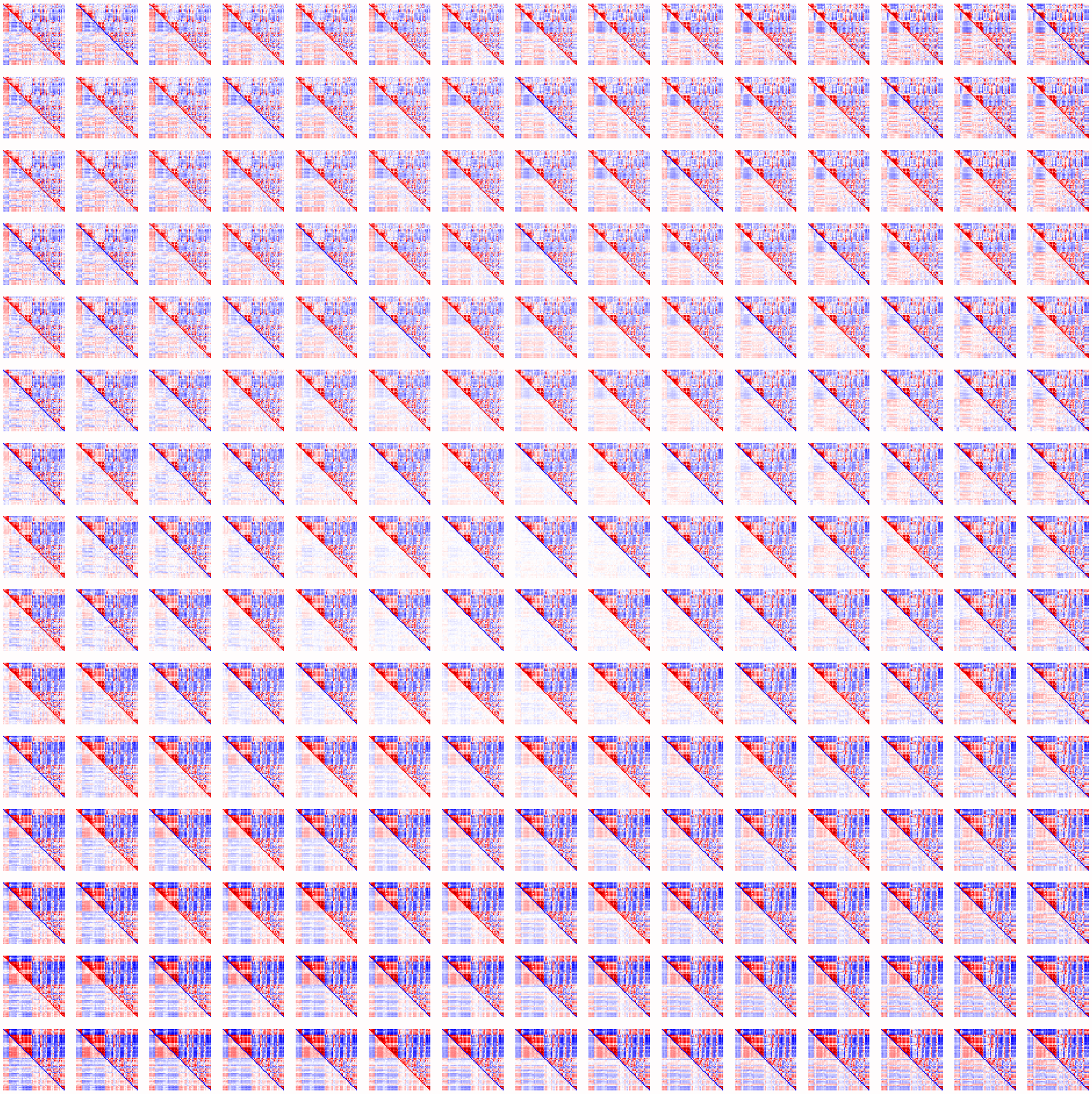

Figure S4: **FBIRN generated sFNC (training).**

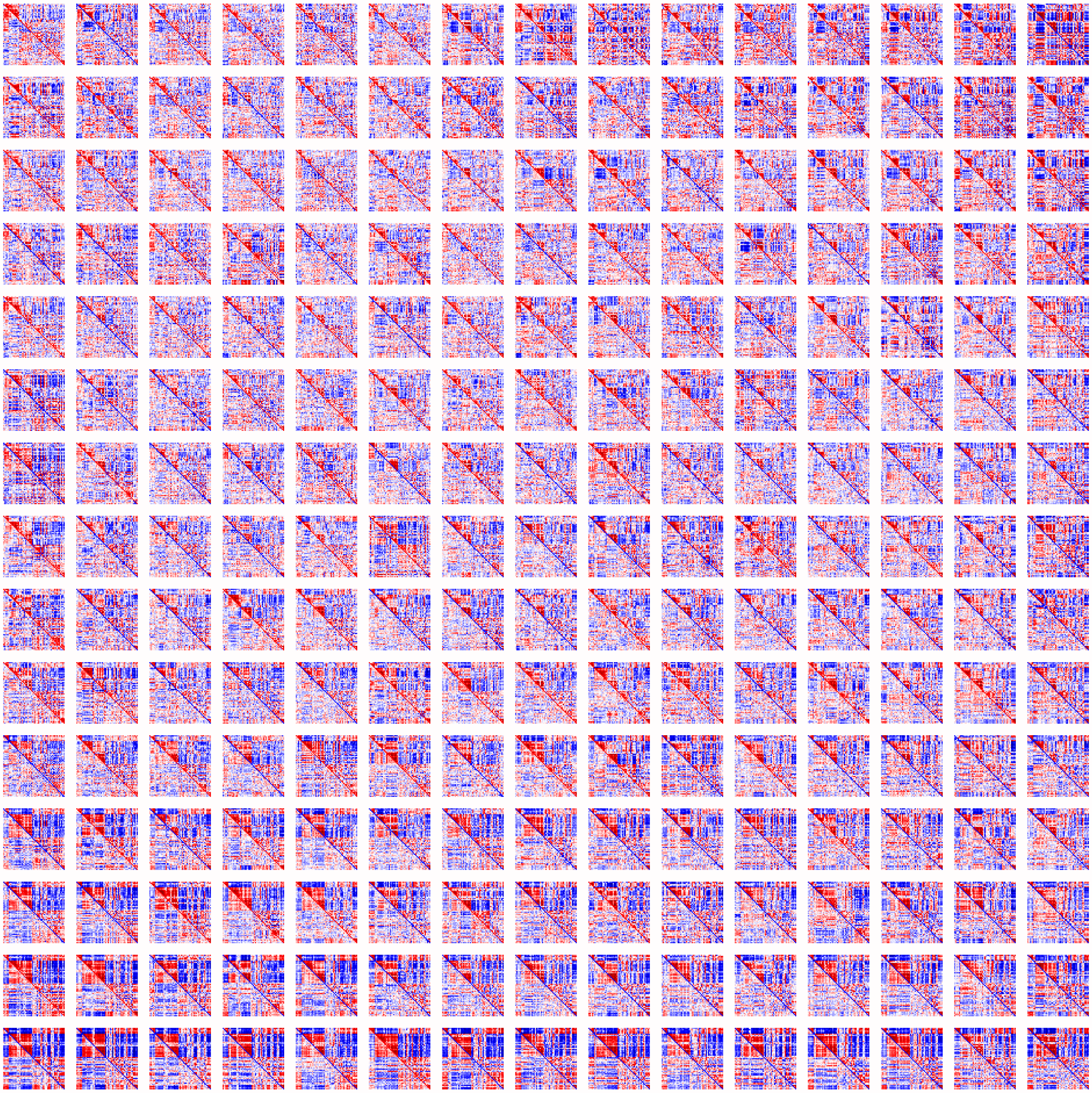

Figure S5: **FBIRN original sFNC (training).**

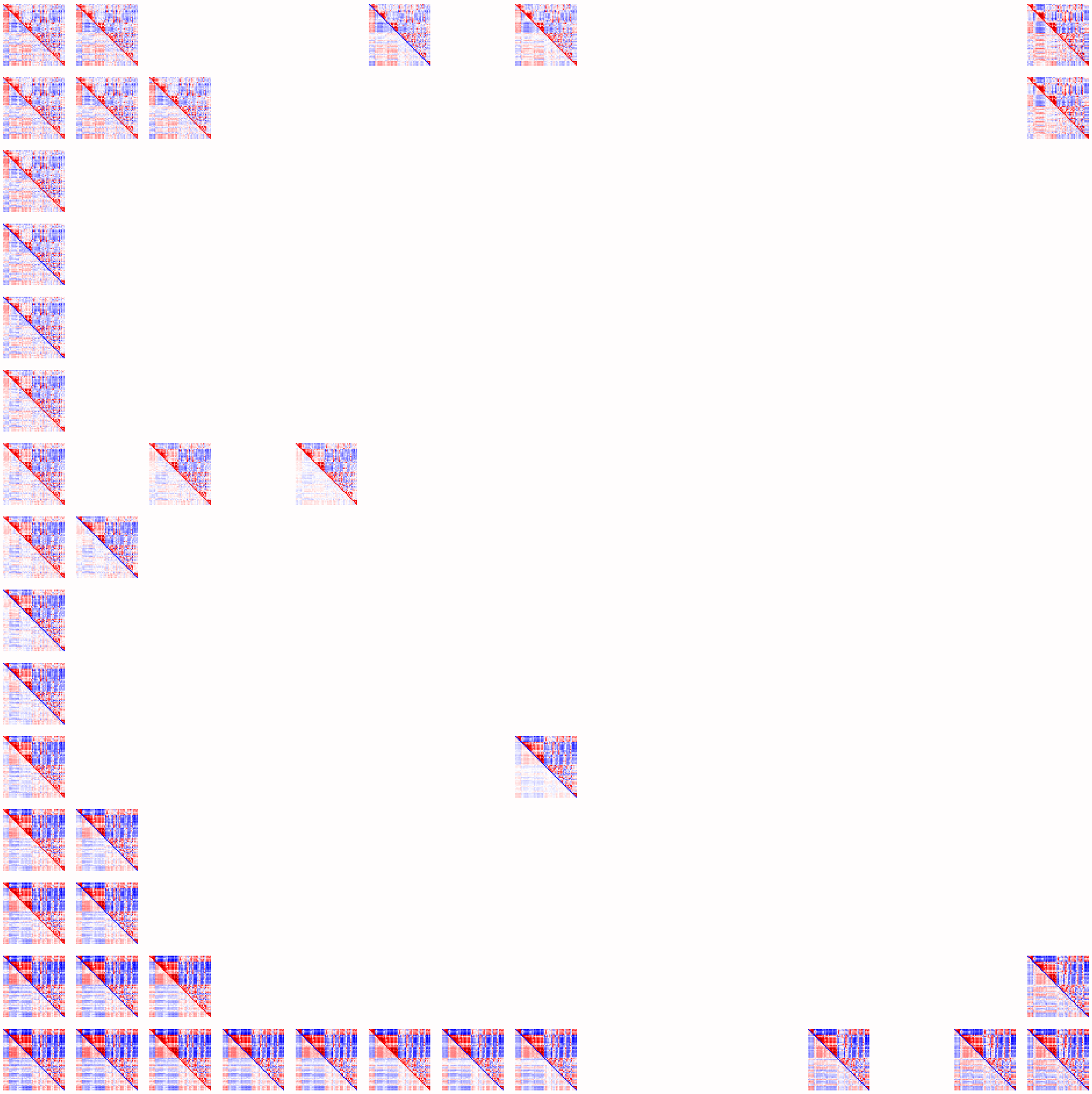

Figure S6: **FBIRN generated sFNC (test).**

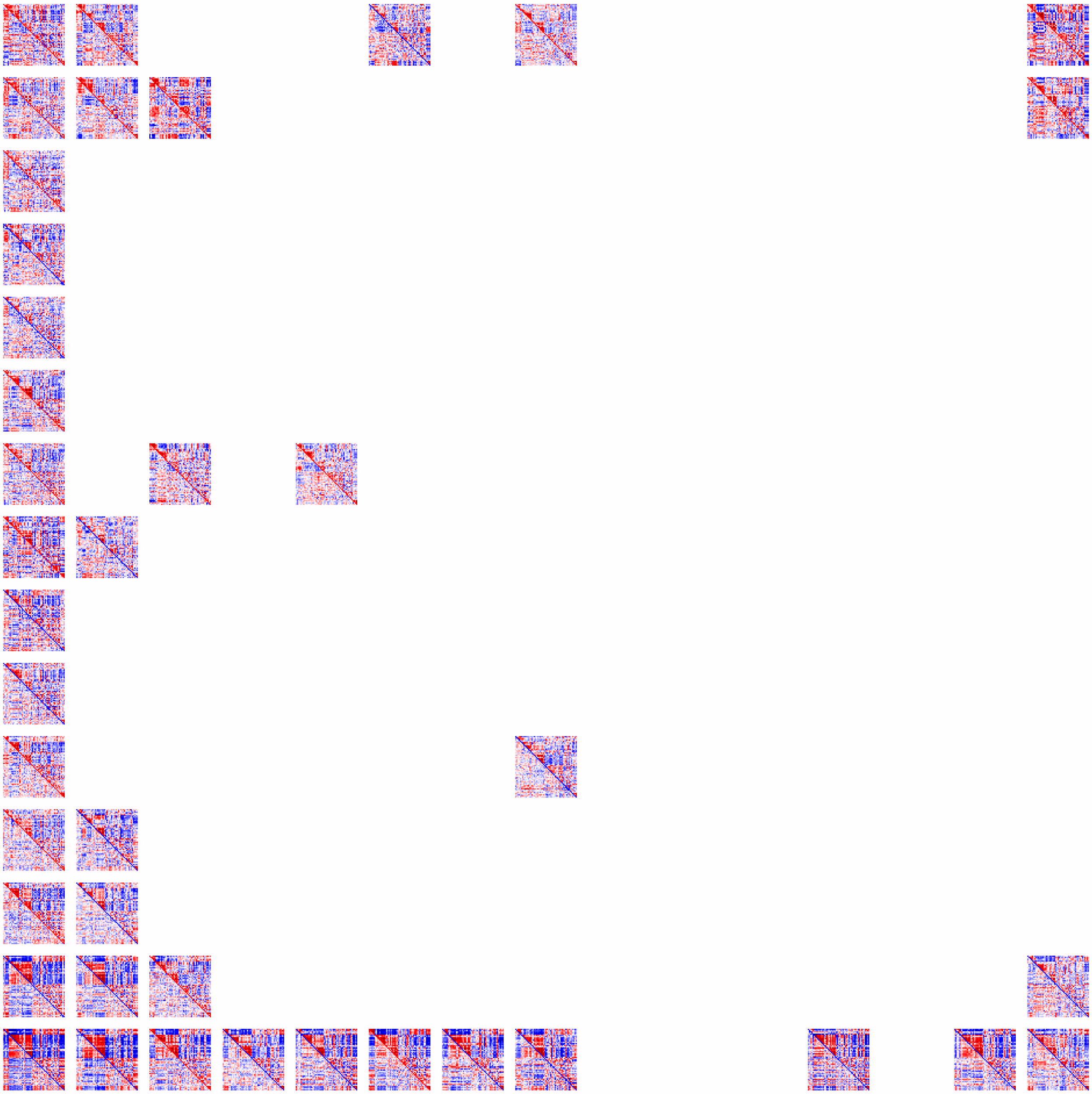

Figure S7: **FBIRN original sFNC (test).**

8.2 ABIDE I

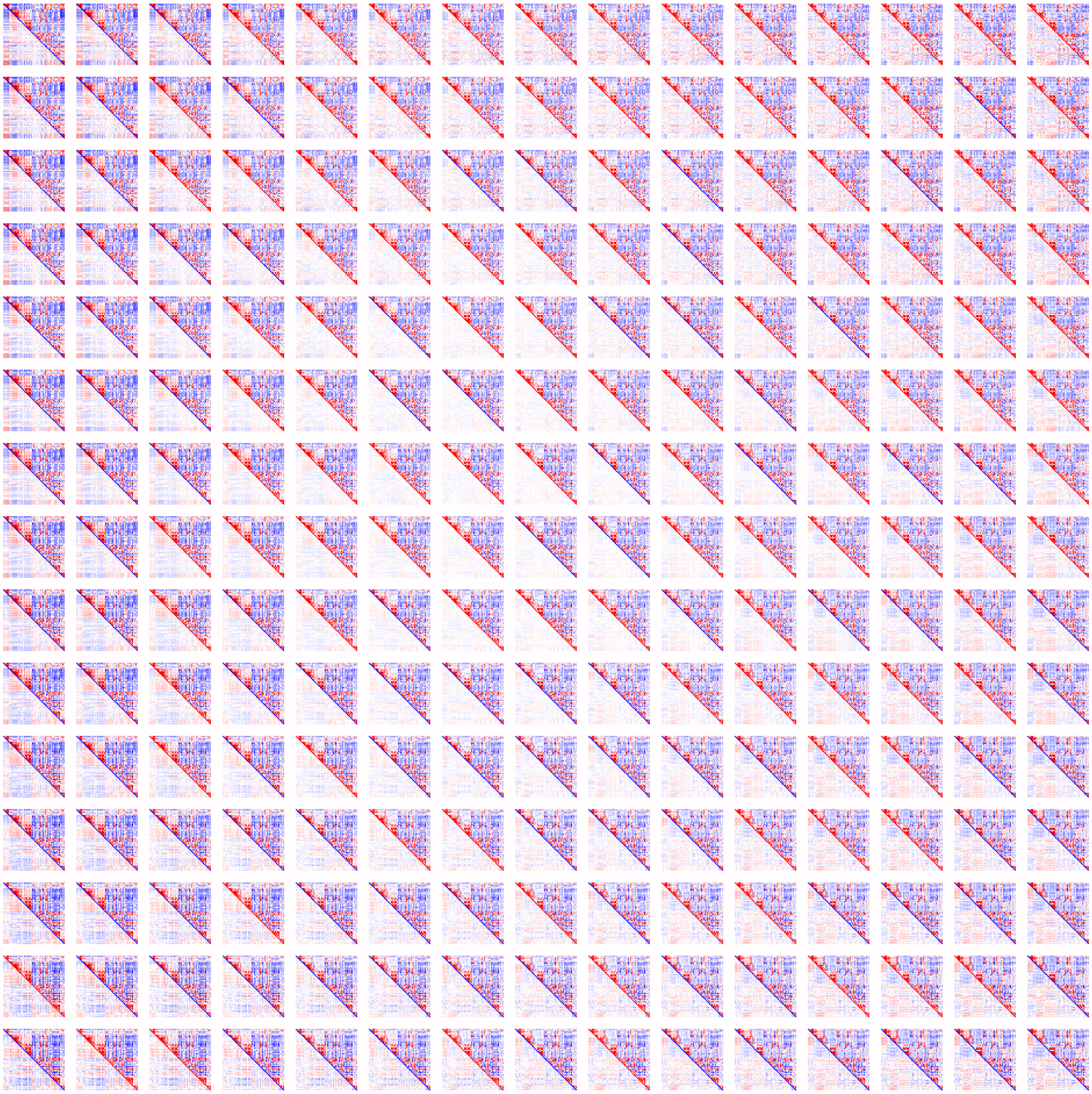

Figure S8: **ABIDE I generated sFNC (training).**

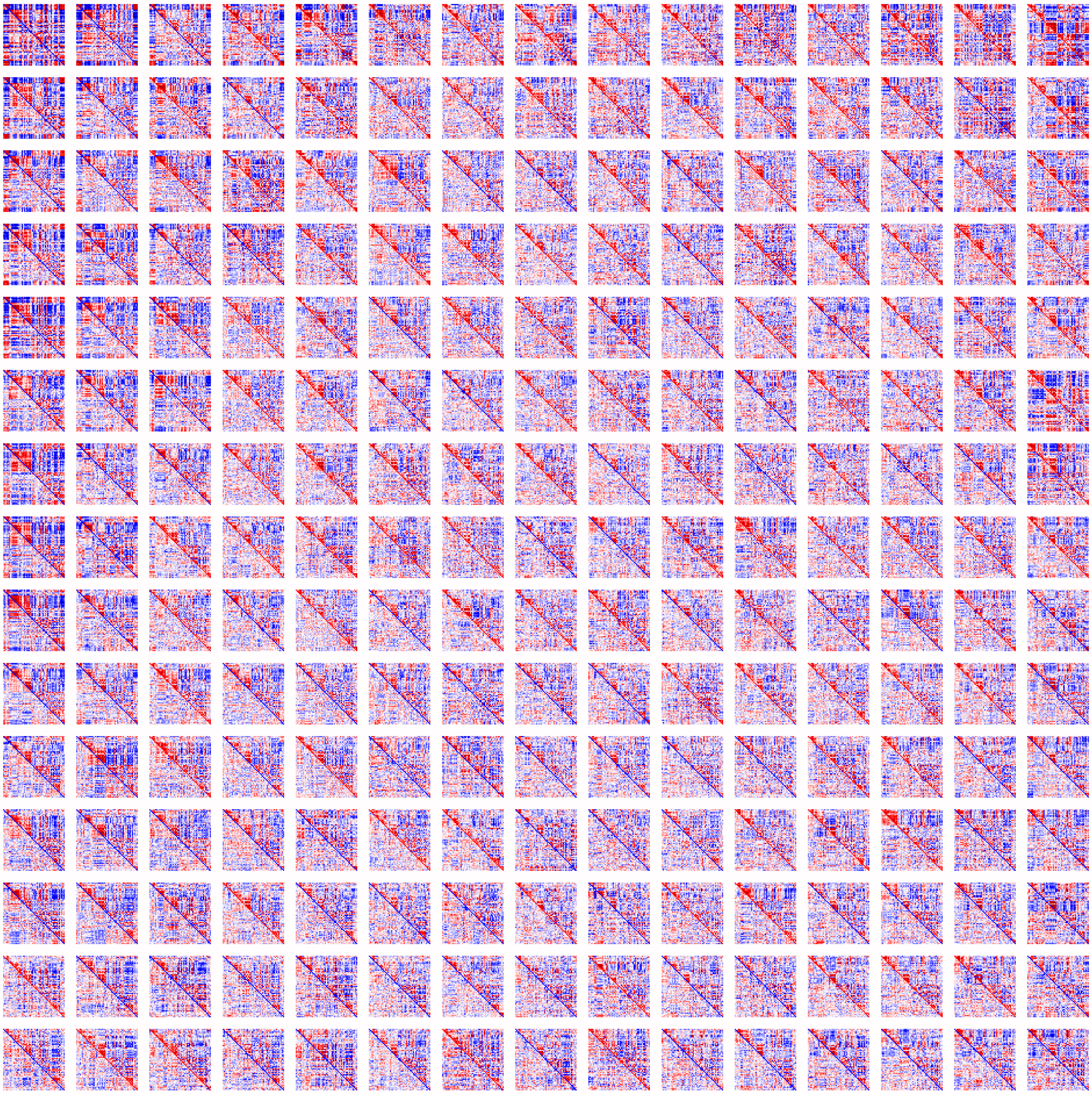

Figure S9: **ABIDE I original sFNC (training).**

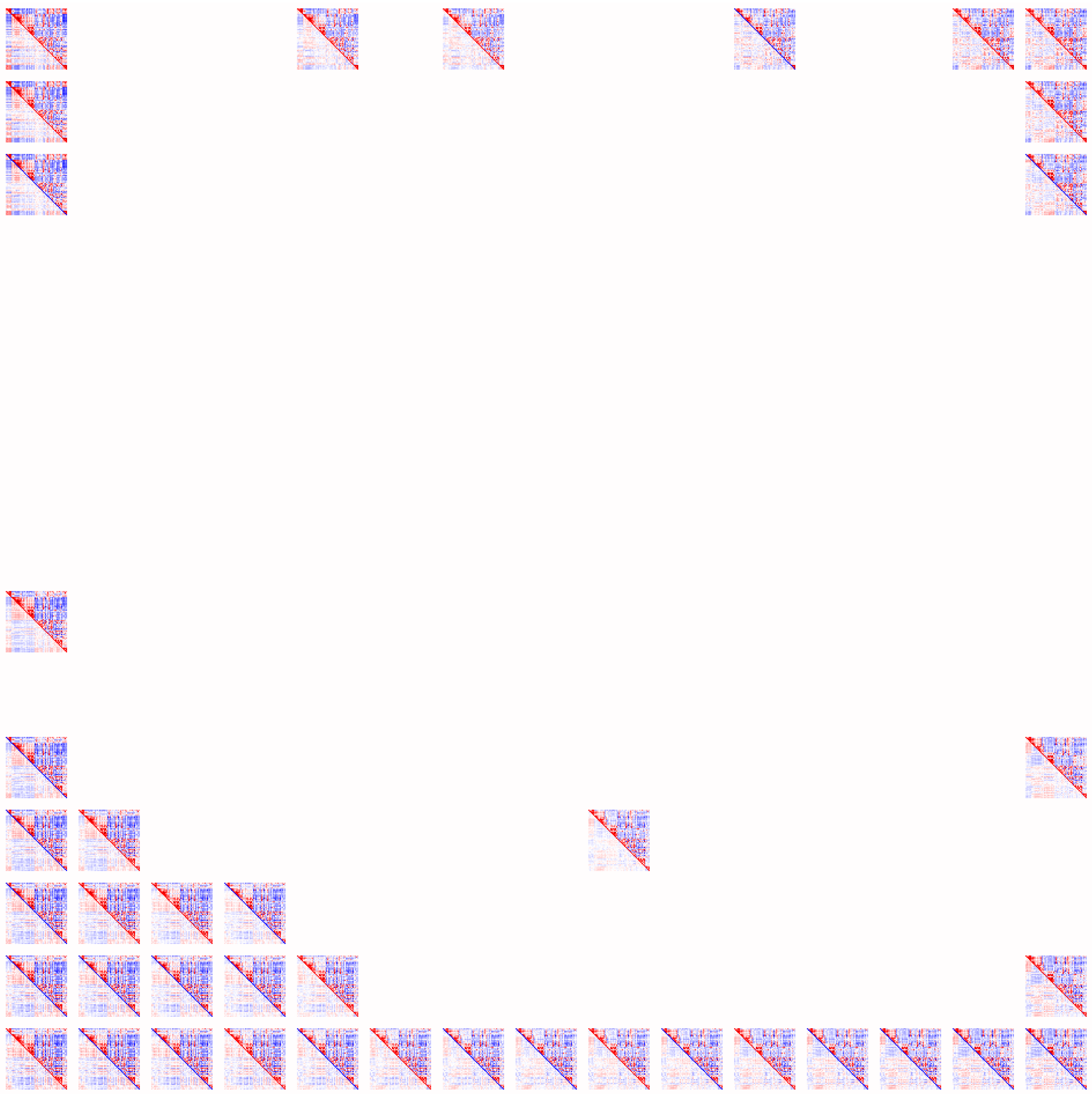

Figure S10: **ABIDE I generated sFNC (test).**

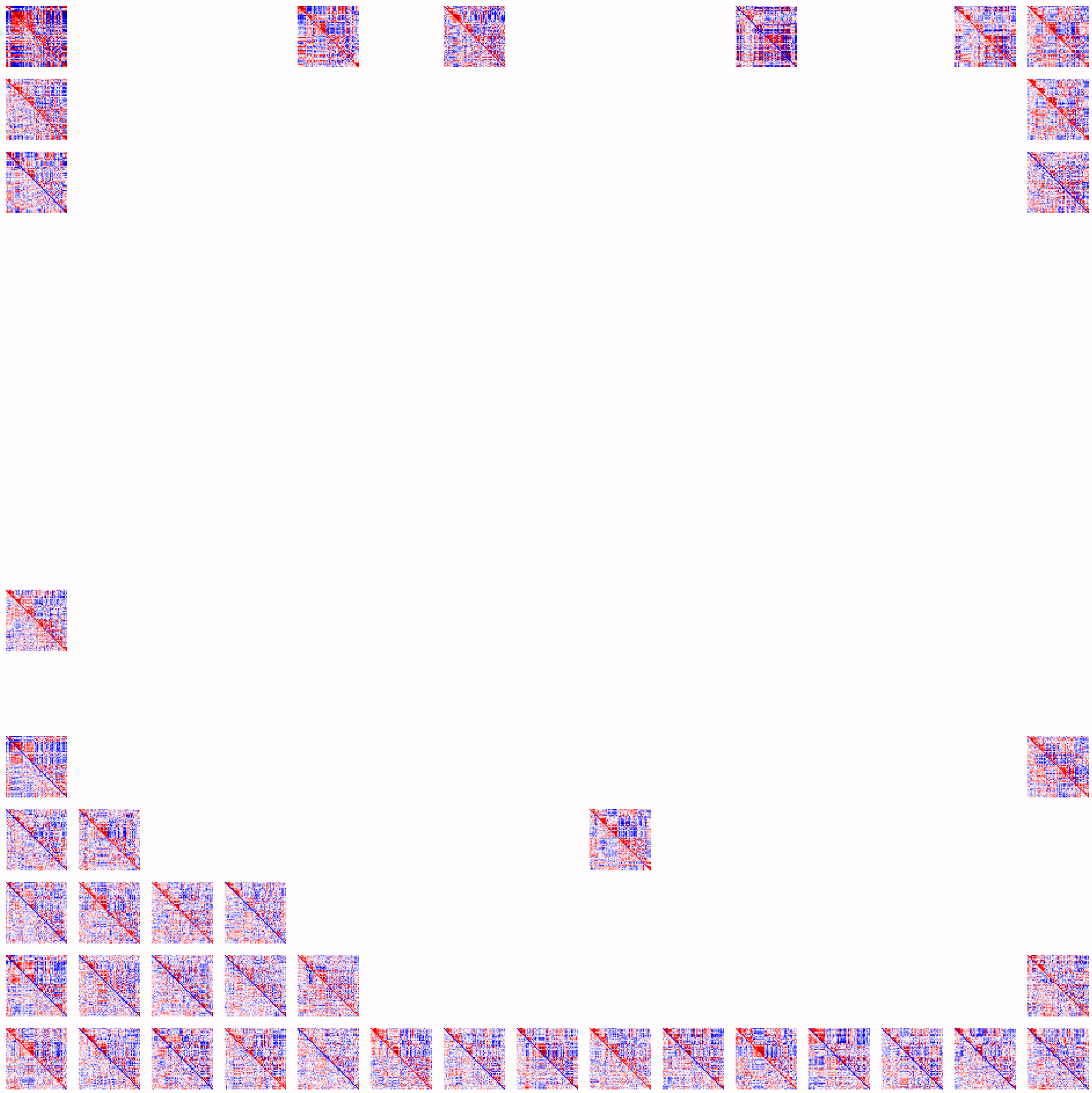

Figure S11: **ABIDE I original sFNC (test).**

9 Group differences in sFNC variability

To quantify heterogeneity in sFNC, we computed the element-wise standard deviation of sFNC for each group and each dataset (Figure S12). In FBIRN, the average standard deviation was slightly higher in the patient group (0.201) than in the control group (0.199). Similarly, in ABIDE I, the patient group also showed a marginally higher average standard deviation (0.185) compared with controls (0.183). These results indicate slightly greater sFNC variability in the patient groups across both datasets.

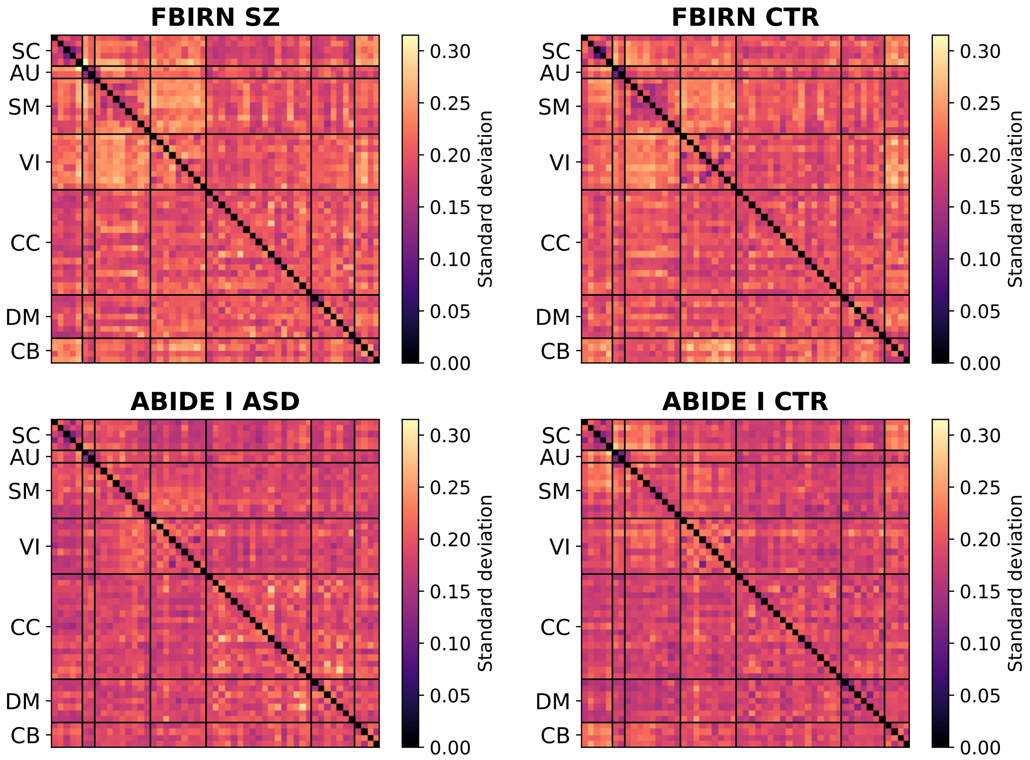

Figure S12: **sFNC element-wise standard deviation.** In both the FBIRN and ABIDE I datasets, the average element-wise standard deviation in the patient group was marginally higher than that in the control group, indicating slightly greater sFNC variability in the patient group.

10 Similarity measured by mean squared error

The mean squared error (MSE) was used to measure the similarity between generated and original sFNC matrices. The median MSEs for the FBIRN training set, FBIRN test set, ABIDE I training set, and ABIDE I test set were 0.031, 0.034, 0.029, and 0.034, respectively. These results suggest that the generated sFNC matrices closely matched the original ones, as measured by MSE.

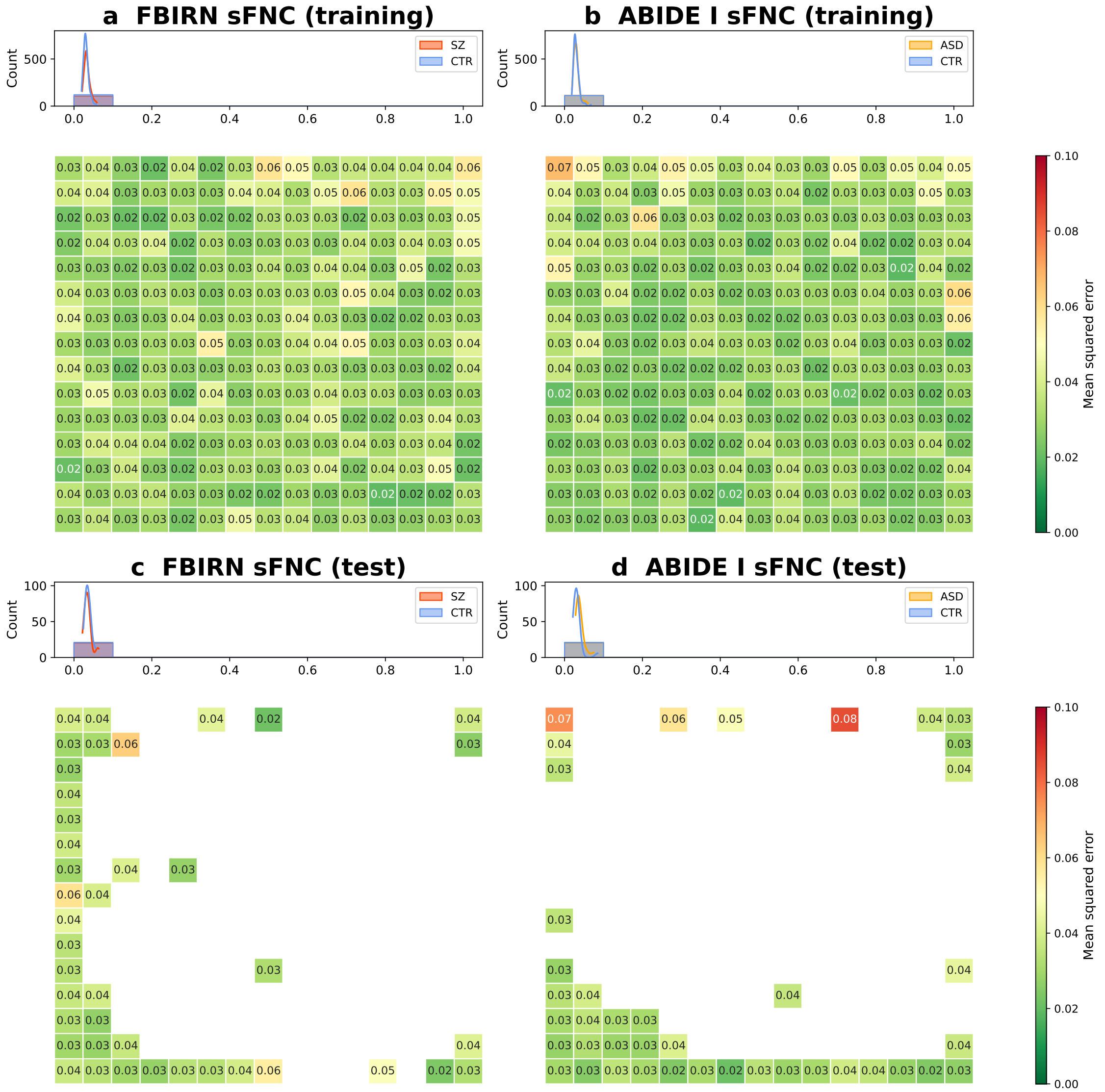

Figure S13: **Mean squared errors between generated and original sFNC matrices.** (a) FBIRN training set. (b) ABIDE I training set. (c) FBIRN test set. (d) ABIDE I test set. Each panel shows mean squared errors (MSEs) between individual generated and original sFNC matrices on the 2D grid and the corresponding group-specific histogram distributions. Low MSEs across all panels suggest a high degree of correspondence between generated and original sFNC matrices.

11 Subgroup analysis using two- and three-dimensional latent representations

We examined how the dimensionality of the VAE latent space affects subgroup identification within the SZ patient cohort. First, we trained two VAEs to learn latent representations, one with a two-dimensional (2D) latent space and one with a three-dimensional (3D) latent space. Second, for each set of latent representations, we applied k-means clustering, varying the number of clusters k from 2 to 9. Third, we selected the optimal number of clusters using the elbow criterion based on the L_2 loss. Finally, we assessed whether pairs of clusters differed significantly in subject measures, including PANSS positive and negative scores, CMINDS cognitive scores, age, and gender.

Based on the elbow criterion, four clusters were identified for 2D representations and six clusters for 3D representations (Figure S14). Figures S15 and S18 show the resulting subgroup (cluster) assignments and the corresponding average sFNC pattern for each subgroup within each diagnostic group. Figures S16 and S19 illustrate the corresponding p-values for subject measures between subgroup pairs. Figures S17 and S20 then display distributions of subject measures across subgroups. For both 2D and 3D representations, fifteen subgroup pairs exhibited statistically significant differences ($p < 0.05$, Mann–Whitney U test), suggesting that the higher-dimensional (3D) latent space did not yield additional meaningful subgroups compared with the 2D latent space.

Furthermore, we examined FNC patterns across four subgroups derived from 2D latent representations (Figure S15). Table S6 summarizes pairwise subgroup differences in subject measures and FNC patterns. Subgroups 1 and 2 differed significantly in age and attention scores ($p < 0.05$, Mann–Whitney U test), with associated FNC changes primarily involving sensorimotor and cognitive control networks, including SC-SM, AU-CC, SM-VI, SM-DM, and CC-CB domains ($p < 0.0001$, Wilcoxon signed-rank test with Bonferroni correction). Subgroups 1 and 3 showed significant differences in age, visual learning scores, and composite scores. Statistically significant differences were observed in both within-network alterations (SM, VI) and between-network interactions. Subgroups 1 and 4 differed significantly in visual learning scores, with FNC differences dominated by visual network connectivity (VI-VI, VI-CB, VI-SM, VI-SC) and subcortical, sensorimotor, and cerebellar interactions (SC-SM, SC-CB, SM-CB). Subgroups 2 and 3 were significantly different in visual learning and reasoning scores, with FNC differences spanning within-network connectivity (SM, VI, and CC) and between-network interactions involving subcortical, sensorimotor, visual, and cerebellar-related connections. Subgroups 2 and 4 differed in working memory and visual learning scores, particularly among subcortical, sensorimotor, visual, and cerebellar domains. Subgroups 3 and 4 exhibited multiple cognitive score differences, including speed of processing, working

memory, verbal learning, visual learning, and composite scores, with FNC differences mainly involving sensorimotor and visual networks. Table S7 summarizes pairwise subgroup differences in subject measures and FNC patterns using 3D latent representations.

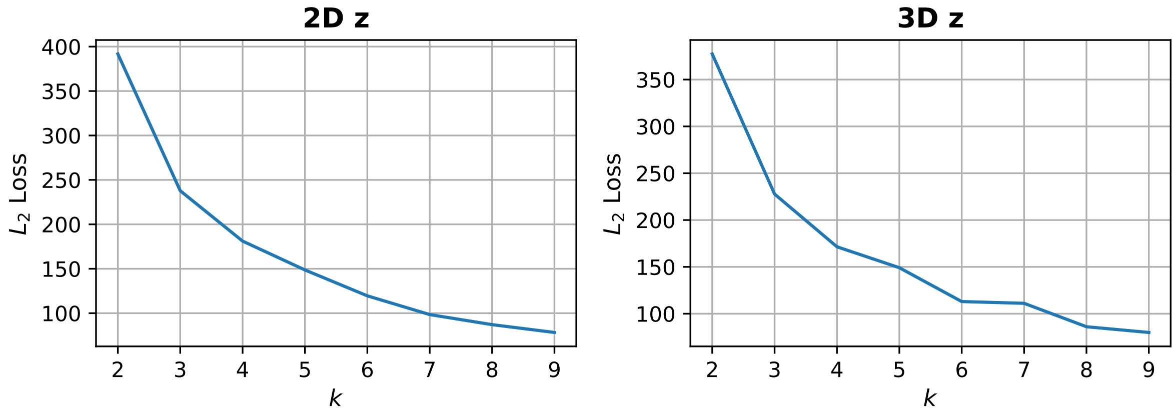

Figure S14: k**-means clustering** L_2 **loss with respect to different** k **values (2D vs 3D).** According to the elbow criterion, four clusters were identified for 2D representations and six clusters for 3D representations.

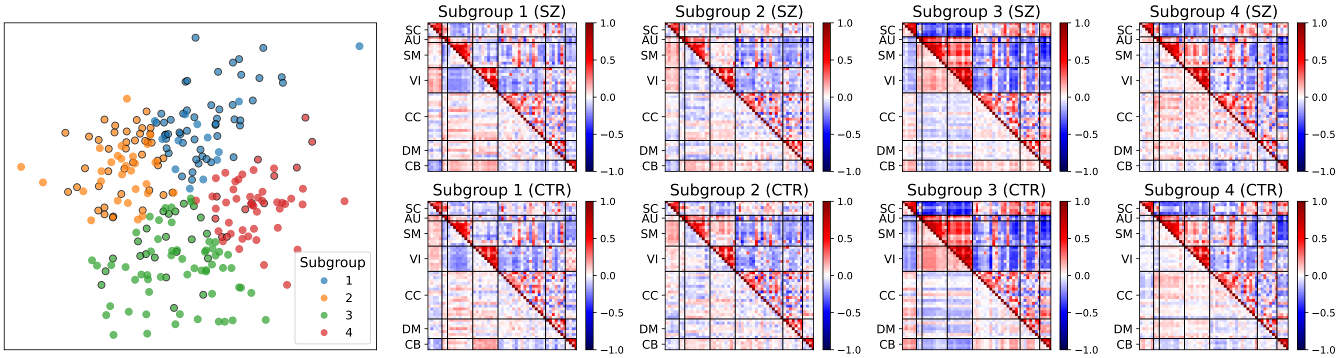

Figure S15: **Subgroups based on 2D latent representations.** In the scatter plot, SZ patients are highlighted in black circles, while controls are shown in their cluster colors. The average sFNC pattern is shown for each of the four k-means subgroups (clusters) and for each of the two diagnostic groups (SZ patients and controls). Subgroups were sorted by the increasing proportion of controls among all subjects.

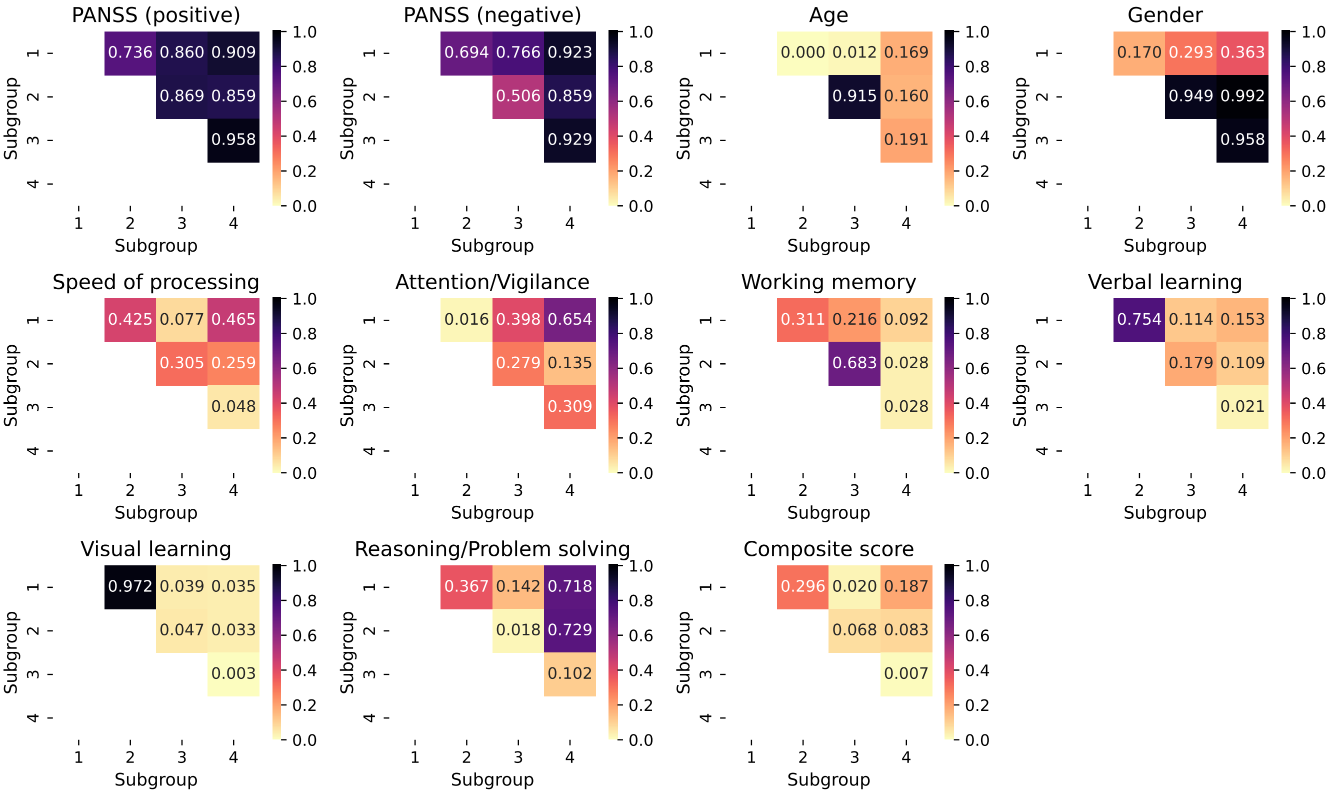

Figure S16: **Statistical significance of each score type between two SZ subgroups (2D latent representations).** The heatmap shows p-values for each score type between all pairs of subgroups (Mann–Whitney U test). Fifteen cluster pairs exhibited statistically significant differences ($p < 0.05$).

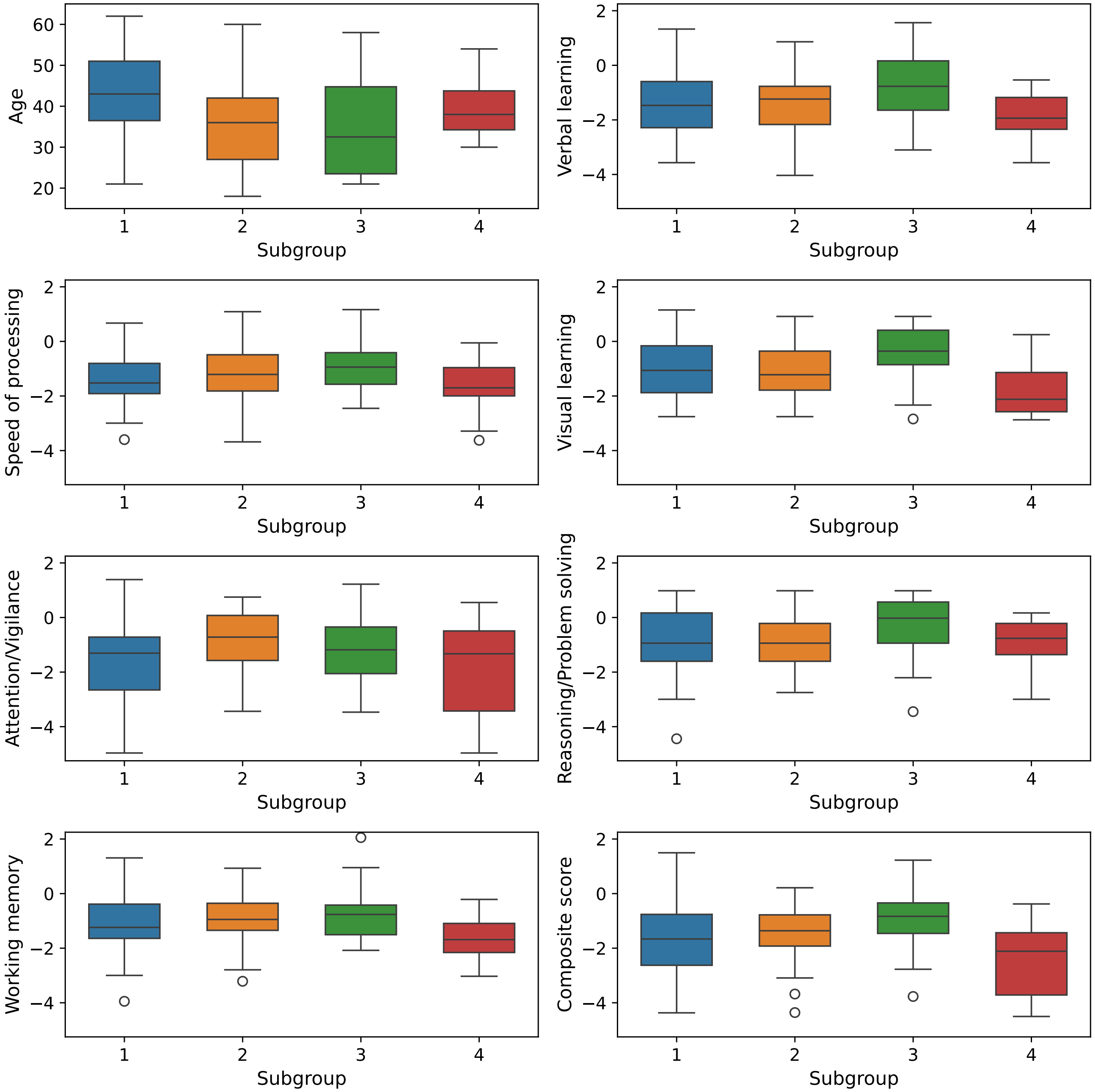

Figure S17: **Subject measure distributions for SZ subgroups (2D latent representations).** Each box plot shows the distribution of each subject measure for each SZ subgroup.

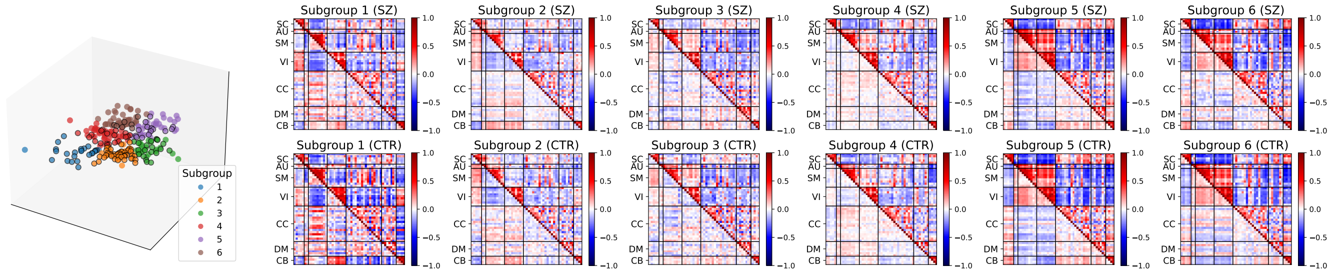

Figure S18: **Subgroups based on 3D latent representations.** In the scatter plot, SZ patients are highlighted in black circles, while controls are shown in their cluster colors. The average sFNC pattern is shown for each of the four k-means subgroups (clusters) and for each of the two diagnostic groups (SZ patients and controls). Subgroups were sorted by the increasing proportion of controls among all subjects.

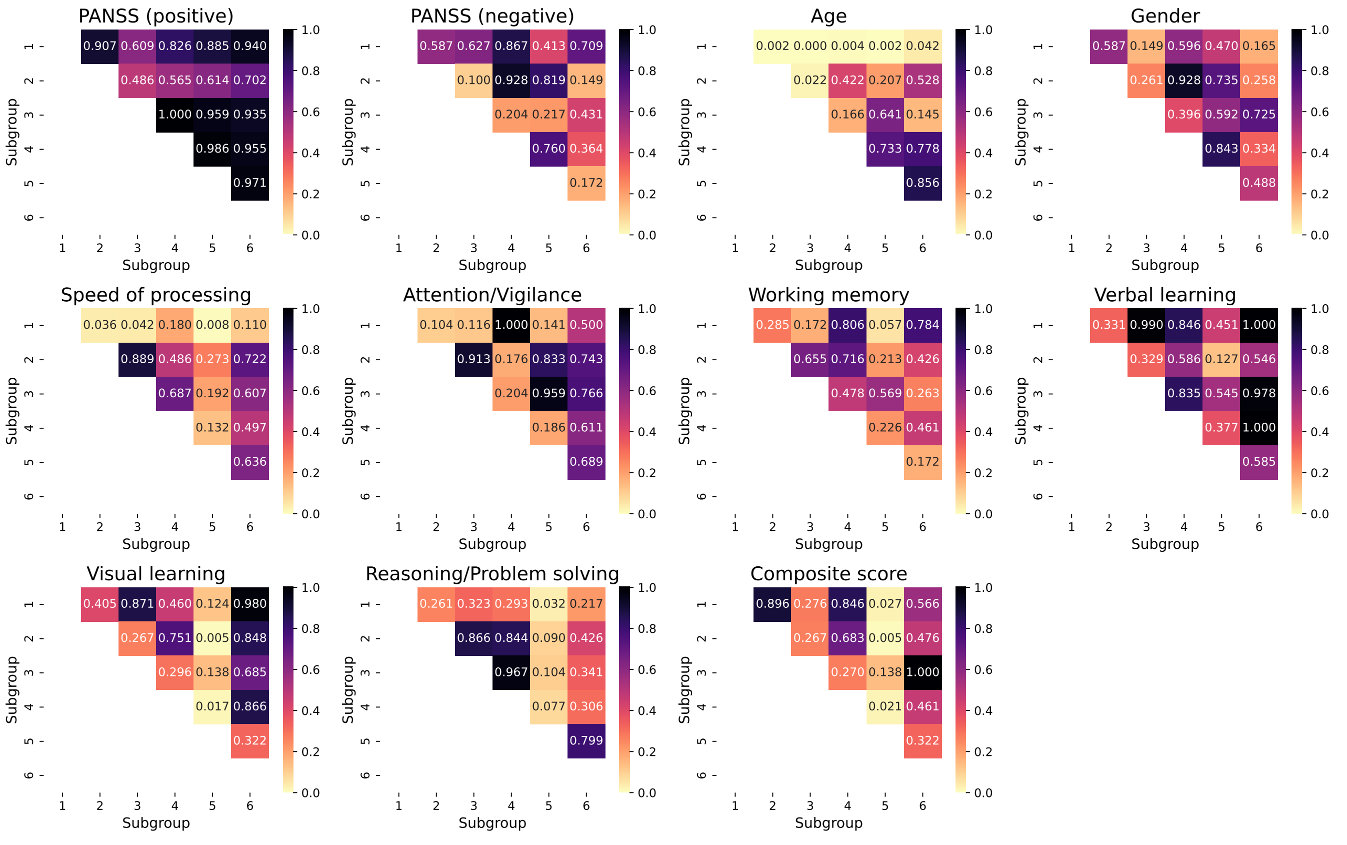

Figure S19: **Statistical significance of each score type between two subgroups of 3D latent representations in the patient group.** The heatmap shows p-values for each score type between all pairs of subgroups (Mann–Whitney U test). Fifteen cluster pairs exhibited statistically significant differences ($p < 0.05$).

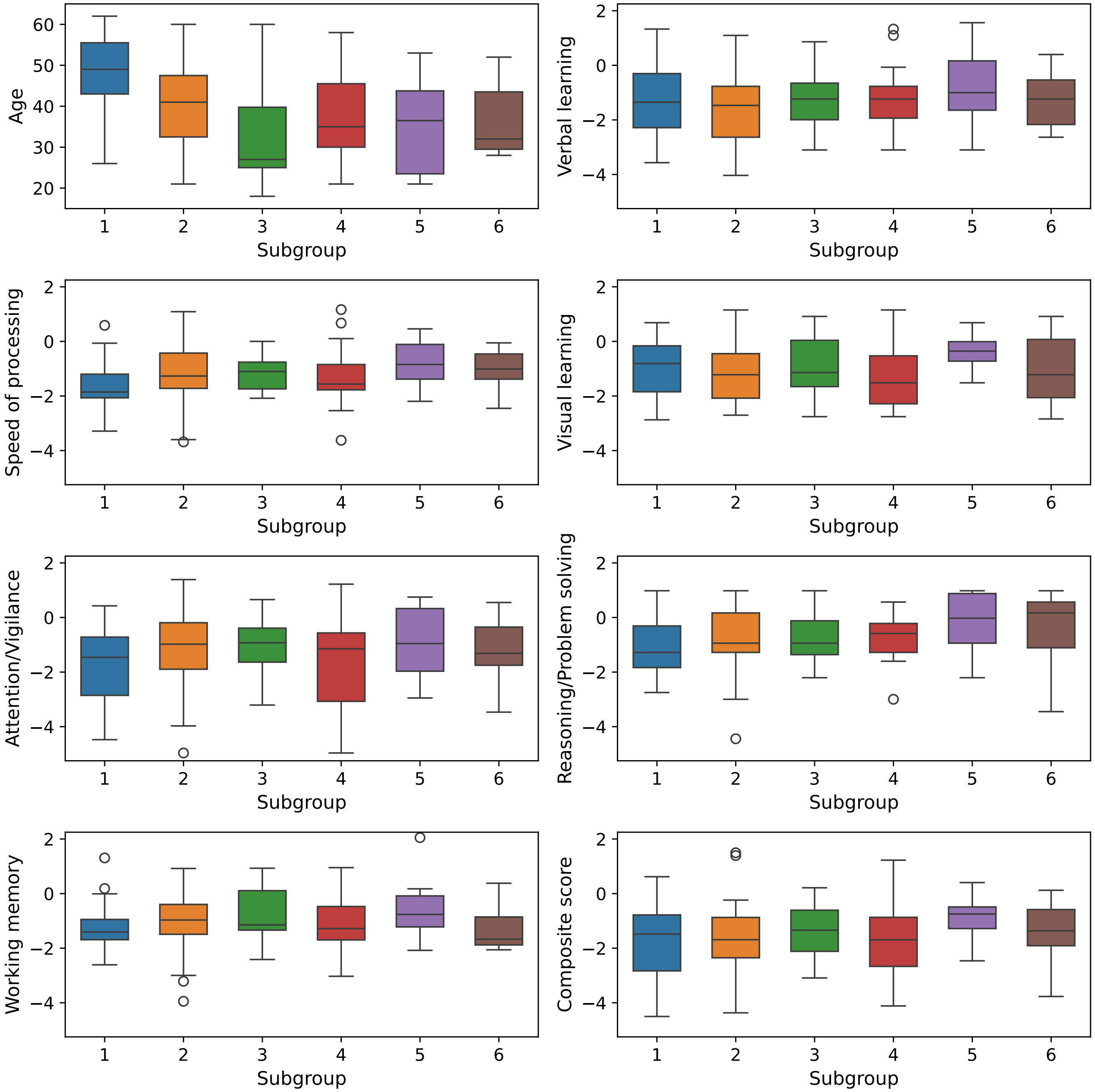

Figure S20: **Subject measure distributions for SZ subgroups (3D latent representations).** Each box plot shows the distribution of each subject measure for each SZ subgroup.

Table S6: **Pairwise subgroup differences in subject measures and functional network connectivity (2D latent representations).** Each row lists a subgroup pair, significantly different subject measures ($p < 0.05$, Mann–Whitney U test), and corresponding functional network connectivity patterns ($p < 0.05$, Wilcoxon signed-rank test with Bonferroni correction).

Subgroup pairs	Subject measures	Functional network connectivity
1, 2	Age<br>Attention	SC-SM, AU-CC, SM-VI, SM-DM, CC-CB ($p < 0.0001$);<br>AU-SM, SM-CC ($p < 0.001$);<br>SC-CB, VI-CB, DM-CB ($p < 0.01$);<br>SC-VI, AU-DM, CB-CB ($p < 0.05$)
1, 3	Age<br>Visual learning<br>Composite score	SC-SM, SC-VI, SC-CB, SM-SM, SM-VI,<br>SM-CC, SM-DM, SM-CB, VI-VI, VI-CC,<br>VI-CB, CC-CB, DM-CB ($p < 0.0001$);<br>AU-SM, AU-VI, AU-CC ($p < 0.001$);<br>SC-SC ($p < 0.01$);<br>SC-AU, SC-CC, CC-CC ($p < 0.05$)
1, 4	Visual learning	SC-SM, SC-VI, SC-CB, SM-VI, SM-CB,<br>VI-VI, VI-CB ($p < 0.0001$);<br>CC-CC, CB-CB ($p < 0.05$)
2, 3	Visual learning<br>Reasoning	SC-SM, SC-VI, SC-CB, SM-SM, SM-VI,<br>SM-CB, VI-VI, VI-CC, VI-CB, CC-CC ($p < 0.0001$);<br>SC-CC, AU-VI ($p < 0.001$);<br>SC-SC, AU-SM, SM-CC, CC-CB ($p < 0.01$);<br>SC-AU, CB-CB ($p < 0.05$)
2, 4	Working memory<br>Visual learning	SC-SM, SC-VI, SM-VI, SM-DM, SM-CB,<br>VI-VI, VI-CB, CC-CB ($p < 0.0001$);<br>AU-CC, SM-CC ($p < 0.001$);<br>SC-CB, SM-SM, CC-CC, DM-DM ($p < 0.01$);<br>SC-SC, AU-DM, CB-CB ($p < 0.05$)
3, 4	Speed of processing<br>Working memory<br>Verbal learning<br>Visual learning<br>Composite score	SC-SM, SC-VI, SC-CB, AU-CC, SM-SM,<br>SM-VI, SM-CC, SM-DM, SM-CB, VI-VI,<br>VI-CC, VI-CB, CC-CB, DM-CB ($p < 0.0001$);<br>SC-CC, AU-SM, AU-VI, DM-DM ($p < 0.001$);<br>AU-DM ($p < 0.01$);<br>SC-SC, SC-AU, VI-DM, CB-CB ($p < 0.05$)

Table S7: **Pairwise subgroup differences in subject measures and functional network connectivity (3D latent representations).** Each row lists a subgroup pair, significantly different subject measures ($p < 0.05$, Mann–Whitney U test), and corresponding functional network connectivity patterns ($p < 0.05$, Wilcoxon signed-rank test with Bonferroni correction).

Subgroup pairs	Subject measures	Functional network connectivity
1, 2	Age<br>Speed of processing	SC-SM, SC-VI, AU-CC, SM-VI, SM-CB, CC-CB ($p < 0.0001$);<br>AU-SM, VI-VI ($p < 0.001$);<br>SM-CC, SM-DM ($p < 0.01$);<br>SM-SM, CB-CB ($p < 0.05$)
1, 3	Age<br>Speed of processing	SC-SM, SC-VI, AU-CC, SM-VI, SM-DM, VI-CB,<br>CC-CB ($p < 0.0001$);<br>AU-SM, SM-CC ($p < 0.001$);<br>SC-CB, DM-DM, DM-CB ($p < 0.01$);<br>AU-DM, CB-CB ($p < 0.05$)
1, 4	Age	SC-VI, SM-VI, SM-CB, VI-VI, VI-CB, CC-CB ($p < 0.0001$);<br>AU-SM, DM-CB ($p < 0.001$);<br>SC-SC, SC-CB ($p < 0.01$);<br>AU-VI, SM-DM, CB-CB ($p < 0.05$)
1, 5	Age<br>Speed of processing<br>Reasoning<br>Composite score	SC-VI, SC-CB, AU-CC, SM-VI, SM-CC, SM-DM,<br>SM-CB, VI-VI, VI-CC, VI-CB, CC-CB, DM-CB ($p < 0.0001$);<br>AU-SM, AU-VI ($p < 0.001$);<br>SC-SC, SC-SM ($p < 0.01$);<br>SC-AU, CB-CB ($p < 0.05$)
1, 6	Age	SC-SM, SC-VI, SC-CB, SM-VI, SM-CC, SM-CB,<br>VI-VI, VI-CB, CC-CB, DM-CB ($p < 0.0001$);<br>AU-SM, AU-VI ($p < 0.001$);<br>SC-SC, VI-CC ($p < 0.01$)
2, 3	Age	SC-VI, SC-CB, SM-VI, SM-DM, SM-CB, VI-VI,<br>VI-DM, VI-CB, CC-CB, DM-DM, DM-CB ($p < 0.0001$);<br>AU-SM, AU-VI, SM-CC ($p < 0.001$);<br>SC-SC, SC-DM, AU-DM ($p < 0.01$);<br>SC-AU, SC-SM, CB-CB ($p < 0.05$)
2, 5	Visual learning<br>Composite score	SC-SM, SC-VI, SC-CB, SM-SM, SM-VI, SM-CC,<br>SM-CB, VI-VI, VI-CC, VI-CB, CC-CC, CC-CB ($p < 0.0001$);<br>SC-CC, AU-SM, AU-VI, AU-CC, DM-CB ($p < 0.001$);<br>SC-SC, SM-DM ($p < 0.01$);<br>SC-AU, SC-DM, CB-CB ($p < 0.05$)
4, 5	Visual learning<br>Composite score	SC-SM, SC-VI, SC-CC, SC-CB, AU-CC, SM-SM,<br>SM-VI, SM-CC, SM-DM, SM-CB, VI-VI, VI-CC,<br>VI-CB, CC-CB ($p < 0.0001$);<br>AU-SM, AU-VI, DM-CB ($p < 0.001$);<br>AU-DM ($p < 0.01$);<br>SC-AU, SC-DM, VI-DM, DM-DM ($p < 0.05$)

12 sFNC interpolation along cognitive score trajectories

To examine how sFNC patterns vary in relation to cognitive performance, we divided subjects in the FBIRN dataset into two groups based on the median cognitive score: a lower-score group (scores ≤ median) and a higher-score group (scores > median). Within each group, we estimated the cluster distribution in the latent space using kernel density estimation and defined the cluster centroid as the point of maximum density. For each cognitive measure, we constructed a trajectory connecting the centroids of the higher- and lower-score clusters in the latent space. Finally, we generated one representative sFNC matrix from each cluster centroid to characterize the connectivity patterns associated with cognitive performance. Most cognitive scores were moderately to strongly correlated with each other (Figure S21). Therefore, the resulting clusters and trajectories were largely similar across different cognitive measures (Figure S22).

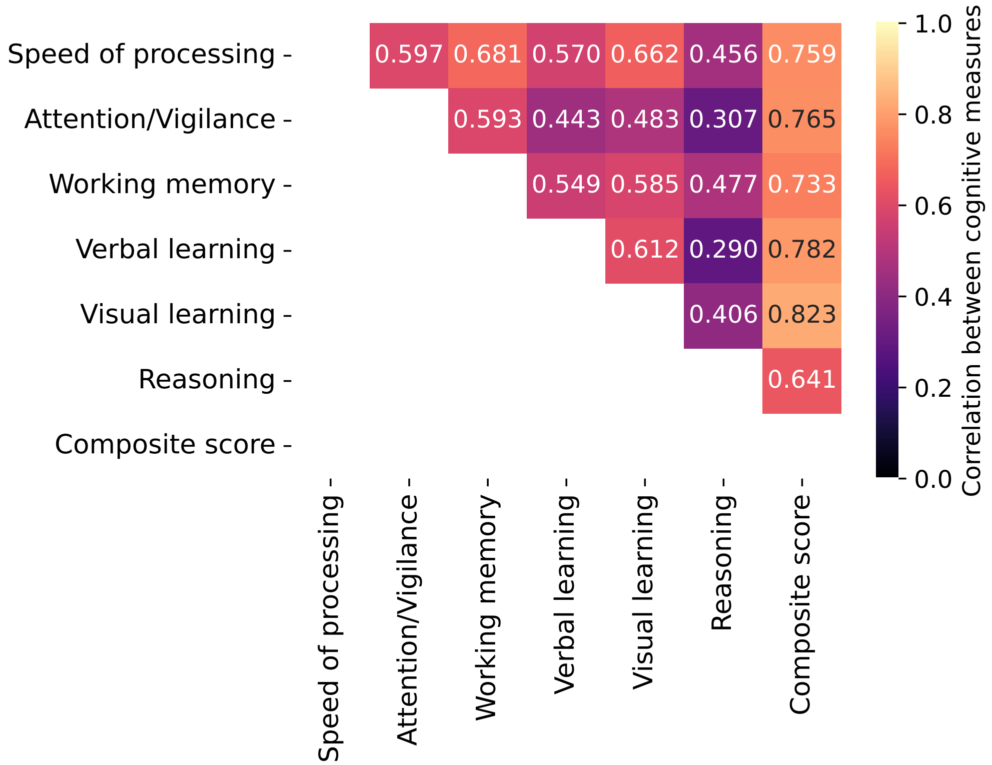

Figure S21: **Correlations between cognitive measures.** Cognitive scores exhibited varying degrees of similarity, with correlation coefficients ranging from 0.290 to 0.823. The composite score was strongly correlated with the other scores.

As shown in Figure S22, the higher- and lower-score clusters occupied highly consistent spatial locations in the latent space, with the lower-score cluster located in the upper-left region and the higher-score cluster in the lower-right region. For each cluster, we generated one representative sFNC matrix by

sampling from the cluster centroid. For each group, we also computed the average sFNC matrix across the original sFNC matrices. The generated sFNC matrices were highly similar to the original ones, with correlations ranging from 0.926 to 0.990 (Figure S23).

Between two cognitive score groups, we observed consistent sFNC changes associated with cognitive performance. As cognitive performance decreased from group 1 to group 2, negative correlations were significantly attenuated in the SC–SM, SC–VI, and SM–CB domains ($p < 0.0001$, Wilcoxon signed-rank test with Bonferroni correction). In addition, positive correlations shifted to negative in the SC–CB and SM–VI domains ($p < 0.0001$), while negative correlations shifted to positive in the VI–CB domain ($p < 0.0001$) and the SC–AU domain ($p < 0.05$). Moreover, positive correlations within the SM, VI, and CC domains were significantly reduced ($p < 0.0001$), as were those within the SC domain and the AU–SM domain ($p < 0.01$), and within the CB domain ($p < 0.05$).

Several connectivity changes were specific to individual cognitive domains. For attention, working memory, and reasoning, positive correlations changed to negative in the AU–VI domain ($p < 0.05$, $p < 0.05$, $p < 0.01$, respectively). Moreover, negative correlations in the VI–CC domain were significantly weaker in the lower-score group for working memory ($p < 0.001$) and reasoning ($p < 0.0001$).

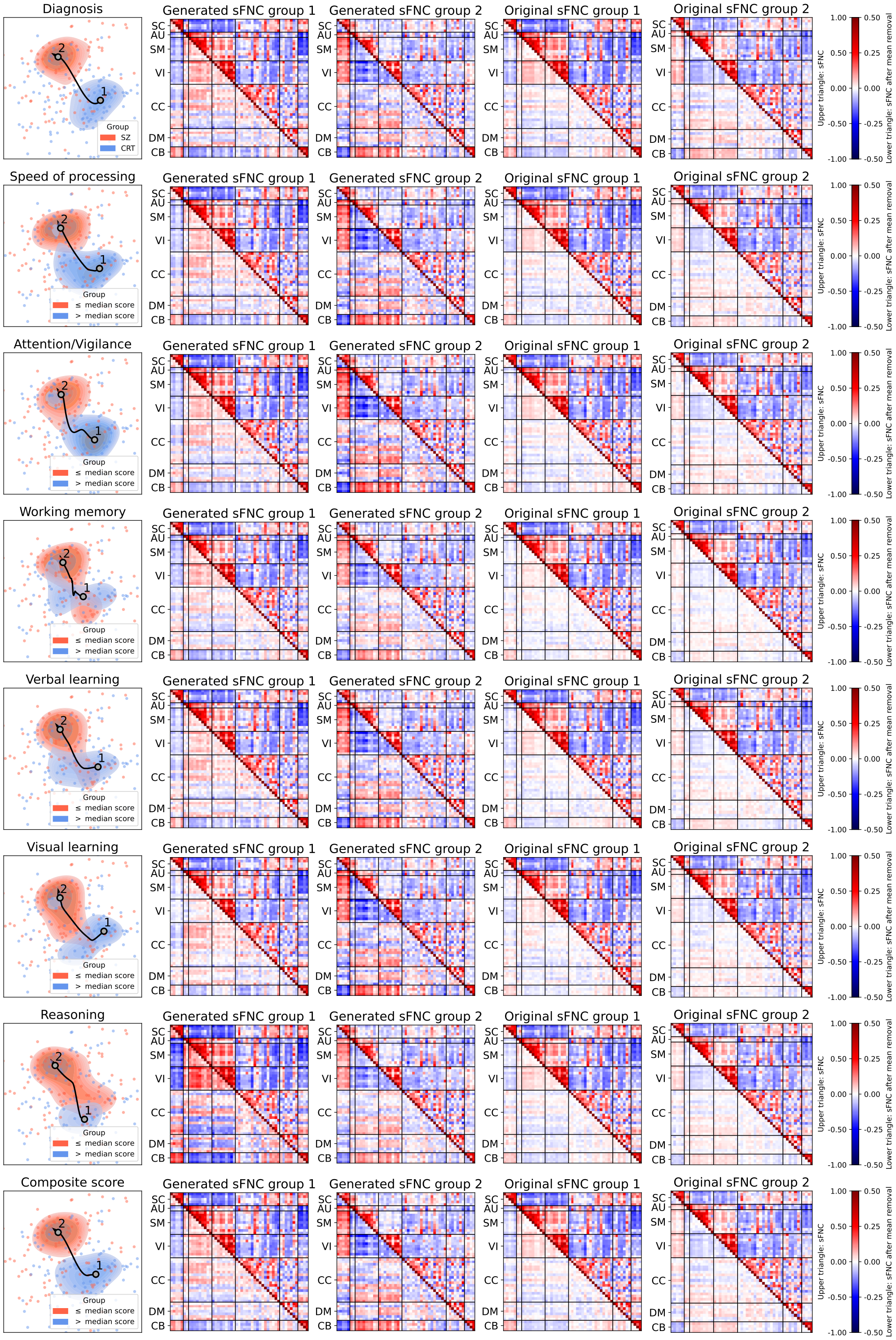

Figure S22: **Interpolation along a trajectory derived from two clusters defined by cognitive scores.** In each row, the scatter plot shows latent features colored by cognitive scores (red: ≤ median score; blue: > median score) and overlaid with contour plots of two clusters connected by a trajectory. For each group, we generated one representative sFNC matrix by sampling from the cluster centroid and computed the average sFNC matrix across the original sFNC matrices.

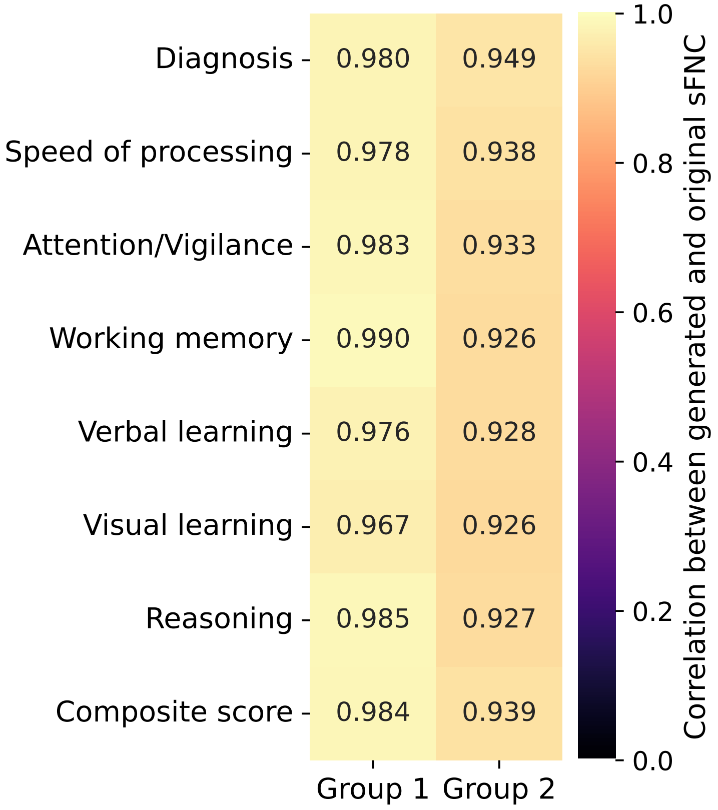

Figure S23: **Correlations between generated and original sFNC.** For each group, the generated sFNC matrices closely matched the corresponding original sFNC matrices.

13 k-means clustering

The k-means clustering algorithm was applied separately to the generated and original dFNC data in each dataset to identify dynamic states by searching the number of states k from 2 to 9. The optimal number of states was then identified using the elbow criterion based on the L_2 loss, defined as the mean Euclidean distance between the samples and their respective centroids. The elbow point occurred at $k = 5$ in most cases (Figure S24a,b,c). Thus, $k = 5$ was selected as the optimal number of states for both datasets.

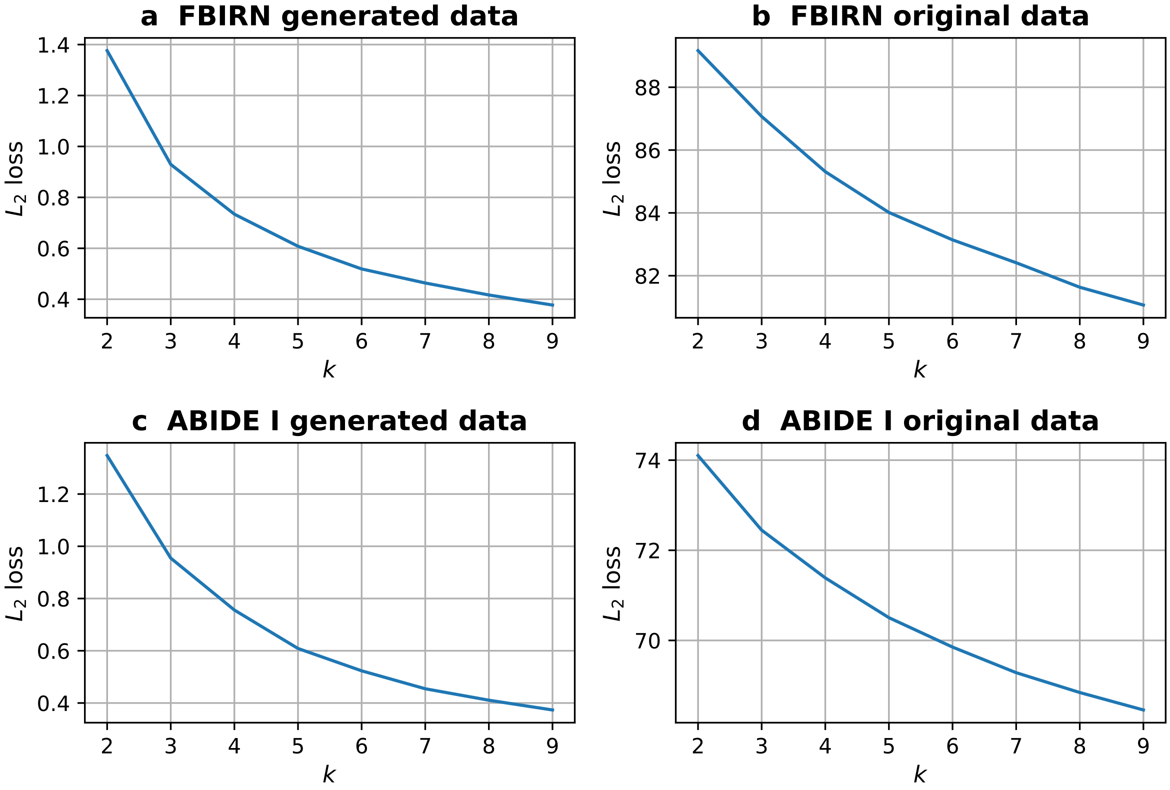

Figure S24: k**-means clustering** L_2 **loss with respect to different** k **values.** (a) FBIRN generated data. (b) FBIRN original data. (c) ABIDE I generated data. (d) ABIDE I original data.

References

Bishop, C. M., & Bishop, H. (2023). *Deep learning: Foundations and concepts* (1st ed.). Springer Nature. https://doi.org/10.1007/978-3-031-45468-4

Kay, S. R., Fiszbein, A., & Opler, L. A. (1987). The positive and negative syndrome scale (panss) for schizophrenia. *Schizophrenia Bulletin*, *13*(2), 261–276. https://doi.org/10.1093/schbul/13.2.261

Khemakhem, I., Kingma, D., Monti, R., & Hyvarinen, A. (2020). Variational autoencoders and nonlinear ica: A unifying framework. In S. Chiappa & R. Calandra (Eds.), *Proceedings of the twenty third international conference on artificial intelligence and statistics* (pp. 2207–2217, Vol. 108). PMLR. https://proceedings.mlr.press/v108/khemakhem20a.html

Kingma, D. P., & Welling, M. (2014). Auto-encoding variational bayes. In Y. Bengio & Y. LeCun (Eds.), *2nd international conference on learning representations, ICLR 2014, banff, ab, canada, april 14-16, 2014, conference track proceedings*. http://arxiv.org/abs/1312.6114

Lopez-Paz, D., Hennig, P., & Schölkopf, B. (2013). The randomized dependence coefficient. In C. Burges, L. Bottou, M. Welling, Z. Ghahramani, & K. Weinberger (Eds.), *Advances in neural information processing systems* (Vol. 26). Curran Associates, Inc. https://proceedings.neurips.cc/paper_files/paper/2013/file/aab3238922bcc25a6f606eb525ffdc56-Paper.pdf

Lord, C., Risi, S., Lambrecht, L., Cook Jr, E. H., Leventhal, B. L., DiLavore, P. C., Pickles, A., & Rutter, M. (2000). The autism diagnostic observation schedule—generic: A standard measure of social and communication deficits associated with the spectrum of autism. *Journal of Autism and Developmental Disorders*, *30*(3), 205–223. https://doi.org/10.1023/A:1005592401947

Maas, A. L., Hannun, A. Y., & Ng, A. Y. (2013). Rectifier nonlinearities improve neural network acoustic models. *Proceedings of the 30th International Conference on Machine Learning (ICML)*, *28*. https://ai.stanford.edu/~amaas/papers/relu_hybrid_icml2013_final.pdf

Tiao, L. C. (2017). A Tutorial on Variational Autoencoders with a Concise Keras Implementation. *tiao.io*. https://tiao.io/post/tutorial-on-variational-autoencoders-with-a-concise-keras-implementation/

Tipping, M. E., & Bishop, C. M. (1999). Probabilistic principal component analysis. *Journal of the Royal Statistical Society: Series B (Statistical Methodology)*, *61*(3), 611–622. https://doi.org/10.1111/1467-9868.00196

van Erp, T. G., Preda, A., Turner, J. A., Callahan, S., Calhoun, V. D., Bustillo, J. R., Lim, K. O., Mueller, B., Brown, G. G., Vaidya, J. G., McEwen, S., Belger, A., Voyvodic, J., Mathalon, D. H., Nguyen, D., Ford, J. M., & Potkin, S. G. (2015). Neuropsychological profile in adult schizophrenia measured with the cminds. *Psychiatry Research*, *230*(3), 826–834. https://doi.org/10.1016/j.psychres.2015.10.028
